# Global and local trajectories of infant gut microbiota development in Bangladesh across the COVID-19 pandemic

**DOI:** 10.1128/msystems.00101-26

**Published:** 2026-09-01

**Authors:** Maria Ioanna Papadaki, Gwen Falony, Sara Vieira-Silva, Raul Yhossef Tito, Leen Rymenans, Jill Swinnen, Lila Close, Jara Wagemans, Shafina Jahan, Muntasir Alam, Kamrun Nahar, Mustafizur Rahman, Jeroen Raes, Jelle Matthijnssens

**Affiliations:** 1 KU Leuven, Department of Microbiology, Immunology and Transplantation, Rega Institute, Laboratory of Clinical and Epidemiological Virology 26657 , Leuven, Belgium; 2 Host-Microbe Interactomics, Department Animal Science, Wageningen University & Research 4508, Wageningen, the Netherlands; 3 Systems Biology and Multiomics Research Group, Institute of Experimental and Clinical Research (IREC), Université Catholique de Louvain, UCLouvain, Brussels, Belgium; 4 KU Leuven, Department of Microbiology, Immunology and Transplantation, Rega Institute 26657 , Leuven, Belgium; 5 VIB, Center for Microbiology, Leuven, Belgium; 6 The Akkermansia Company SA, Mont-Saint-Guibert, Belgium; 7 Department of Cell Biology and Physiology, University of Arkansas for Medical Sciences 12215, Little Rock, Arkansas, USA; 8 Infectious Diseases Division, International Centre for Diarrheal Disease Research 56291 , Dhaka, Mohakhali, Bangladesh; Quadram Institute Bioscience, Norwich, Norfol, United Kingdom; Università di Trento, Trento, Italy

**Keywords:** infant, gut microbiota, longitudinal, 16S rRNA, early life, bacteria, COVID-19

## Abstract

The establishment of the gut microbiota in early life is fundamental to lifelong health, yet this process remains poorly explored outside Western and high-income settings. We investigated microbial maturation in a non-Western context, by longitudinally characterizing the gut microbiota of 20 healthy infants from urban Bangladesh (*n* = 984 fecal samples) across the first 2 years of life. Microbiota development followed a three-staged successional pattern characterized by a progressive increase of microbiota diversity, closely resembling patterns described in Western populations. Comparative analysis with a Belgian infant cohort showed broadly conserved maturation dynamics but population-specific differences in core bacteria, including *Segatella*, which was characteristic of Bangladeshi infants. *Segatella*’s role in the developing gut remains unclear, as high abundances were associated with disease in this cohort. Transient disruptions in maturation also coincided with episodes of illness. The second year of this cohort coincided with the onset of the COVID-19 pandemic where differences in the relative abundance of several key taxa were detected. These insights expand our understanding of healthy infant gut microbiota development across populations and emphasize the need to consider sociocultural and environmental factors, including global disruptions, in shaping early-life microbial ecosystems.

The first years of life represent a critical period during which the gut microbiota is established, with lasting implications for long-term health. However, current knowledge of this process is derived largely from studies conducted in Western and high-income populations even though gut microbiota composition is known to vary across geographic locations. In this study, we longitudinally characterized the gut microbiota development in a healthy infant cohort from urban Bangladesh, showing that geographically distinct populations can harbor different microbial communities while following similar developmental patterns. We further observe that illness can temporarily influence microbiota maturation, and that part of the microbiota maturation period in this cohort overlapped with a major societal disruption, the COVID-19 pandemic. Together, these findings illustrate the importance of geographic, cultural, and environmental context when defining healthy gut microbiota trajectories and contribute to a more inclusive, globally representative framework for understanding early-life microbiota development.

## INTRODUCTION

The human gut microbiome has gathered increasing attention over the past decade due to its critical role in health and disease. The gut microbiota is a complex and dynamic ecosystem, including bacteria, viruses, fungi, and protists, and begins its establishment early in life with the infant gut undergoing rapid colonization at birth (1, 2). As the gut microbiota contributes to various physiological processes, including metabolism, immune regulation, and defense against pathogens (1–3), its balanced establishment in infancy is crucial. Understanding the factors shaping its composition and development during this period is, therefore, vital. Microbial imbalance, or dysbiosis, has been linked to a range of conditions (3), from immunological disorders such as allergic asthma to metabolic and inflammatory diseases like diabetes and inflammatory bowel disease (IBD) (4–8).

Gut colonization starts at birth and generally follows a consistent trajectory (1, 2, 9–11). The initial inoculation is characterized by pioneer bacteria, primarily facultative anaerobes such as *Enterobacteriaceae* (11). Breastfeeding promotes dominance of fermentative bacteria such as Bifidobacterium, facilitating the digestion of human milk oligosaccharides (HMOs) (11). As infants transition to solid foods, microbial diversity expands, with key taxa like *Bacteroidota*, *Lachnospiraceae*, and *Ruminococcaceae* emerging to metabolize complex carbohydrates (11, 12). By age 2 to 3, the gut microbiota increasingly resembles an adult-like composition (12, 13).

Numerous factors influence the early gut microbiota development, including host genetics, delivery mode, medication exposure, and environmental factors such as diet and lifestyle (9, 12, 14). Geographical and cultural differences also shape microbial composition. Studies have consistently shown that gut microbiota diversity and structure vary with urbanization, westernization, and lifestyle (9, 15–17). Compared to their urban and Western counterparts, children living in rural or less industrialized environments tend to harbor highly diverse gut microbial communities, enriched in fiber-degrading bacteria such as *Segatella* (formerly *Prevotella *[9, 16–19]).

Despite growing recognition of the gut microbiota’s critical role in human health, research remains heavily skewed toward Western and high-income populations, leaving significant gaps in our understanding of microbial diversity across different geographical and cultural contexts. Although gut microbiota studies across regions began over a decade ago (13), data from non-Western populations remain limited (20), with most work focusing on the adult gut microbiome. This imbalance precludes the acquisition of a more comprehensive perspective on how inheritance, environmental, dietary, and lifestyle factors shape the gut microbiome globally. While extensive research in Western cohorts has explored gut microbiota in health and disease, studies in non-Western settings have largely focused on pathological conditions, such as malnutrition (21, 22), with limited data available on the healthy intestinal microbiota development (23–25). Expanding microbiome research to underrepresented populations in low- and middle-income countries (LMICs) is essential (26) for achieving a more inclusive and globally relevant understanding of gut microbial ecology and its implications for human health.

In this study, we analyzed 16S rRNA data from a densely sampled longitudinal cohort of healthy Bangladeshi children (BBGUT cohort) to investigate early-life gut microbiota dynamics. Specifically, we characterized the development of the gut bacterial community from birth to 2 years of age in an urban infant population from Dhaka, a period that partially overlapped with the COVID-19 pandemic, and explored factors that may influence this process. Finally, to place our findings in a global context, we compared them with a previously studied Belgian infant cohort (BABEL cohort) (10) to explore how these dynamics occur in different geographical settings.

## MATERIALS AND METHODS

### Study design and sample selection

This study was conducted in Kamalapur, an urban area located in southeast Dhaka characterized by high population density and low socioeconomic status. Thirty healthy pregnant women were enrolled at the International Centre for Diarrheal Disease Research, Bangladesh (icddr,b), and their newborns were followed from birth over a 2-year period (children were born between 26 October 2018 and 01 December 2018, and sampling occurred between 26 October 2018 and 24 November 2020). Infant fecal material collection occurred three times/week during the first 6 months, twice/week in the following 6 months, and once/week throughout the second year. Additionally, one fecal sample per mother was collected shortly after birth. Trained field staff conducted household visits for sample collection and transport to the laboratory, during which detailed metadata questionnaires were completed. These questionnaires were designed to collect longitudinal infant-specific metadata, including demographic characteristics (birth mode, birth place, and household composition), medical history (vaccination status, antibiotic/medication exposure, and disease occurrence), and dietary practices (breastfeeding, formula feeding, and solid food introduction) (Table S1). Further details on metadata collection are provided in Supplemental methods.

Due to incomplete sampling and missing metadata, only 20 of the 30 enrolled infants were included in this study. In total, 1,002 samples were selected for this study, from which 18 were maternal and 984 originated from 20 infants. To explore the longitudinal development of the healthy gut microbiota, we selected approximately 33 samples per infant at predefined time points without reports for antibiotic intake or gastrointestinal disease (~21 samples during the first year, ~12 during the second). For this purpose, 486 (out of 984) samples were selected (i.e., samples representing healthy time points or HTP samples). To assess the effect of external events on the early gut microbiota development, we selected 20–30 additional samples per infant associated with specific life events during the first year of life. For this, 498 (out of 984) *ad hoc* collected samples (i.e., collected on an *ad hoc* basis, ADHC samples) were selected (Fig. S2). For the comparative analysis with the Belgian infant population, we included 265 samples from the BABEL cohort (10) consisting of 7 infants. A more detailed description of the study design and sample selection can be found in Supplemental methods.

### Fecal microbiota profiling and moisture content measurement

Frozen samples were aliquoted (150–200 mg) at the Rega Institute (Belgium) without thawing and further maintained at −80°C until DNA extraction. Bacterial DNA extraction followed a modification of a published protocol (27), and 16S rRNA amplification and sequencing were performed as previously described (28). Sequencing was performed on 7 runs using the Illumina MiSeq platform (2 × 250 paired-end reads). Full methodological details are provided in Supplemental methods.

Maternal fecal samples (*n* = 18) were further processed to estimate their moisture percentage by freeze drying using the Alpha 1–4 LSCplus (Martin Christ) freeze dryer and comparing weights before and after lyophilization.

### Sequencing data preprocessing

Following demultiplexing with sdm in the LotuS pipeline (29), with no mismatches permitted, the fastq sequences were analyzed separately for each sample using the DADA2 pipeline (v.1.6) (30). Primer sequences and the first 10 subsequent nucleotides were trimmed. Taxonomy was assigned using the formatted RDP (Ribosomal Database Project) training set "rdp_train_set_19” (31) after merging paired sequences and eliminating chimeras. The decontam R package with the prevalence method was used for identification and removal of contaminant sequences (32). In total, 224 bacterial and 2 archaeal amplicon sequence variants (ASVs) were identified in our data set. Samples were rarified to 15,086 reads, and sequences with a relative abundance smaller than 0.0001 across all samples were removed. To ensure a meaningful comparative analysis of this cohort with the previously studied Belgian cohort (10), the Belgian data were re-processed (7 infants with samples up to 365 days of age) using the approach described here. In our updated analysis, the Belgian cohort exhibited two main successional stages of microbiota development in contrast to the three stages reported in the original publication (10). The reduced number observed here likely reflects comparable underlying dynamics, with differences attributed, among other factors, to changes in data processing and taxonomical updates in the available databases since the original publication (Fig. S4).

### Statistical analyses

All statistical analyses and visualization were conducted in R (http://www.R-project.org, v4.3.2), using the following packages: ggplot2 (33) (v3.4.4), vegan (34) (v2.6-4), phyloseq (35) (v1.42.0), microbiome (36) (v1.20.0), and microViz (37) (v0.11.0). To analyze group differences and temporal trends in alpha diversity, linear mixed-effects models were used with the lme4 (38) (v1.1-37) and lsmeans (39) (v2.30-2) R packages. These models account for the non-independence of repeated measurements from the same infants by including subject-level random intercepts. *P*-values were computed using likelihood ratio tests for overall effects and Kenward-Roger adjusted Wald tests for pairwise group comparisons. To compare median differences between independent or paired groups of continuous variables, we used the Wilcoxon rank-sum (or Mann-Whitney *U*) and Wilcoxon signed-rank tests for two groups. To evaluate the association between genera prevalence patterns across the Bangladeshi and the Belgian infant cohorts, a Spearman rank correlation analysis was computed. Differences in microbial community composition (beta diversity) between cohorts were assessed using permutational multivariate analysis of variance (PERMANOVA) with the adonis2 function in the vegan R package (Bray-Curtis dissimilarity, 999 permutations). Differences in *Segatella* prevalence between sample groups across gut microbiota maturation stages were assessed using Fisher’s exact tests while Pearson’s chi-squared tests were conducted on contingency tables that were created to assess the association between different covariates and (i) the occurrence of maturation setbacks and (ii) the presence of samples with high *Segatella* abundance. Where applicable, multiple testing adjustments were made using the Benjamini-Hochberg method, with a false discovery rate (FDR) threshold set at 0.05.

Additional statistical analyses were performed for (i) the identification of bacterial communities (Dirichlet multinomial mixture modeling [DMM]), (ii) determination of maturation setbacks, (iii) order of appearance of the most abundant bacterial genera, (iv) diversity metrics and multivariate analysis of metadata effects on the microbiota, and (v) analysis of samples with high *Segatella* abundance. Detailed descriptions of these analyses are provided in Supplemental methods.

## RESULTS

### The healthy Bangladeshi infant gut undergoes bacterial colonization in three distinct developmental stages

**Fig 1:**
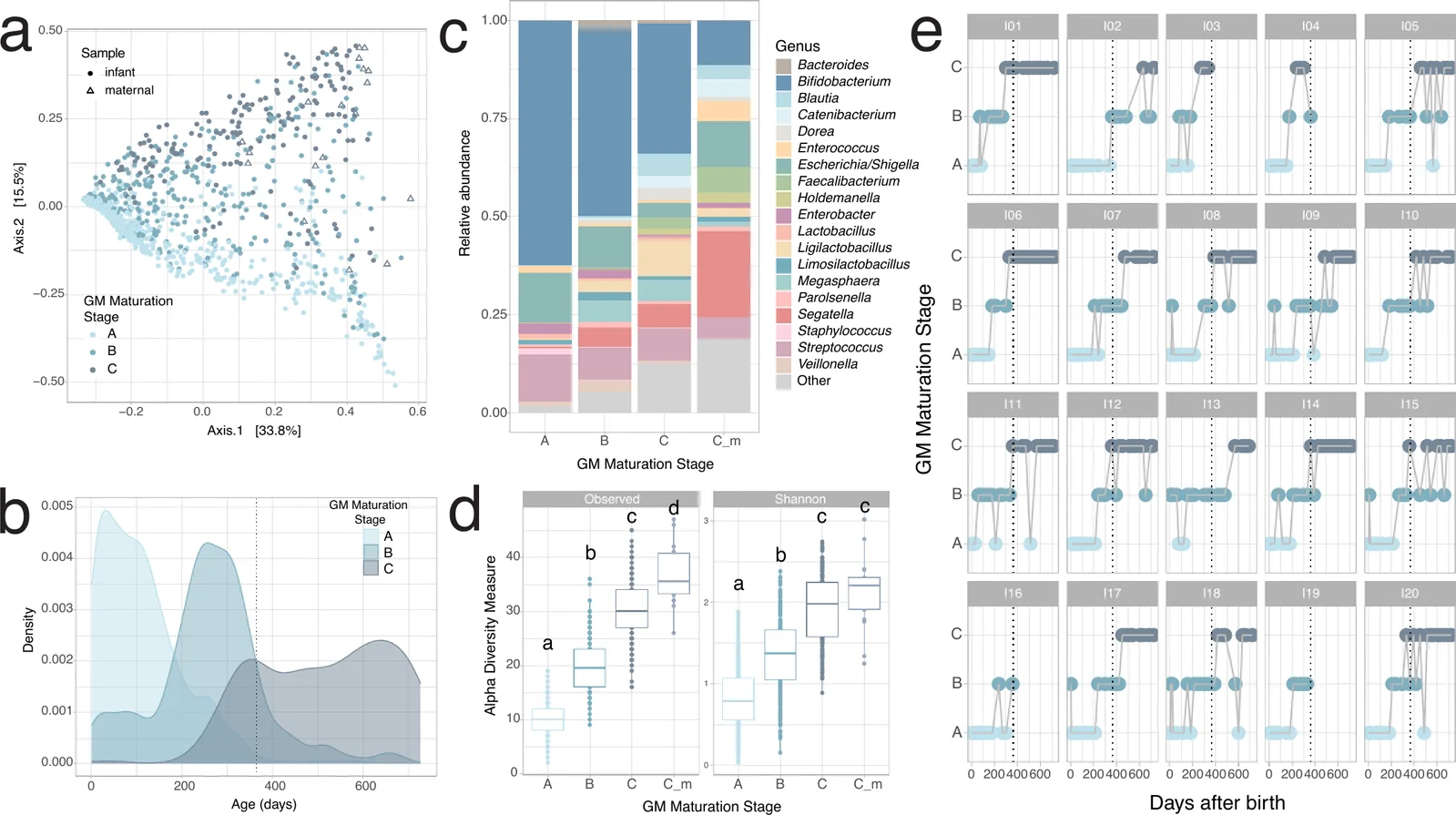
(**a**) Principal coordinate analysis (PCoA) based on Bray–Curtis dissimilarity showing the clustering of all infant (*n* = 984) and maternal samples (*n* = 18). Samples are colored according to gut microbiota maturation stage, with circles representing infant samples and triangles representing maternal samples. (**b**) Density plot illustrating the distribution of sample age (in days) across GMM stages A, B, and C. The vertical dashed line indicates the separation between year 1 and year 2 at day 365. (**c**) Relative abundance of the 10 most abundant genera within each maturation stage describing the Bangladeshi cohort (A [*n* = 499], B [*n* = 312], C [*n* = 173], C_m [*n* = 18]). For visualization purposes, infant and maternal samples assigned to stage C are displayed separately as C and C_m, respectively. (**d**) Alpha diversity measures (observed genus richness and Shannon diversity), across gut microbiota maturation stages. Alpha diversity increased progressively from stage A to C. Differences in alpha diversity measures between the three stages were analyzed using linear mixed-effects models with infant-specific random effects to account for repeated measures from the same infants. Significant increases were observed from stage A to B (*P* < 0.001) and from B to C (*P* < 0.001) for both observed richness and Shannon diversity. Observed richness within maturation stage C was found to be higher in mothers compared to infants (*P* < 0.001); but Shannon diversity was not significantly different (*P* = 0.371). (**e**) Stepwise progression of gut microbiota maturation stages per infant. Identified maturation stages, determined using the DMM analysis at the genus level on the combined infant and maternal samples (*n* = 1,002), are represented in three distinct colors along the vertical axis. Here, only HTP infant samples (*n* = 486) are shown. The horizontal axis represents age in days (0–2 years), with dashed lines indicating the separation between the first and second year of life. Solid lines are used to connect the samples of each individual for visualization purposes. Only 16/20 selected infants have samples spanning both years.

Using 16S rRNA data, we explored gut microbiota maturation dynamics in 20 Bangladeshi infants during the first 2 years of life. We first assessed whether the data set clustered into distinct community types, using the Dirichlet multinomial mixture (DMM) modeling approach. This analysis revealed the presence of three compositionally distinct clusters in which Bangladeshi infant samples were structured throughout the first two years of life, designated as clusters A, B, and C (Fig. 1a). Following the definition previously described by Beller and colleagues (10), these clusters are also referred to as gut microbiota maturation (GMM) stages. Specifically, during the first months of life, infant samples predominantly clustered within stage A, gradually transitioning to stage B, and ultimately reaching stage C around the beginning of the second year of life (Fig. 1b and Fig. S6a). When analyzed alongside the infant cohort, maternal samples mainly clustered within stage C (Fig. 1a), supporting the notion that this transition represents gut microbiota maturation toward an adult-like profile.

At the genus level, our analysis showed an overall increase in bacterial diversity across maturation stages and time (Fig. 1c and d and Fig. S6b). *Bifidobacterium* dominated the Bangladeshi infant gut throughout the first two years of life. Facultative anaerobes, such as *Escherichia*/*Shigella* and *Streptococcus,* were also highly abundant in maturation stage A, but unlike *Bifidobacterium*, these taxa declined in stages B and C (Fig. 1c). A similar trend was observed in Belgian infants (10), suggesting that these genera may be indicative of a conserved early-life gut environment, with higher oxygen availability (40). Overall, the Bangladeshi infant gut is initially colonized by opportunistic bacteria and facultative anaerobes, which is consistent with the current understanding of early-life microbial colonization (12, 41–43). Concurrently, we observed the early establishment of *Bifidobacterium* which could be attributed to breastfeeding and the fact that most infants were vaginally delivered (*n* = 14; Table S1) (12, 44, 45). Introduction of solid foods in infancy leads to an increase in bacterial richness and the gradual emergence of more obligate anaerobes, such as *Dorea* and *Faecalibacterium*, capable of metabolizing structurally diverse dietary compounds. Notably, *Bacteroides* had a relatively low abundance in Bangladeshi infants and was nearly absent in their mothers (1/18 individuals with a relative *Bacteroides* abundance of >0.5%; Fig. 1c and Fig. S7), a key difference from Western populations.

Bangladesh is a country with high breastfeeding rates (46, 47), and in this cohort, all infants were breastfed at similar frequencies. Most infants were exclusively breastfed for approximately the first 6 months, after which solid foods were introduced alongside breastfeeding. Prolonged breastfeeding likely contributed to the dominance of *Bifidobacterium* in the gut microbiota across all developmental stages. Although 16S rRNA V4 gene marker sequencing generally lacks species- and strain-level resolution, the most abundant *Bifidobacterium* ASV in our data set was classified as *B. longum* when compared to RDP taxonomy, indicating that at least some of the dominant ASVs correspond to this species, in line with observations from other Bangladeshi infant cohorts (24, 48). As this species is capable of degrading both HMOs and complex polysaccharides when breastfeeding is combined with solid food (24) and given that dietary diversity has been associated with bifidobacteria abundance (49), this may explain the maintained high relative abundance of the genus following the introduction of solid food. This compositional pattern aligns with a recent study reporting that, during early infancy, microbiota in many Asian populations, including Bangladesh, are enriched in *Bifidobacterium*, reflecting early-stage development (50).

Although *Segatella* was initially absent in early life, its relative abundance increased as the gut microbiota matured toward GMM stage C (Fig. 1c), mirroring trends in non-Western and less industrialized infant and adult populations, where species of the *Prevotellaceae* family dominate the gut microbiome (13, 16, 17, 19, 24). Compared to infants, members of the *Prevotellaceae* family were enriched in several maternal fecal samples (15/18 individuals harbored *Segatella* alone or in combination with *Leyella* and/or *Prevotellamassilia*; mean combined relative abundance: 30.5%) (Fig. S7). Analysis of the maternal samples revealed a high water content, averaging 88.6% (Fig. S7). High fecal moisture reflects looser stool consistency, which corresponds to higher Bristol Stool Scale (BSS) scores and can serve as a proxy for faster colonic transit time (51). Elevated stool water content has been associated with the *Prevotella* enterotype (as described by Arumugam et al. [19]) in a Belgian adult cohort (52). This enterotype linked to fiber-rich diets common in non-Western settings (53) is characterized by a microbial community in which *Prevotella* (now *Segatella*) plays a major role in carbohydrate degradation (54). Consumption of non-fermentable fiber increases stool water content and plasticity, contributing to increased water retention in the gut (52, 55).

Alpha diversity differed significantly across GMM maturation stages (linear mixed-effects models (LMM), *P* < 0.001), with a stepwise increase in microbiota richness and diversity from stage A to B (LMM, *P*< 0.001) and from B to C (LMM, *P* < 0.001), supporting a structured, sequential maturation process (Fig. 1d; Table S3).

Despite occasional regressions to earlier maturation stages (Fig. 1e)—potentially due to illness, medication, or other factors—we observed a conserved and structured trajectory of microbiota maturation over time in three distinct GMM stages across infants (Fig. 1e, *n* = 20, Kendall test, Kendall’s corrected *w* = 0.91, and *P* = 9.9e−5). This finding highlights the resilience of the Bangladeshi infant gut microbiome in its ability to maintain a structured and predictable developmental pattern despite occasional regressions.

### Bangladeshi and Belgian infant microbiota show similar developmental patterns with distinct microbial signatures

To investigate gut microbiota maturation patterns across different geographical and lifestyle settings, we compared the gut microbiota maturation patterns of the Bangladeshi infant cohort (BBGUT) with a previously studied Belgian infant cohort (BABEL) (10) considering only samples representing healthy time points for both populations. The Bangladeshi and the Belgian data sets were generated and processed using the same analytical approach for comparability, with analysis limited to the first year of life, as it is the data available for the Belgian cohort.

**Fig 2:**
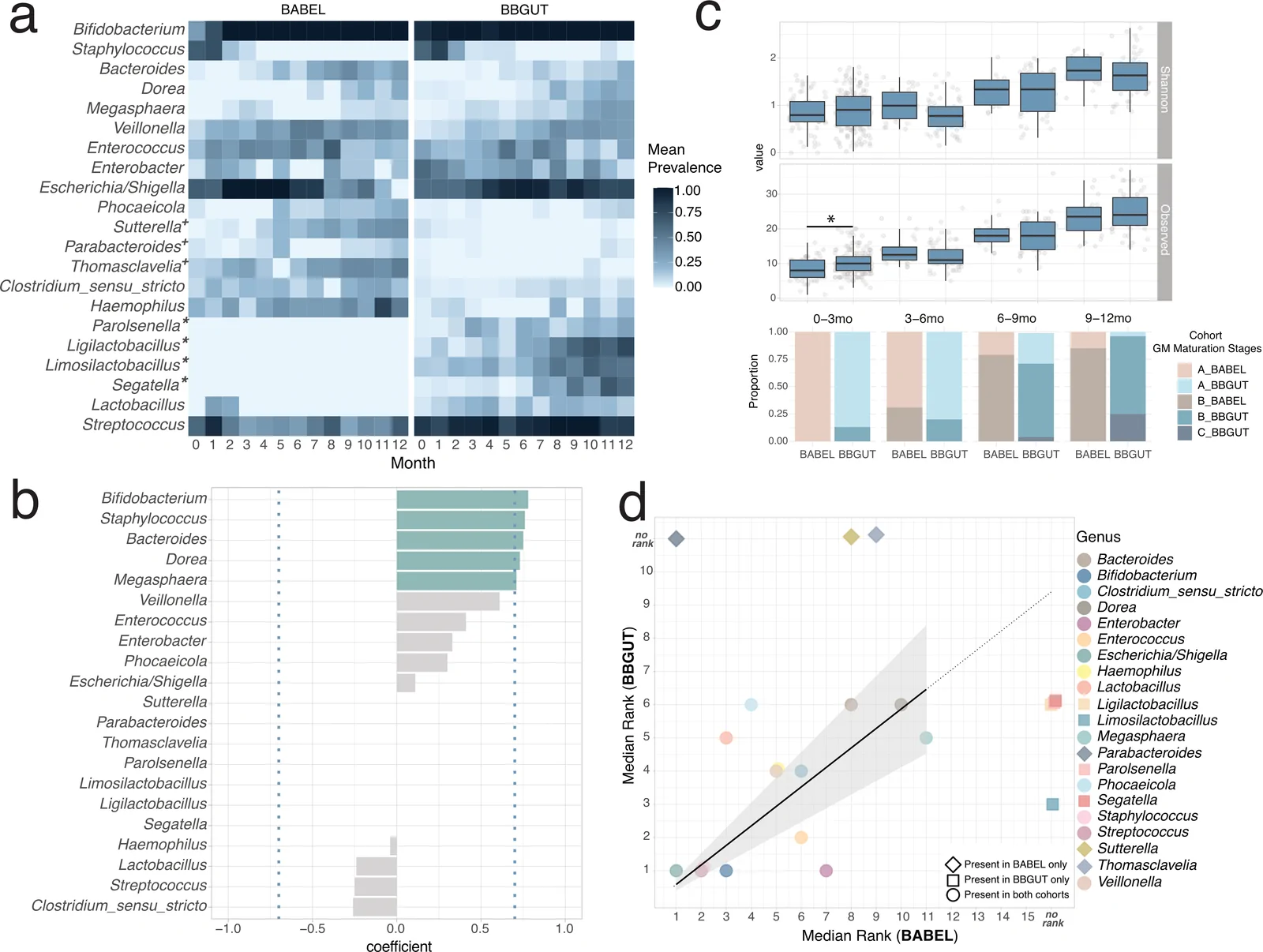
(**a**) Heatmap showing the prevalence of each of the 15 most abundant genera identified across the first year of life of the two cohorts (BABEL *n* = 124, BBGUT *n*= 328). Each cell represents the mean prevalence of a specific genus across all samples of a specific cohort and month of the year. To account for the rapid changes in infant gut composition after birth, the first 10 days of life have been grouped as Month 0. Genera unique to BBGUT are shown with *, while genera unique to BABEL are indicated with +. Here, a genus unique to a specific cohort is defined as one that is present in more than 25% of the infants of each cohort, with a per sample relative abundance detection threshold of 0.5%. (**b**) Spearman correlation analysis of mean genus prevalence between the BBGUT and BABEL cohorts across time. The plot displays the strength of correlations (Spearman’s rho) for genera present in both cohorts, with the genera ordered by correlation strength. Each bar represents the Spearman correlation for a genus, with the teal color indicating statistical significance after correction for multiple testing (*P* < 0.05). Dotted vertical lines at −0.7 and 0.7 indicate thresholds for significant negative and positive correlations. Genera unique to either cohort are represented with a correlation of zero. (**c**) Top: Alpha diversity measures (observed genus richness and Shannon diversity) comparing diversity over time between the two cohorts. When comparing the two cohorts a significant difference in richness was only observed between months 0–3 (Wilcoxon rank-sum test, *P* = 0.003, FDR < 0.005). Bottom: Proportion of GMMs observed in each cohort across age bins (0–3, 3–6, 6–9, 9–12 months). (**d**) Correlation of median genus ranks between the BABEL and BBGUT cohorts. Each point represents a genus, colored according to its taxonomic classification. Genera present in both cohorts are depicted with circles, while those exclusive to one, the BBGUT or the BABEL, are represented by squares and diamonds respectively. A linear regression (solid black line) was fitted to genera shared between both cohorts, with a forced intercept at (0,0) to visualize the correlation in order of appearance, with a dotted line extending the regression beyond observed data points. Missing genera were assigned the highest rank for visualization. A Kendall rank correlation test showed significant association between the genus ranks in the two cohorts (Kendall’s *w* = 0.79, *P* = 0.016, Spearman rho = 0.58).

First, we examined differences in the prevalence of the most abundant bacterial genera in the two cohorts (Fig. 2a). Our analysis revealed that only about 33% of the most abundant genera (5 out of 15) detected in each cohort exhibited similar prevalence trends over the first year of life in both cohorts, supported by a significant positive correlation in mean prevalence over time (Fig. 2b). While some of these bacteria followed similar patterns in acquisition and persistence in both cohorts during early life, the relative abundance dynamics differed over time for most genera (Fig. S8a and b). Cohort differences included the presence of *Segatella*, found exclusively in Bangladeshi infants (Fig. 2a, BBGUT), supporting the dominance of *Prevotellaceae* members in non-Western regions over *Bacteroides* (13, 53, 56). Certain lactic acid bacteria (*Limosilactobacillus, Ligilactobacillus*) were also exclusive to Bangladesh (Fig. 2a, BBGUT), whereas bacteria taxonomically assigned to *Parabacteroides, Thomasclavelia,* and *Sutterella* genera were predominantly found in Belgium (Fig. 2a, BABEL). Additionally, *Streptococcus* was more prevalent and abundant in Bangladeshi infants (Fig. 2a, BBGUT; Fig. S8a) together with a richer community of *Bacillota* genera, particularly lactic acid bacteria such as *Lactobacillus*. Elevated *Bacillota* diversity has been previously reported in Bangladeshi children and infants (16, 57), aligning with our observations. These differences suggest variations in microbial signatures due to geographical factors and the bio-cultural environment, such as divergent breastfeeding practices (e.g., continuation of breastfeeding alongside solid food in Bangladeshi infants, which was not the case for Belgian infants). Beta diversity analysis further supported the differences in community composition between the two cohorts (permutational multivariate analysis of variance, Adonis test, *R*^2^ = 0.059, *P* = 0.001; Table S4).

We next assessed alpha diversity patterns to determine whether microbiota diversity (genus-level bacterial diversity) evolved similarly in both cohorts and found comparable trajectories over time (Fig. 2c; Table S5). Bacterial GMM stages aligned well with infant age across the first year (Fig. 2c, bottom), progressing from A to A–B in BABEL and from A to A–B to A–B–C in BBGUT. To determine whether these observations resulted from age-binning effects, we performed a month-by-month analysis of alpha diversity (Fig. S9). Differences in alpha diversity were statistically significant only during the first 10 days of life (Shannon diversity and observed richness, Wilcoxon rank-sum tests, adjusted *P* < 0.01) while subsequent time points showed similar patterns, supporting our initial conclusion that alpha diversity follows a consistent trajectory over time.

To further determine if infant gut microbiota development follows a consistent pattern of bacterial colonization in two geographically distinct populations, we analyzed the temporal sequence, or the order of appearance, of the 15 most dominant bacterial genera during the first year of life for both cohorts (Fig. 2d). We defined genera as appearing when their relative abundance exceeded 0.5% and were ranked accordingly for each infant in each cohort (Fig. S10). Individual analysis revealed that the order of appearance was highly consistent across individuals for both BBGUT (*n* = 20, Kendall test, corrected Kendall’s *w* = 0.59, *P* = 9.99e−05) and BABEL cohorts (*n* = 7, Kendall test, corrected Kendall’s *w* = 0.49, *P* = 9.99e−05), consistent with previous reports (10).

Despite cohort-specific compositional differences, we observed a significant positive correlation in the sequence of bacterial appearance during early life when comparing infants from Belgium and Bangladesh (Fig. 2d). These findings suggest a conserved pattern of microbial succession across geographically and culturally distinct infant populations (Kendall test, corrected Kendall’s *w* = 0.79, *P* = 0.016). We found that lower ranking colonizers, mostly facultative anaerobes such as *Pseudomonadota* members, appeared early in life, co-occurring with *Actinomycetota*, and followed by the later appearance of more strictly anaerobic bacteria, such as *Bacillota* (Fig. S10). While the general order of appearance is similar between the two cohorts, we observed notable differences, including a simultaneous early appearance of most genera in the BBGUT cohort vs a more gradual succession over time in BABEL (detailed overview in Fig. S10).

### Disease-related events are associated with maturation setbacks

**Fig 3:**
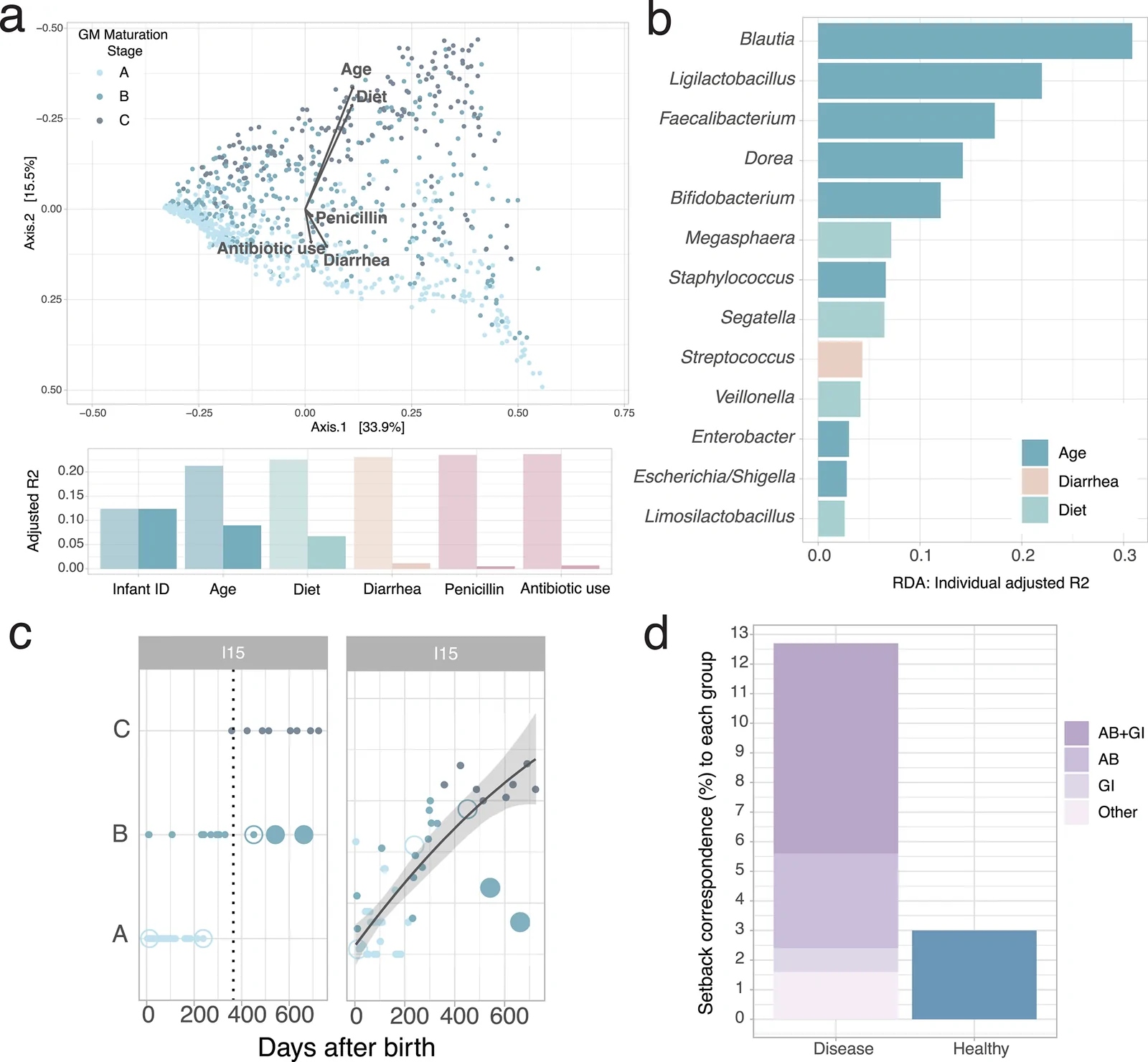
(**a**) Principal coordinate analysis (Bray-Curtis dissimilarity) showing the compositional variation between the three identified GMMs. Arrows indicate the effect size and direction of each significant covariate (univariate dbRDA, adjusted *P* < 0.05) regressed on the ordination coordinates. Bar plot: non-redundant covariates found to be significantly associated with the compositional variation on the bacterial genus level (multivariate dbRDA). Light-colored bars indicate the cumulative contribution of the covariates to the explained variance (*R*^2^) of the sample ordination. Darker-colored bars show the univariate explanatory power of covariates (univariate dbRDA *R*^2^). (**b**) Summary of the most influential covariates explaining the variation in the top 15 genera within the BBGUT cohort, using a multivariate distance-based redundancy analysis (dbRDA) on the relative abundances of each genus, controlling for infant identity, with FDR < 0.05. The bar lengths indicate the explanatory power of the most significant covariate, as determined by stepwise dbRDA (*R*²). (**c**) Example of the gut microbiota maturation stage succession over time for infant I15. Left: larger dots indicate time points where the microbiota reverted to a previous maturation stage. Right: changes in maturation scores over time for the same child. Maturation scores were calculated by averaging the ranks of the genera present in each sample (abundance threshold: 0.5%), based on their order of appearance over time. The black line represents a quadratic regression with a 95% confidence interval. Larger filled dots highlight time points where the maturation score dropped below this confidence interval, while empty large dots indicate a return to an earlier maturation stage without a corresponding score drop (left and right panels). Only setbacks accompanied by a maturation score decrease were considered as maturation setbacks. (**d**) Correspondence of samples with maturation setbacks across different health statuses. Samples are categorized as healthy or disease-associated**,** with further subcategories: AB + GI (samples collected during both antibiotic administration and gastrointestinal symptoms), AB (samples collected during antibiotic administration without gastrointestinal symptoms), GI (samples collected during gastrointestinal symptoms without antibiotic use), and Other (samples associated with other disease events, such as antifungal treatment or reported fungal infections at the time of sampling).

We analyzed a range of demographic factors (delivery mode/place, gestational age, hygiene levels, sibling presence), infant-specific variables (infant ID, age), and medical history (vaccination, gastrointestinal disease occurrence, antibiotic use), to identify the key covariates associated with compositional variation in the Bangladeshi cohort across all samples (*n* =984). Overall, the cumulative explanatory power of the non-redundant variables in our multivariate distance-based redundancy analysis (dbRDA) collectively explained approximately 24% of the variation in the microbial community composition (Fig. 3a; Table S6). Among significant covariates, infant identity, reflecting interindividual variation, had the highest explanatory power (*n* = 984, dbRDA with Bray-Curtis dissimilarity, *R*² =0.12, adjusted *P* < 0.01), followed by age (*R*² = 0.09, adjusted *P* < 0.01), diet (*R*² = 0.013, adjusted *P* < 0.01), diarrhea occurrence (*R*² = 0.006, adjusted *P* < 0.01), and antibiotic use (*R*² = 0.006, adjusted *P* < 0.05) (Fig. 3a; Table S6). Despite our findings being broadly consistent with previous observations in Belgian infants (10), medical history-related variables, such as diarrhea and antibiotic use, contributed more substantially to the explained variation in BBGUT, likely reflecting higher disease and medication exposure compared to BABEL. Whereas the tested covariates explained nearly 40% of variance in Belgian infants (10), they accounted for only 24% in the Bangladeshi cohort.

Additionally, the contribution of different metadata covariates to the variation of the most abundant bacterial genera of the BBGUT cohort (*n* = 984, multivariate stepwise distance-based redundancy analysis using Euclidean distance on composition) was also evaluated. Here, we found age to be the variable with the highest explanatory power in over 50% of the genera (8 out of 15) (Fig. 3b; Table S7). For only four taxa, namely, *Megasphaera, Segatella*, *Veillonella,* and *Limosilactobacillus*, diet (i.e., introduction to solid food) explained most variation in their abundance patterns. Interestingly, *Streptococcus*, one of the common bacterial genera in healthy early-life samples, was associated with diarrhea episodes.

Several occasional regressions (i.e., setbacks) to earlier GMM stages were observed during the gut microbiota maturation of the BBGUT cohort despite the conserved process generally followed in healthy samples (Fig. 1e). To better understand these fluctuations, we assessed the influence of external factors on gut microbiota maturation by considering both the setback changes and the level of maturation at the time of the change. The latter was quantified by calculating a maturation score (see Supplemental methods) considering the presence or absence of key bacterial genera. Only samples that exhibited a setback to a lower maturation stage, along with a corresponding decrease in maturation score, were included in the analysis (*n* = 31, across all samples; Fig. S11). This approach allowed for a more precise evaluation of external factors influencing gut microbiota development (example of infant 15 in Fig. 3c), revealing 31 setback instances throughout our cohort. To further assess which factors could explain these disruptions, we evaluated the metadata of these 31 samples and explored their associated health status. Instances with an observed maturation setback comprised only 3.1% of the total healthy time points (15/486) and 12.6% of the total disease-associated events (16/127) (Fig. 3d; Table S8). A chi-squared test confirmed that the occurrence of maturation setbacks is significantly associated with health status [χ² (1) =17.045, *P* = 3.65e−05] and positively associated with disease (Table S8). This positive association between maturation setbacks and disease-associated samples suggests a link between disease occurrence and maturation disruption. Notably, we observed a maturation setback for around 24% of the total disease events for which both antibiotic administration and gastrointestinal signs were reported at the same time (9/37). However, a specific association with these types of events and maturation setbacks was not confirmed statistically. Samples associated with setback events also exhibited lower microbial alpha diversity compared to samples from healthy timepoints preceding the setbacks (Fig. S12).

### Changes in the development of a healthy gut microbiota coincide with the first COVID-19 lockdown period in Bangladesh

**Fig 4:**
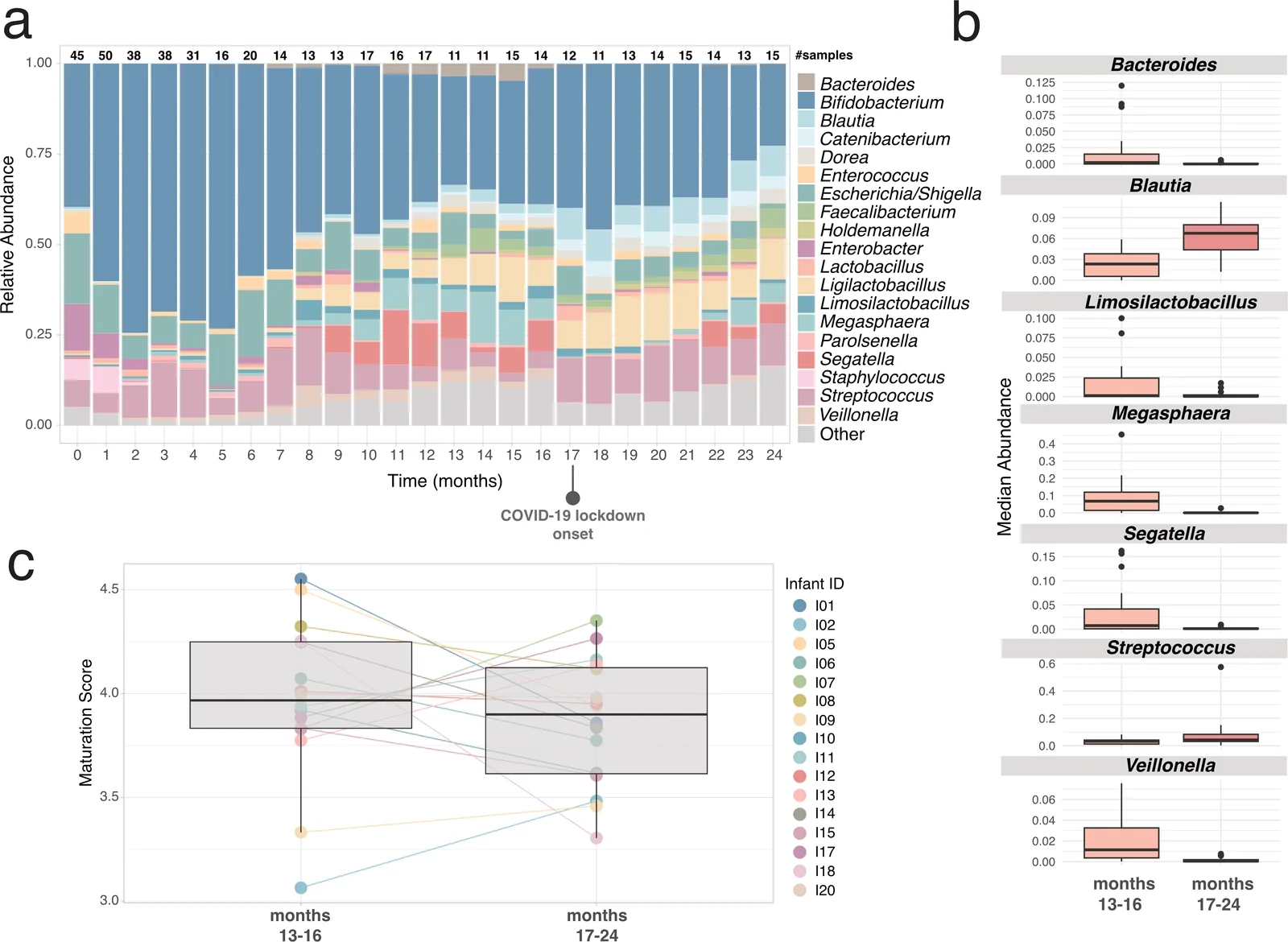
(**a**) Mean relative abundance of the most common bacterial genera found in the Bangladeshi infant cohort during the first 2 years of life (0–24 months). To account for the rapid changes in infant gut composition after birth, the first 10 days of life have been grouped as month 0. The number of healthy samples assigned to each month is shown at the top of each bar, and the period that coincides with onset of the first COVID-19 lockdown measures is marked on the *x*-axis. (**b**) Bacterial genera with a statistically significant difference in their median relative abundance when comparing the 13–16 month and 17–24 month groups during the second year of life (Wilcoxon signed-rank test, *P* < 0.05, FDR < 0.05). (**c**) Average weighted maturation score comparison between the two age periods during the second year of life. Each point represents an infant’s maturation score in each period calculated considering HTP samples only, with colors distinguishing different children. Lines connect scores for the same infant across the two periods, illustrating individual trajectories in maturation patterns. (Wilcoxon signed-rank test, *P* = 0.46). Note that data for the second year include 16 out of 20 infants.

While analyzing the monthly progression of gut microbiota maturation in the BBGUT cohort (considering only HTP samples), we noticed a qualitative decrease in the relative abundance of some taxa during the second year (Fig. 4a). To further assess these shifts, we compared period-specific changes in bacterial relative abundances and identified several genera with significantly greater fluctuations after 16 months of age (Fig. S13; Table S9). We, therefore, divided the second year into two periods, months 13–16 and months 17–24 of infant age. As all infants were born within a 36-day window, this boundary also coincides with the onset of the national COVID-19 lockdown in Bangladesh (26 March 2020; Fig. S14), meaning that infant age and calendar period cannot be distinguished in this cohort. For this analysis, we focused on samples representing healthy time points due to a limited number of samples associated with disease in year 2 (*n* = 17/175 samples). Comparing the two periods revealed a statistically significant decline in the relative abundance of several common bacteria, including *Segatella, Bacteroides, Veillonella,* and *Megasphaera*, observed during months 17–24 (Fig. 4b; Table S10). Only *Blautia*, a genus suggested to have probiotic potential (58) and *Streptococcus*, showed an increase in relative abundance during this time. Although changes in these bacterial taxa were observed, the current study design does not allow us to determine whether they were driven by pandemic-related factors.

Next, we compared maturation scores between infants before and after 16 months of age (calculated based on HTP samples), accounting for bacterial rankings and presence across monthly samples throughout their second year of life as previously discussed (Fig. 3b). Although there was no significant overall decrease in the maturation score during months 17–24 (Fig. 4c), and alpha diversity indices similarly showed no significant change between the two age periods (linear mixed model, *P* = 0.44 [Shannon index], *P* = 0.43 [observed richness]; Table S3), individual responses varied, with some infants showing signs of plateaued or reduced maturation. While these results do not support a consistent, cohort-wide delay in microbiota maturation (Fig. 4b), we identified temporal perturbations during this period, as reflected by changes in the relative abundance of specific bacterial taxa (Fig. 4c).

### The role of *Segatella* bacteria in the gut microbiota maturation of Bangladeshi infants remains unclear

Temporal changes in *Segatella* abundance in the gut microbiota of BBGUT infants were observed, with notable fluctuations in healthy samples (Fig. 4a). *Segatella* was first detected toward the end of the first year of life, but contrary to the stable prevalence typically seen in other Bangladeshi (24) and non-Western populations (17), its average abundance declined sharply between months 17 and 24 (Fig. 4a and b). This observation prompted us to further investigate the development of *Segatella*, as it was the dominant genus within the *Prevotellaceae* family present in this cohort and one of the most abundant bacterial genera in maternal samples (Fig. S7).

**Fig 5:**
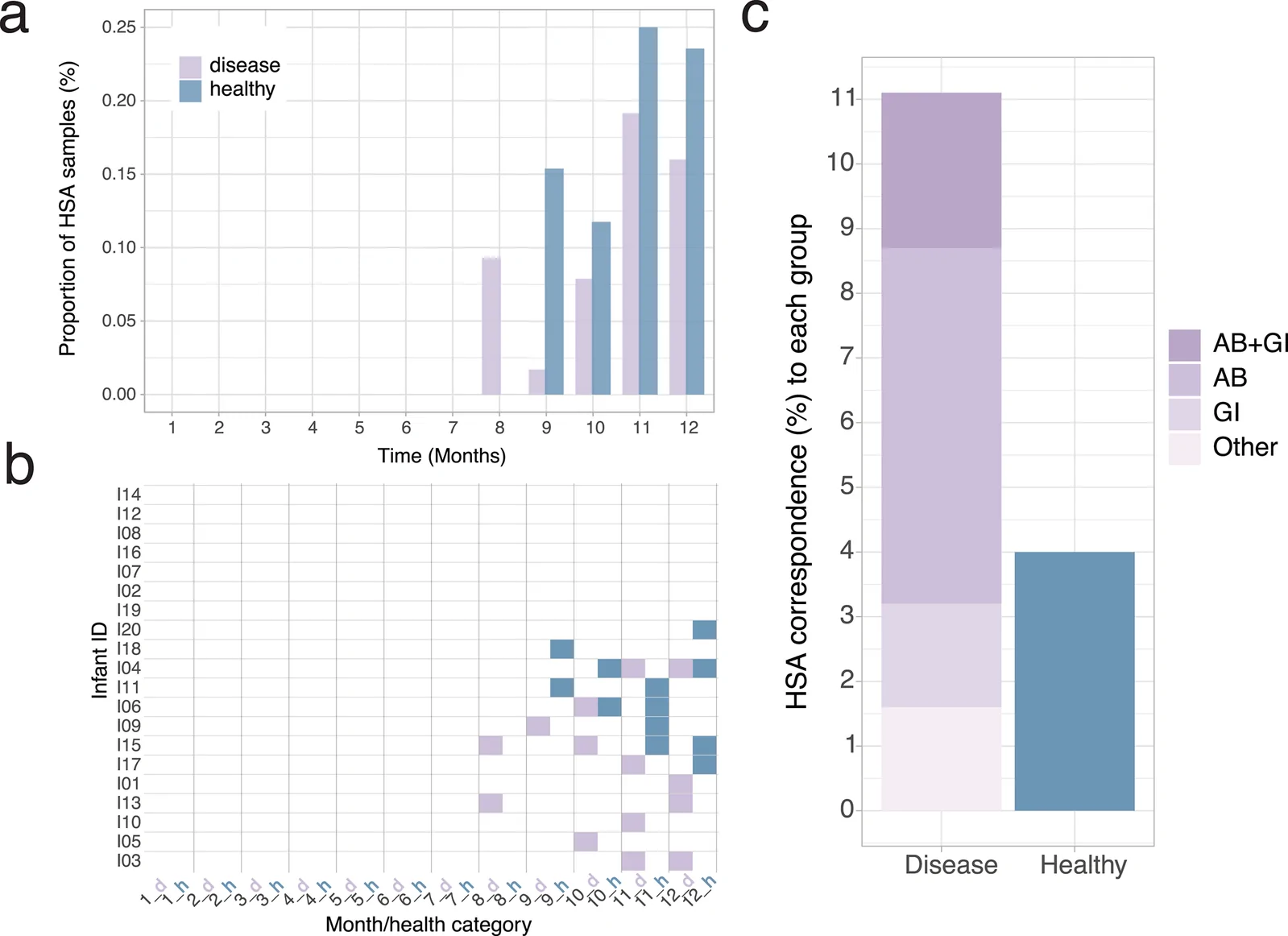
(**a**) Normalized proportion of samples with *Segatella* abundance greater than or equal to 0.25 (HSA samples, *n* = 33) relative to the number of healthy or diseased samples across each month in the first year of life. The bars represent the proportion of HSA samples within each health category, with colors indicating the health status (healthy, light purple; disease-related, blue). (**b**) Monthly presence of *Segatella* in infant samples across the two health categories (healthy and disease). To enable a direct comparison between disease and health-associated samples, the visualization is restricted to the first year of life, where both groups are adequately represented. The grid depicts the binary presence (1 = detected, 0 = not detected) of HSA samples for each child over a 12-month period. The *x*-axis represents the combination of month and health category, while the *y*-axis shows individual infant IDs. (**c**) Stacked bar plot illustrating the percentage of HSA corresponding to the different health categories (4% of all healthy samples and 11% of all disease-related events have ≥ 25% *Segatella* abundance). HSA samples related to antibiotic events (AB) represent 5.5% of the disease events, HSA samples associated with events with combined antibiotic administration and gastrointestinal disease occurrence (AB + GI) represent 2.4% of the disease events, and HSA samples linked to gastrointestinal disease events (GI) represent 1.6% of the disease episodes.

Further analysis revealed that the observed increase in *Segatella* prevalence was primarily driven by disease-associated samples in maturation stage C (Fisher’s exact test, *P* < 0.001; Fig. S15a and b). Thus, we focused on a subset of 33 samples exhibiting considerably high *Segatella* abundances (≥25%) during the first 2 years of life (Fig. 5a). We first examined the proportion of these high *Segatella* abundance (HSA) samples over time and found that they began to appear around month 8 (Fig. 5a). Although these samples were more prevalent in HTPs overall, *Segatella*-rich samples appeared earlier in samples associated with disease (Fig. 5a). In the first year of life, the first detection occurred during disease-related time points for 45% of infants (9 out of 20), compared to only 25% of infants (4 out of 20) whose first *Segatella*-rich samples appeared during HTPs (Fig. 5b).

When categorizing HSA samples by health status, they represented 4% of all healthy samples and 11% of all disease samples in the cohort (Fig. 5c; Table S11). Notably, within the disease-related group, most HSA samples were associated with antibiotic administration (Fig. 5c). A chi-squared test confirmed a significant association between the health categories and the occurrence of HSA samples [χ² (1) = 8.66, *P* = 0.003] (Table S11). Specifically, we observed a positive association between disease-related samples and elevated *Segatella* abundance, suggesting a link between *Segatella* spikes and disease occurrence.

Following this observation, we explored whether certain *Segatella* ASVs were enriched in disease-associated samples compared to those linked to healthy time points and found no significant differences (Fig. S16). To gain deeper insight into the taxonomical classification of *Segatella* sequences in this study, using the latest RDP taxonomy (training set 19), *Segatella* ASVs were identified as *Segatella copri* and *Segatella hominis* (Table S12). Within-individual comparison of HSA event samples against the closest preceding healthy time point (*n* = 33 paired events from 16 infants) identified *Bifidobacterium* as the only genus with a significant reduction in relative abundance during HSA events (Wilcoxon signed-rank test, adjusted *P* < 0.001; Fig. S17).

## DISCUSSION

The early development of gut microbiota is a dynamic process, with bacterial alpha diversity increasing over the first years of life (12, 13). In this study, we explored gut microbiota development in healthy Bangladeshi infants during the first 2 years of life. Western cohort studies have shown that gut microbiota development follows a successional and predictable trajectory of microbial acquisition (12, 41, 42). Consistent with these observations, we found that gut microbiota development in a non-Western infant cohort follows similar patterns to those reported in Western settings (10, 59), occurring in distinct maturation stages marked by an increase in bacterial richness and diversity.

The high abundance of *Bifidobacterium* observed here aligns with previous findings in Bangladeshi infant populations (21, 24, 47), supporting reports of more abundant bifidobacteria communities in non-Western infants (60, 61). Breastfeeding is more prevalent in non-Western communities (62) and breastfed infants harbor a more abundant community of lactic acid bacteria and *Bifidobacterium* (63, 64) which may also explain the high *Bacillota* (formerly *Firmicutes* [65]) abundance and diversity in our cohort.

Comparison of the healthy infant gut microbiota development in the BBGUT cohort with that of a Belgian (BABEL) cohort (10) during the first year of life revealed significant compositional differences where many bacterial genera exhibited differential prevalence and abundance. Examples of this are the exclusive presence of *Segatella* and the more diverse community of *Bacillota* in Bangladeshi infants contrasting with the higher abundances of *Escherichia/Shigella* taxa found in Belgian ones. Nevertheless, we also observe substantial similarities between the two populations in the overall acquisition patterns of the most abundant genera. In contrast to other studies reporting higher microbiota diversity in non-Western settings (16, 53, 56), our data showed that alpha diversity follows a comparable trajectory in the two cohorts during infancy. This aligns with findings by Yatsunenko and colleagues, which showed that differences in alpha diversity between U.S. and non-U.S. populations do not become apparent until after the third year of life (13). The order in which the early colonizing genera appeared occurred also in a similar manner, further supporting that bacterial colonization may follow similar succession patterns irrespective of geographical context or cultural heterogeneity as recently proposed (25). Our observations in a Bangladeshi infant population contradict the idea that breastfeeding cessation is the main driver of microbiota maturation—as seen in western infants (59, 63). Here, we observed gut microbiota maturation over time, reflected by increased alpha diversity and the emergence of more complex taxa, similar to that of the Belgian cohort despite extended breastfeeding. Another study conducted in a Bangladeshi infant cohort showed that the introduction of complementary foods was associated with shifts in microbiota composition and suggested that, in populations where breastfeeding continues alongside complementary feeding, dietary transitions may contribute to microbiota maturation beyond the effects of breastfeeding cessation alone (24). In addition to this, increased interpersonal transmission of gut microbes in LMIC communities may enhance microbial diversity (66), which could partially explain the observed bacterial diversity in BBGUT, regardless of breastfeeding status.

When looking at factors that influence gut microbiota development during early life, the combined variance explained by age, diet, disease, and antibiotic use accounted for less than 25% compared to almost 40% of variance explained by metadata variables in Belgian infants (10). This suggests that additional, unaccounted factors may play a significant role and need further investigation. For instance, Vatanen and colleagues showed that seasonal shifts (pre-monsoon and rainy monsoon seasons) affected the presence of several gut bacterial species in Bangladeshi infants (24). Stool consistency, which was not measured in this study, is also an important covariate explaining microbiota variation in healthy adult populations (27, 52) although this was not observed in Belgian infants (10). The role of host genetics in shaping gut microbiota composition is also widely recognized (67, 68). We propose that additional dimensions of cultural heterogeneity, potentially overlooked in this study, along with factors such as climate, seasonal variation, host genetics, and stool consistency, should be considered when investigating gut microbiota development, particularly in understudied populations.

Several studies have shown that neonatal antibiotic exposure can delay microbiota maturation, reduce bifidobacteria abundance, increase the abundance of *Enterobacteriaceae* species, and lower overall microbial diversity (10, 69–72). In LMICs, early-life exposure to a broad range of enteric pathogens, often subclinical infections, is common and may influence the typical colonization process (43). In this cohort, we report specific setbacks in microbiota maturation coinciding with antibiotic use and events of concurrent antibiotic administration and gastrointestinal disease. Despite these acute setbacks, no major persistent impacts on gut microbiota development were observed during early life. Maturation-stage regressions were also observed in healthy samples during the second year of life (Fig. 1e). In this study, this interval overlapped almost entirely with the lockdown period in Bangladesh, suggesting that these regressions cannot be attributed specifically to lockdown-related factors. Instead, the illness episodes and antibiotic exposures recorded during this period represent potential contributing factors more directly supported by the available data.

In addition, *Streptococcus*—one of the common bacterial genera in healthy early-life samples—was associated with diarrhea episodes. We hypothesize that this may be linked to potential enteric viral infections, which could increase the abundance of genera like *Streptococcus* (73, 74). However, as *Streptococcus* is a lactic acid bacterium, its colonization in the infant gut may also be promoted by sustained breastfeeding among other factors. Further research is required to elucidate the role and implications of *Streptococcus* presence in the healthy infant gut within this cohort.

Sampling in the BBGUT cohort partially overlapped with the early COVID-19 lockdown measures in Bangladesh, resulting in most of the second year being spent under restrictions. To our knowledge, this is the first longitudinal study of healthy infant gut microbiota development in Bangladesh to span the pandemic period within a single cohort. Although no cohort-wide changes in gut microbiota maturation were observed during the lockdown period (overlapping with months 17–24), taxon-specific changes were detected. During this period, we observed a reduction in relative abundance of genera associated with microbiota maturation, such as *Segatella* and *Megasphaera*. Although our results indicate a substantial presence of *Bacillota* in the first year of life, we do not observe the expected bacterial diversification and progressive increase in *Bacteroidota* abundances (formerly *Bacteroidetes* [65]) during the second year, usually observed in Bangladeshi (24) and other cohorts (12, 45, 59, 75). Several factors may account for these observations such as lockdown-driven lifestyle and dietary changes related to COVID-19. However, as these variables were not measured in our study, this interpretation remains speculative and the effect of such associations should be systematically assessed in future studies. For instance, extended breastfeeding throughout both years may have also contributed, especially if breastfeeding frequency was increased during the lockdown period, although this cannot be determined from our data. The lower intestinal pH in breastfed infants may inhibit the colonization of acid-sensitive bacteria, thereby shaping microbial composition, preventing pathogen growth, and potentially constraining overall microbial diversity (1, 76) even after solid food introduction. This effect may be more pronounced during periods of social distancing, limiting horizontal bacterial transmission. However, because all infants experienced the first COVID-19 lockdown at the same developmental stage, the effects of pandemic-related restrictions cannot be separated from normal age-related microbiota development in this study. Furthermore, we cannot exclude that changes in the sample handling upon collection during this period might have also affected the observed compositional profiles. Finally, the relatively small cohort size of this study might limit the generalizability of our findings. Nonetheless, to date, only limited studies have examined the impact of the COVID-19 pandemic on healthy infant gut microbiota development, reporting changes in both diversity and maturation (77–79). Further investigation into the long-term effects of the COVID-19 period is needed, particularly in LMICs, where the prevalence of other factors such as malnutrition could have compounding effects on the development of a healthy gut microbiome.

Finally, we observed that *Segatella* (formerly *Prevotella* [18]) was prevalent in disease-associated samples. High abundances of this genus occurred mostly during the first year of life in samples associated with disease episodes first appearing in the 8th month of life. Our findings indicate a positive association between high *Segatella* abundance and disease-related samples, most of which coincided with antibiotic exposure. In the developing infant gut, a *Bifidobacterium*-dominated community is progressively displaced by *Bacteroidetes*, including *Bacteroides* and *Prevotella/Segatella* clade members as complementary feeding is introduced and dietary complexity increases (24, 61). High *Segatella* abundance (HSA) events in our cohort superficially resemble this trajectory, with *Segatella* expanding while *Bifidobacterium* contracts; however, the extent to which this reflects a disease-associated perturbation remains unclear, and further work is required to resolve the underlying community dynamics. *Segatella* is known to be more prevalent in non-Western populations (15, 17, 56, 80), and its presence is often linked to a healthier diet with fewer processed foods compared to Western and more industrialized regions (81). Nonetheless, the role of *Segatella* within the gut microbiota remains controversial. *Segatella* has been reported as a marker of both health and disease, highlighting its complex and context-dependent nature (82). The high diversity observed among species within the genus, such as *Segatella copri*, contributes to the conflicting findings reported across studies (82–85). As our data revealed an association with disease in a non-Western infant cohort, future studies should prioritize higher taxonomic (i.e., species- and strain-level) resolution of *Segatella copri*, which may uncover functionally distinct variants that differ in their impacts on host health, as shown in other taxa (e.g., pathogenic and commensal *E. coli* strains [86–88]). This is critical to account for its documented phylogenetic and functional heterogeneity, as the species comprises multiple clades with distinct metabolic repertoires and divergent associations with host health, including links to inflammatory and autoimmune conditions such as Rheumatoid Arthritis, as well as to improved metabolic responses in the context of high-fiber diets (84, 89–91). Given its importance, variability, and debated role in gut health, detailed longitudinal and functional studies of *Segatella* are needed to elucidate its contributions to health and disease across diverse populations, particularly in early life, when gut microbiome development is highly dynamic and the presence and activity of *Segatella* may influence long-term health trajectories.

### Conclusion

This study provides a densely sampled longitudinal characterization of gut microbiota development in an LMIC setting, offering detailed insights into the maturation of the infant gut microbiome in Bangladesh. By comparing gut microbiota development to a Western infant cohort, we identified both conserved trajectories across geographies and cohort-specific compositional differences reflecting the influence of lifestyle and the environment. We are the first to characterize early-life gut microbiota development in a Bangladeshi infant population during COVID-19, providing insights into microbiota dynamics under these unique circumstances and highlighting the need for appropriate study designs and continued longitudinal monitoring to evaluate potential long-term impacts. In addition, we identified a link between *Segatella* and disease, adding to the growing and sometimes conflicting literature on its role in human health. Together, these findings offer new perspectives on gut microbiota development across diverse populations and emphasize the significance of environmental, cultural, and global disruption factors in shaping the infant gut microbiome. Looking ahead, extending these observations into later childhood and larger cohorts as well as integrating higher-resolution multi-omics approaches will be critical to deepen our understanding and to guide interventions that support infant health in LMICs.

## Supplementary Material

Reviewer comments

## Data Availability

The 16S rRNA sequencing data generated for the current work is available at the European Nucleotide Archive (ENA; https://www.ebi.ac.uk/ena, accession number PRJEB95966). All scripts for conducting the analysis and generating the figures are publicly available at https://github.com/Matthijnssenslab/Bangladesh_InfGut_16S.

## SUPPLEMENTAL MATERIAL

Supplemental Figures msystems.00101-26-s0001.docx Figures S1 to S17.

Supplemental Text msystems.00101-26-s0002.docx Detailed materials and methods.

Supplemental Tables msystems.00101-26-s0003.xlsx Tables S1 to S12.

OPEN PEER REVIEW reviewer-comments.pdf An accounting of the reviewer comments and feedback.

ASM does not own the copyrights to Supplemental Material that may be linked to, or accessed through, an article. The authors have granted ASM a non-exclusive, world-wide license to publish the Supplemental Material files. Please contact the corresponding author directly for reuse.

## References

[1] Milani C, Duranti S, Bottacini F, Casey E, Turroni F, Mahony J, Belzer C, Delgado Palacio S, Arboleya Montes S, Mancabelli L, Lugli GA, Rodriguez JM, Bode L, de Vos W, Gueimonde M, Margolles A, van Sinderen D, Ventura M. 2017. The first microbial colonizers of the human gut: composition, activities, and health implications of the infant gut microbiota. Microbiol Mol Biol Rev 81:e00036-17. doi:10.1128/MMBR.00036-17 PMC5706746 29118049

[2] Lim ES, Zhou Y, Zhao G, Bauer IK, Droit L, Ndao IM, Warner BB, Tarr PI, Wang D, Holtz LR. 2015. Early life dynamics of the human gut virome and bacterial microbiome in infants. Nat Med 21:1228–1234. doi:10.1038/nm.3950 PMC4710368 26366711

[3] Zheng D, Liwinski T, Elinav E. 2020. Interaction between microbiota and immunity in health and disease. Cell Res 30:492–506. doi:10.1038/s41422-020-0332-7 PMC7264227 32433595

[4] Arrieta M-C, Stiemsma LT, Dimitriu PA, Thorson L, Russell S, Yurist-Doutsch S, Kuzeljevic B, Gold MJ, Britton HM, Lefebvre DL, Subbarao P, Mandhane P, Becker A, McNagny KM, Sears MR, Kollmann T, Mohn WW, Turvey SE, Finlay BB, CHILD Study Investigators. 2015. Early infancy microbial and metabolic alterations affect risk of childhood asthma. Sci Transl Med 7:307ra152. doi:10.1126/scitranslmed.aab2271 26424567

[5] Kostic AD, Gevers D, Siljander H, Vatanen T, Hyötyläinen T, Hämäläinen A-M, Peet A, Tillmann V, Pöhö P, Mattila I, Lähdesmäki H, Franzosa EA, Vaarala O, de Goffau M, Harmsen H, Ilonen J, Virtanen SM, Clish CB, Orešič M, Huttenhower C, Knip M, Xavier RJ. 2015. The dynamics of the human infant gut microbiome in development and in progression toward type 1 diabetes. Cell Host Microbe 17:260–273. doi:10.1016/j.chom.2015.01.001 PMC4689191 25662751

[6] Depner M, Taft DH, Kirjavainen PV, Kalanetra KM, Karvonen AM, Peschel S, Schmausser-Hechfellner E, Roduit C, Frei R, Lauener R, Divaret-Chauveau A, Dalphin J-C, Riedler J, Roponen M, Kabesch M, Renz H, Pekkanen J, Farquharson FM, Louis P, Mills DA, von Mutius E, PASTURE study group, Ege MJ. 2020. Maturation of the gut microbiome during the first year of life contributes to the protective farm effect on childhood asthma. Nat Med 26:1766–1775. doi:10.1038/s41591-020-1095-x 33139948

[7] Zhang M, Sun K, Wu Y, Yang Y, Tso P, Wu Z. 2017. Interactions between intestinal microbiota and host immune response in inflammatory bowel disease. Front Immunol 8:942. doi:10.3389/fimmu.2017.00942 PMC5558048 28855901

[8] Hoskinson C, Dai DLY, Del Bel KL, Becker AB, Moraes TJ, Mandhane PJ, Finlay BB, Simons E, Kozyrskyj AL, Azad MB, Subbarao P, Petersen C, Turvey SE. 2023. Delayed gut microbiota maturation in the first year of life is a hallmark of pediatric allergic disease. Nat Commun 14:4785. doi:10.1038/s41467-023-40336-4 PMC10465508 37644001

[9] Derrien M, Alvarez A-S, de Vos WM. 2019. The gut microbiota in the first decade of life. Trends Microbiol 27:997–1010. doi:10.1016/j.tim.2019.08.001 31474424

[10] Beller L, Deboutte W, Falony G, Vieira-Silva S, Tito RY, Valles-Colomer M, Rymenans L, Jansen D, Van Espen L, Papadaki MI, Shi C, Yinda CK, Zeller M, Faust K, Van Ranst M, Raes J, Matthijnssens J. 2021. Successional stages in infant gut microbiota maturation. mBio 12:e01857-21. doi:10.1128/mBio.01857-21 PMC8686833 34903050

[11] Xiao L, Zhao F. 2023. Microbial transmission, colonisation and succession: from pregnancy to infancy. Gut 72:772–786. doi:10.1136/gutjnl-2022-328970 PMC10086306 36720630

[12] Nunez H, Nieto PA, Mars RA, Ghavami M, Sew Hoy C, Sukhum K. 2025. Early life gut microbiome and its impact on childhood health and chronic conditions. Gut Microbes 17:2463567. doi:10.1080/19490976.2025.2463567 PMC11810090 39916516

[13] Yatsunenko T, Rey FE, Manary MJ, Trehan I, Dominguez-Bello MG, Contreras M, Magris M, Hidalgo G, Baldassano RN, Anokhin AP, Heath AC, Warner B, Reeder J, Kuczynski J, Caporaso JG, Lozupone CA, Lauber C, Clemente JC, Knights D, Knight R, Gordon JI. 2012. Human gut microbiome viewed across age and geography. Nature 486:222–227. doi:10.1038/nature11053 PMC3376388 22699611

[14] Bolte EE, Moorshead D, Aagaard KM. 2022. Maternal and early life exposures and their potential to influence development of the microbiome. Genome Med 14:4. doi:10.1186/s13073-021-01005-7 PMC8751292 35016706

[15] Obregon-Tito AJ, Tito RY, Metcalf J, Sankaranarayanan K, Clemente JC, Ursell LK, Zech Xu Z, Van Treuren W, Knight R, Gaffney PM, Spicer P, Lawson P, Marin-Reyes L, Trujillo-Villarroel O, Foster M, Guija-Poma E, Troncoso-Corzo L, Warinner C, Ozga AT, Lewis CM. 2015. Subsistence strategies in traditional societies distinguish gut microbiomes. Nat Commun 6:6505. doi:10.1038/ncomms7505 PMC4386023 25807110

[16] Lin A, Bik EM, Costello EK, Dethlefsen L, Haque R, Relman DA, Singh U. 2013. Distinct distal gut microbiome diversity and composition in healthy children from Bangladesh and the United States. PLoS One 8:e53838. doi:10.1371/journal.pone.0053838 PMC3551965 23349750

[17] de Goffau MC, Jallow AT, Sanyang C, Prentice AM, Meagher N, Price DJ, Revill PA, Parkhill J, Pereira DIA, Wagner J. 2022. Gut microbiomes from Gambian infants reveal the development of a non-industrialized *Prevotella-*based trophic network. Nat Microbiol 7:132–144. doi:10.1038/s41564-021-01023-6 PMC8727306 34972822

[18] Hitch TCA, Bisdorf K, Afrizal A, Riedel T, Overmann J, Strowig T, Clavel T. 2022. A taxonomic note on the genus *Prevotella*: description of four novel genera and emended description of the genera *Hallella* and *Xylanibacter**.* Syst Appl Microbiol 45:126354. doi:10.1016/j.syapm.2022.126354 36067550

[19] Arumugam M, Raes J, Pelletier E, Le Paslier D, Yamada T, Mende DR, Fernandes GR, Tap J, Bruls T, Batto J-M, et al.. 2011. Enterotypes of the human gut microbiome. Nature 473:174–180. doi:10.1038/nature09944 PMC3728647 21508958

[20] Abdill RJ, Adamowicz EM, Blekhman R. 2022. Public human microbiome data are dominated by highly developed countries. PLoS Biol 20:e3001536. doi:10.1371/journal.pbio.3001536 PMC8846514 35167588

[21] Subramanian S, Huq S, Yatsunenko T, Haque R, Mahfuz M, Alam MA, Benezra A, DeStefano J, Meier MF, Muegge BD, Barratt MJ, VanArendonk LG, Zhang Q, Province MA, Petri WA Jr, Ahmed T, Gordon JI. 2014. Persistent gut microbiota immaturity in malnourished Bangladeshi children. Nature 510:417–421. doi:10.1038/nature13421 PMC4189846 24896187

[22] Robertson RC, Edens TJ, Carr L, Mutasa K, Gough EK, Evans C, Geum HM, Baharmand I, Gill SK, Ntozini R, Smith LE, Chasekwa B, Majo FD, Tavengwa NV, Mutasa B, Francis F, Tome J, Stoltzfus RJ, Humphrey JH, Prendergast AJ, Manges AR. 2023. The gut microbiome and early-life growth in a population with high prevalence of stunting. Nat Commun 14:654. doi:10.1038/s41467-023-36135-6 PMC9929340 36788215

[23] Maghini DG, Oduaran OH, Olubayo LAI, Cook JA, Smyth N, Mathema T, Belger CW, Agongo G, Boua PR, Choma SSR, et al.. 2025. Expanding the human gut microbiome atlas of Africa. Nature 638:718–728. doi:10.1038/s41586-024-08485-8 PMC11839480 39880958

[24] Vatanen T, Ang QY, Siegwald L, Sarker SA, Le Roy CI, Duboux S, Delannoy-Bruno O, Ngom-Bru C, Boulangé CL, Stražar M, et al.. 2022. A distinct clade of *Bifidobacterium longum* in the gut of Bangladeshi children thrives during weaning. Cell 185:4280–4297. doi:10.1016/j.cell.2022.10.011 36323316

[25] Fahur Bottino G, Bonham KS, Patel F, McCann S, Zieff M, Naspolini N, Ho D, Portlock T, Joos R, Midani FS, Schüroff P, Das A, Shennon I, Wilson BC, O’Sullivan JM, Britton RA, Murray DM, Kiely ME, Taddei CR, Beltrão-Braga PCB, Campos AC, Polanczyk GV, Huttenhower C, Donald KA, Klepac-Ceraj V. 2025. Early life microbial succession in the gut follows common patterns in humans across the globe. Nat Commun 16:660. doi:10.1038/s41467-025-56072-w PMC11733223 39809768

[26] Gordon JI, Barratt MJ, Hibberd MC, Rahman M, Ahmed T. 2024. Establishing human microbial observatory programs in low- and middle-income countries. Ann NY Acad Sci 1540:13–20. doi:10.1111/nyas.15224 39298326

[27] Falony G, Joossens M, Vieira-Silva S, Wang J, Darzi Y, Faust K, Kurilshikov A, Bonder MJ, Valles-Colomer M, Vandeputte D, et al.. 2016. Population-level analysis of gut microbiome variation. Science 352:560–564. doi:10.1126/science.aad3503 27126039

[28] Tito RY, Cypers H, Joossens M, Varkas G, Van Praet L, Glorieus E, Van den Bosch F, De Vos M, Raes J, Elewaut D. 2017. Brief report: dialister as a microbial marker of disease activity in *Spondyloarthritis**.* Arthritis Rheumatol 69:114–121. doi:10.1002/art.39802 27390077

[29] Hildebrand F, Tadeo R, Voigt AY, Bork P, Raes J. 2014. LotuS: an efficient and user-friendly OTU processing pipeline. Microbiome 2:30. doi:10.1186/2049-2618-2-30 PMC4179863 27367037

[30] Callahan BJ, McMurdie PJ, Rosen MJ, Han AW, Johnson AJA, Holmes SP. 2016. DADA2: High-resolution sample inference from Illumina amplicon data. Nat Methods 13:581–583. doi:10.1038/nmeth.3869 PMC4927377 27214047

[31] Wang Q, Cole JR. 2024. Updated RDP taxonomy and RDP Classifier for more accurate taxonomic classification. Microbiol Resour Announc 13:e01063-23. doi:10.1128/mra.01063-23 PMC11008197 38436268

[32] Davis NM, Proctor DM, Holmes SP, Relman DA, Callahan BJ. 2018. Simple statistical identification and removal of contaminant sequences in marker-gene and metagenomics data. Microbiome 6:226. doi:10.1186/s40168-018-0605-2 PMC6298009 30558668

[33] Wickham H. 2016. Ggplot2: elegant graphics for data analysis. Springer-Verlag New York.

[34] Oksanen J, et al.. 2025. Vegan: Community Ecology Package. R package version

[35] McMurdie PJ, Holmes S. 2013. Phyloseq: an R package for reproducible interactive analysis and graphics of microbiome census data. PLoS one 8:e61217. doi:10.1371/journal.pone.0061217 PMC3632530 23630581

[36] Lahti L, et al.. 2017. Tools for microbiome analysis in R. Microbiome package version 1.23.1

[37] Barnett DJM, Arts ICW, Penders J. 2021. microViz: an R package for microbiome data visualization and statistics. JOSS 6:3201. doi:10.21105/joss.03201

[38] Bates D, Mächler M, Bolker B, Walker S. 2015. Fitting linear mixed-effects models using lme4. J Stat Softw 67:1–48. doi:10.18637/jss.v067.i01

[39] Lenth RV. 2016. Least-squares means: the R package lsmeans. J Stat Softw 69:1–33. doi:10.18637/jss.v069.i01

[40] Espey MG. 2013. Role of oxygen gradients in shaping redox relationships between the human intestine and its microbiota. Free Radic Biol Med 55:130–140. doi:10.1016/j.freeradbiomed.2012.10.554 23127782

[41] Tanaka M, Nakayama J. 2017. Development of the gut microbiota in infancy and its impact on health in later life. Allergol Int 66:515–522. doi:10.1016/j.alit.2017.07.010 28826938

[42] Arrieta M-C, Stiemsma LT, Amenyogbe N, Brown EM, Finlay B. 2014. The intestinal microbiome in early life: health and disease. Front Immunol 5:427. doi:10.3389/fimmu.2014.00427 PMC4155789 25250028

[43] Robertson RC, Manges AR, Finlay BB, Prendergast AJ. 2019. The human microbiome and child growth - first 1000 days and beyond. Trends Microbiol 27:131–147. doi:10.1016/j.tim.2018.09.008 30529020

[44] Zhang C, Li L, Jin B, Xu X, Zuo X, Li Y, Li Z. 2021. The effects of delivery mode on the gut microbiota and health: state of art. Front Microbiol 12:724449. doi:10.3389/fmicb.2021.724449 PMC8733716 35002992

[45] Roswall J, Olsson LM, Kovatcheva-Datchary P, Nilsson S, Tremaroli V, Simon M-C, Kiilerich P, Akrami R, Krämer M, Uhlén M, Gummesson A, Kristiansen K, Dahlgren J, Bäckhed F. 2021. Developmental trajectory of the healthy human gut microbiota during the first 5 years of life. Cell Host Microbe 29:765–776. doi:10.1016/j.chom.2021.02.021 33794185

[46] Taft DH, Lewis ZT, Nguyen N, Ho S, Masarweh C, Dunne-Castagna V, Tancredi DJ, Huda MN, Stephensen CB, Hinde K, von Mutius E, Kirjavainen PV, Dalphin J-C, Lauener R, Riedler J, Smilowitz JT, German JB, Morrow AL, Mills DA. 2022. *Bifidobacterium* species colonization in infancy: a global cross-sectional comparison by population history of breastfeeding. Nutrients 14:1423. doi:10.3390/nu14071423 PMC9003546 35406036

[47] Huda MN, Lewis Z, Kalanetra KM, Rashid M, Ahmad SM, Raqib R, Qadri F, Underwood MA, Mills DA, Stephensen CB. 2014. Stool microbiota and vaccine responses of infants. Pediatrics 134:e362–72. doi:10.1542/peds.2013-3937 PMC4187229 25002669

[48] Gehrig JL, Venkatesh S, Chang H-W, Hibberd MC, Kung VL, Cheng J, Chen RY, Subramanian S, Cowardin CA, Meier MF, et al.. 2019. Effects of microbiota-directed foods in gnotobiotic animals and undernourished children. Science 365:eaau4732. doi:10.1126/science.aau4732 PMC6683325 31296738

[49] Homann C-M, Rossel CAJ, Dizzell S, Bervoets L, Simioni J, Li J, Gunn E, Surette MG, de Souza RJ, Mommers M, Hutton EK, Morrison KM, Penders J, van Best N, Stearns JC. 2021. Infants’ first solid foods: impact on gut microbiota development in two intercontinental cohorts. Nutrients 13:2639. doi:10.3390/nu13082639 PMC8400337 34444798

[50] Xiao L, Wang J, Zheng J, Li X, Zhao F. 2021. Deterministic transition of enterotypes shapes the infant gut microbiome at an early age. Genome Biol 22:243. doi:10.1186/s13059-021-02463-3 PMC8383385 34429130

[51] Lewis SJ, Heaton KW. 1997. Stool form scale as a useful guide to intestinal transit time. Scand J Gastroenterol 32:920–924. doi:10.3109/00365529709011203 9299672

[52] Vandeputte D, Falony G, Vieira-Silva S, Tito RY, Joossens M, Raes J. 2016. Stool consistency is strongly associated with gut microbiota richness and composition, enterotypes and bacterial growth rates. Gut 65:57–62. doi:10.1136/gutjnl-2015-309618 PMC4717365 26069274

[53] De Filippo C, Cavalieri D, Di Paola M, Ramazzotti M, Poullet JB, Massart S, Collini S, Pieraccini G, Lionetti P. 2010. Impact of diet in shaping gut microbiota revealed by a comparative study in children from Europe and rural Africa. Proc Natl Acad Sci USA 107:14691–14696. doi:10.1073/pnas.1005963107 PMC2930426 20679230

[54] Vieira-Silva S, Falony G, Darzi Y, Lima-Mendez G, Garcia Yunta R, Okuda S, Vandeputte D, Valles-Colomer M, Hildebrand F, Chaffron S, Raes J. 2016. Species-function relationships shape ecological properties of the human gut microbiome. Nat Microbiol 1:16088. doi:10.1038/nmicrobiol.2016.88 27573110

[55] Mudgil D, Barak S. 2013. Composition, properties and health benefits of indigestible carbohydrate polymers as dietary fiber: a review. Int J Biol Macromol 61:1–6. doi:10.1016/j.ijbiomac.2013.06.044 23831534

[56] Schnorr SL, Candela M, Rampelli S, Centanni M, Consolandi C, Basaglia G, Turroni S, Biagi E, Peano C, Severgnini M, Fiori J, Gotti R, De Bellis G, Luiselli D, Brigidi P, Mabulla A, Marlowe F, Henry AG, Crittenden AN. 2014. Gut microbiome of the Hadza hunter-gatherers. Nat Commun 5:3654. doi:10.1038/ncomms4654 PMC3996546 24736369

[57] Kuang Y-S, Li S-H, Guo Y, Lu J-H, He J-R, Luo B-J, Jiang F-J, Shen H, Papasian CJ, Pang H, Xia H-M, Deng H-W, Qiu X. 2016. Composition of gut microbiota in infants in China and global comparison. Sci Rep 6:36666. doi:10.1038/srep36666 PMC5101483 27827448

[58] Liu X, Mao B, Gu J, Wu J, Cui S, Wang G, Zhao J, Zhang H, Chen W. 2021. *Blautia*-a new functional genus with potential probiotic properties? Gut Microbes 13:1–21. doi:10.1080/19490976.2021.1875796 PMC7872077 33525961

[59] Stewart CJ, Ajami NJ, O’Brien JL, Hutchinson DS, Smith DP, Wong MC, Ross MC, Lloyd RE, Doddapaneni H, Metcalf GA, et al.. 2018. Temporal development of the gut microbiome in early childhood from the TEDDY study. Nature 562:583–588. doi:10.1038/s41586-018-0617-x PMC6415775 30356187

[60] Grześkowiak Ł, Collado MC, Mangani C, Maleta K, Laitinen K, Ashorn P, Isolauri E, Salminen S. 2012. Distinct gut microbiota in southeastern African and northern European infants. J Pediatr Gastroenterol Nutr 54:812–816. doi:10.1097/MPG.0b013e318249039c 22228076

[61] Olm MR, Dahan D, Carter MM, Merrill BD, Yu FB, Jain S, Meng X, Tripathi S, Wastyk H, Neff N, Holmes S, Sonnenburg ED, Jha AR, Sonnenburg JL. 2022. Robust variation in infant gut microbiome assembly across a spectrum of lifestyles. Science 376:1220–1223. doi:10.1126/science.abj2972 PMC9894631 35679413

[62] Victora CG, Bahl R, Barros AJD, França GVA, Horton S, Krasevec J, Murch S, Sankar MJ, Walker N, Rollins NC, Lancet Breastfeeding Series Group. 2016. Breastfeeding in the 21st century: epidemiology, mechanisms, and lifelong effect. Lancet 387:475–490. doi:10.1016/S0140-6736(15)01024-7 26869575

[63] Bäckhed F, Roswall J, Peng Y, Feng Q, Jia H, Kovatcheva-Datchary P, Li Y, Xia Y, Xie H, Zhong H, et al.. 2015. Dynamics and stabilization of the human gut microbiome during the first year of life. Cell Host Microbe 17:690–703. doi:10.1016/j.chom.2015.04.004 25974306

[64] Solís G, de los Reyes-Gavilan CG, Fernández N, Margolles A, Gueimonde M. 2010. Establishment and development of lactic acid bacteria and bifidobacteria microbiota in breast-milk and the infant gut. Anaerobe 16:307–310. doi:10.1016/j.anaerobe.2010.02.004 20176122

[65] Oren A, Garrity GM. 2021. Valid publication of the names of forty-two phyla of prokaryotes. Int J Syst Evol Microbiol 71. doi:10.1099/ijsem.0.005056 34694987

[66] Zuo T, Kamm MA, Colombel J-F, Ng SC. 2018. Urbanization and the gut microbiota in health and inflammatory bowel disease. Nat Rev Gastroenterol Hepatol 15:440–452. doi:10.1038/s41575-018-0003-z 29670252

[67] Lopera-Maya EA, Kurilshikov A, van der Graaf A, Hu S, Andreu-Sánchez S, Chen L, Vila AV, Gacesa R, Sinha T, Collij V, et al.. 2022. Effect of host genetics on the gut microbiome in 7,738 participants of the Dutch Microbiome Project. Nat Genet 54:143–151. doi:10.1038/s41588-021-00992-y 35115690

[68] Kurilshikov A, Medina-Gomez C, Bacigalupe R, Radjabzadeh D, Wang J, Demirkan A, Le Roy CI, Raygoza Garay JA, Finnicum CT, Liu X, et al.. 2021. Large-scale association analyses identify host factors influencing human gut microbiome composition. Nat Genet 53:156–165. doi:10.1038/s41588-020-00763-1 PMC8515199 33462485

[69] Vatanen T, Franzosa EA, Schwager R, Tripathi S, Arthur TD, Vehik K, Lernmark Å, Hagopian WA, Rewers MJ, She J-X, Toppari J, Ziegler A-G, Akolkar B, Krischer JP, Stewart CJ, Ajami NJ, Petrosino JF, Gevers D, Lähdesmäki H, Vlamakis H, Huttenhower C, Xavier RJ. 2018. The human gut microbiome in early-onset type 1 diabetes from the TEDDY study. Nature 562:589–594. doi:10.1038/s41586-018-0620-2 PMC6296767 30356183

[70] Korpela K, Salonen A, Saxen H, Nikkonen A, Peltola V, Jaakkola T, de Vos W, Kolho K-L. 2020. Antibiotics in early life associate with specific gut microbiota signatures in a prospective longitudinal infant cohort. Pediatr Res 88:438–443. doi:10.1038/s41390-020-0761-5 31954376

[71] Luchen CC, Chibuye M, Spijker R, Simuyandi M, Chisenga C, Bosomprah S, Chilengi R, Schultsz C, Mende DR, Harris VC. 2023. Impact of antibiotics on gut microbiome composition and resistome in the first years of life in low- to middle-income countries: a systematic review. PLoS Med 20:e1004235. doi:10.1371/journal.pmed.1004235 PMC10298773 37368871

[72] Baldi A, Braat S, Imrul Hasan M, Bennett C, Barrios M, Jones N, Moir-Meyer G, Abdul Azeez I, Wilcox S, Saiful Alam Bhuiyan M, Ataide R, Clucas D, Harrison LC, Arifeen SE, Bowden R, Biggs B-A, Jex A, Pasricha S-R. 2024. Community use of oral antibiotics transiently reprofiles the intestinal microbiome in young Bangladeshi children. Nat Commun 15:6980. doi:10.1038/s41467-024-51326-5 PMC11324872 39143045

[73] Xiong L, Li Y, Li J, Yang J, Shang L, He X, Liu L, Luo Y, Xie X. 2021. Intestinal microbiota profiles in infants with acute gastroenteritis caused by rotavirus and norovirus infection: a prospective cohort study. Int J Infect Dis 111:76–84. doi:10.1016/j.ijid.2021.08.024 34411719

[74] Tesfaw G, Siraj DS, Abdissa A, Jakobsen RR, Johansen ØH, Zangenberg M, Hanevik K, Mekonnen Z, Langeland N, Bjørang O, Safdar N, Mapes AC, Kates A, Krych L, Castro-Mejía JL, Nielsen DS. 2024. Gut microbiota patterns associated with duration of diarrhea in children under five years of age in Ethiopia. Nat Commun 15:7532. doi:10.1038/s41467-024-51464-w PMC11369280 39223134

[75] Wernroth M-L, Peura S, Hedman AM, Hetty S, Vicenzi S, Kennedy B, Fall K, Svennblad B, Andolf E, Pershagen G, Theorell-Haglöw J, Nguyen D, Sayols-Baixeras S, Dekkers KF, Bertilsson S, Almqvist C, Dicksved J, Fall T. 2022. Development of gut microbiota during the first 2 years of life. Sci Rep 12:9080. doi:10.1038/s41598-022-13009-3 PMC9156670 35641542

[76] Brinck JE, Sinha AK, Laursen MF, Dragsted LO, Raes J, Uribe RV, Walter J, Roager HM, Licht TR. 2025. Intestinal pH: a major driver of human gut microbiota composition and metabolism. Nat Rev Gastroenterol Hepatol 22:639–656. doi:10.1038/s41575-025-01092-6 40603778

[77] Zhang L, Xu W, Meng HYH, Ching JYL, Liu Y, Wang S, Yan S, Lin L, Cheong PK, Ip KL, Peng Y, Zhu J, Cheung CP, Leung TF, Leung ASY, Tam WH, Leung TY, Chan PKS, Chang EB, Rubin DT, Claud EC, Wu WKK, Tun HM, Chan FKL, Ng SC. 2025. Impacts of COVID-19 pandemic on early life gut microbiome. Gut Microbes 17. doi:10.1080/19490976.2024.2443117 PMC12931694 41411654

[78] Querdasi FR, Vogel SC, Thomason ME, Callaghan BL, Brito NH. 2023. A comparison of the infant gut microbiome before versus after the start of the covid-19 pandemic. Sci Rep 13:13289. doi:10.1038/s41598-023-40102-y PMC10432475 37587195

[79] Wang J, Li Y, Mu Y, Huang K, Li D, Lan C, Cui Y, Wang J. 2024. Missing microbes in infants and children in the COVID-19 pandemic: a study of 1,126 participants in Beijing, China. Sci China Life Sci 67:1739–1750. doi:10.1007/s11427-023-2488-0 38748355

[80] Pasolli E, Asnicar F, Manara S, Zolfo M, Karcher N, Armanini F, Beghini F, Manghi P, Tett A, Ghensi P, Collado MC, Rice BL, DuLong C, Morgan XC, Golden CD, Quince C, Huttenhower C, Segata N. 2019. Extensive unexplored human microbiome diversity revealed by over 150,000 genomes from metagenomes spanning age, geography, and lifestyle. Cell 176:649–662. doi:10.1016/j.cell.2019.01.001 PMC6349461 30661755

[81] Smits SA, Leach J, Sonnenburg ED, Gonzalez CG, Lichtman JS, Reid G, Knight R, Manjurano A, Changalucha J, Elias JE, Dominguez-Bello MG, Sonnenburg JL. 2017. Seasonal cycling in the gut microbiome of the Hadza hunter-gatherers of Tanzania. Science 357:802–806. doi:10.1126/science.aan4834 PMC5891123 28839072

[82] Abdelsalam NA, Hegazy SM, Aziz RK. 2023. The curious case of *Prevotella copri*. Gut Microbes 15:2249152. doi:10.1080/19490976.2023.2249152 PMC10478744 37655441

[83] Blanco-Míguez A, Gálvez EJC, Pasolli E, De Filippis F, Amend L, Huang KD, Manghi P, Lesker T-R, Riedel T, Cova L, Punčochář M, Thomas AM, Valles-Colomer M, Schober I, Hitch TCA, Clavel T, Berry SE, Davies R, Wolf J, Spector TD, Overmann J, Tett A, Ercolini D, Segata N, Strowig T. 2023. Extension of the *Segatella copri* complex to 13 species with distinct large extrachromosomal elements and associations with host conditions. Cell Host Microbe 31:1804–1819. doi:10.1016/j.chom.2023.09.013 PMC10635906 37883976

[84] Tett A, Huang KD, Asnicar F, Fehlner-Peach H, Pasolli E, Karcher N, Armanini F, Manghi P, Bonham K, Zolfo M, et al.. 2019. The *Prevotella copri* complex comprises four distinct clades underrepresented in westernized populations. Cell Host Microbe 26:666–679. doi:10.1016/j.chom.2019.08.018 PMC6854460 31607556

[85] Xiao X, Singh A, Giometto A, Brito IL. 2024. Segatella clades adopt distinct roles within a single individual’s gut. NPJ Biofilms Microbiomes 10:114. doi:10.1038/s41522-024-00590-w PMC11514259 39465298

[86] Fang X, Monk JM, Nurk S, Akseshina M, Zhu Q, Gemmell C, Gianetto-Hill C, Leung N, Szubin R, Sanders J, Beck PL, Li W, Sandborn WJ, Gray-Owen SD, Knight R, Allen-Vercoe E, Palsson BO, Smarr L. 2018. Metagenomics-based, strain-level analysis of *Escherichia coli* from a time-series of microbiome samples from a crohn’s disease patient. Front Microbiol 9:2559. doi:10.3389/fmicb.2018.02559 PMC6218438 30425690

[87] Hu D, Fuller NR, Caterson ID, Holmes AJ, Reeves PR. 2022. Single-gene long-read sequencing illuminates *Escherichia coli* strain dynamics in the human intestinal microbiome. Cell Rep 38:110239. doi:10.1016/j.celrep.2021.110239 35021078

[88] Hazen TH, Michalski JM, Tennant SM, Rasko DA. 2023. Genomic diversity of non-diarrheagenic fecal *Escherichia coli* from children in sub-Saharan Africa and south Asia and their relatedness to diarrheagenic *E. coli*. Nat Commun 14:1400. doi:10.1038/s41467-023-36337-y PMC10011798 36918537

[89] De Filippis F, Pasolli E, Tett A, Tarallo S, Naccarati A, De Angelis M, Neviani E, Cocolin L, Gobbetti M, Segata N, Ercolini D. 2019. Distinct genetic and functional traits of human intestinal *Prevotella copri* strains are associated with different habitual diets. Cell Host Microbe 25:444–453. doi:10.1016/j.chom.2019.01.004 30799264

[90] Scher JU, Sczesnak A, Longman RS, Segata N, Ubeda C, Bielski C, Rostron T, Cerundolo V, Pamer EG, Abramson SB, Huttenhower C, Littman DR. 2013. Expansion of intestinal *Prevotella copri* correlates with enhanced susceptibility to arthritis. Elife 2:e01202. doi:10.7554/eLife.01202 PMC3816614 24192039

[91] Pedersen HK, Gudmundsdottir V, Nielsen HB, Hyotylainen T, Nielsen T, Jensen BAH, Forslund K, Hildebrand F, Prifti E, Falony G, et al.. 2016. Human gut microbes impact host serum metabolome and insulin sensitivity. Nature 535:376–381. doi:10.1038/nature18646 27409811

