# Supplementary material for: Global and local trajectories of infant gut microbiota development in Bangladesh across the COVID-19 pandemic
Source: mSystems. 2026 Sep 1;11(9):e00101-26. doi: 10.1128/msystems.00101-26 (PMC13595919; doi:10.1128/msystems.00101-26)
Supplement: Supplementary file 1 [file reviewer-comments.pdf]

ASM | mSystems

Global and local trajectories of infant gut microbiota development in Bangladesh across the COVID-19 pandemic

Maria Ioanna Papadaki, Gwen Falony, Sara Vieira-Silva, Raul Tito, Leen Rymenans, Jill Swinnen, Lila Close, Jara Wagemans, Shafina Jahan, Muntasir Alam, Kamrun Nahar, Mustafizur Rahman, Jeroen Raes, and Jelle Matthijnssens

Corresponding Author(s): Maria Ioanna Papadaki, Katholieke Universiteit Leuven

Review Timeline:

Submission Date:	January 30, 2026
Editorial Decision:	April 7, 2026
Revision Received:	May 5, 2026
Editorial Decision:	July 16, 2026
Revision Received:	August 11, 2026
Accepted:	August 13, 2026

Editor: Evelien Adriaenssens

Reviewer(s): Disclosure of reviewer identity is with reference to reviewer comments included in decision letter(s). The following individuals involved in review of your submission have agreed to reveal their identity: Liviana Ricci (Reviewer #1)

Transaction Report:

(Note: With the exception of the correction of typographical or spelling errors that could be a source of ambiguity, letters and reports are not edited. The original formatting of letters and referee reports may not be reflected in this compilation.)

DOI: https://doi.org/10.1128/msystems.00101-26

Re: mSystems00101-26 (Global and local trajectories of infant gut microbiota development in Bangladesh across the COVID-19 pandemic)

Dear Prof. Jelle Matthijnssens:

Thank you for the privilege of reviewing your work. Below you will find my comments, instructions from the mSystems editorial office, and the reviewer comments.

Due to a lack of available reviewers, I have performed an additional review myself, independent of reviewer 1. I note the high quality of the analyses performed but the low number of individuals sampled and the lack of strain level resolution due to 16S amplicon sequencing. I therefore urge the authors to be careful in their statements, such as interpreting the impact of COVID-19 lockdowns and the importance of Segatella.
I also note the lack of page and line numbers which makes it difficult to provide a detailed review.

Comment to address:
In the COVID-19 section, the authors compare maturation scores between infants before and during lockdown. However, it is unclear whether they have taken incidence of disease (or diarrhea) into account when performing this analysis. With the individual infant responses showing different trajectories, what is the correlation with disease?

I agree with the comments of reviewer 1 and would ask the authors to revise their manuscript accordingly.

Please return the manuscript within 60 days; if you cannot complete the modification within this time period, please contact me. If you do not wish to modify the manuscript and prefer to submit it to another journal, notify me immediately so that the manuscript may be formally withdrawn from consideration by mSystems.

Revision Guidelines
To submit your modified manuscript, log into the submission site at https://msystems.msubmit.net/cgi-bin/main.plex. Go to Author Tasks and click the appropriate manuscript title to begin. The information you entered when you first submitted the paper will be displayed; update this as necessary. Note the following requirements:

• Upload point-by-point responses to the issues raised by the reviewers in a file named "Response to Reviewers," NOT in your cover letter.
• Upload a compare copy of the manuscript (without figures) as a "Marked-Up Manuscript" file.
• Upload a clean .DOC/.DOCX version of the revised manuscript and remove the previous version.
• Each figure must be uploaded as a separate, editable, high-resolution file (TIFF or EPS preferred), and any multipanel figures must be assembled into one file.
• Any supplemental material intended for posting by ASM should be uploaded with their legends separate from the main manuscript. You can combine all supplemental material into one file (preferred) or split it into a maximum of 10 files with all associated legends included.

For complete guidelines on revision requirements, see our Submission and Review Process webpage. Submission of a paper that does not conform to guidelines may delay acceptance of your manuscript.

Data availability: ASM policy requires that data be available to the public upon online posting of the article, so please verify all links to sequence records, if present, and make sure that each number retrieves the full record of the data. If a new accession number is not linked or a link is broken, provide mSystems production staff with the correct URL for the record. If the accession numbers for new data are not publicly accessible before the expected online posting of the article, publication may be delayed; please contact production staff (mSystems@asmusa.org) immediately with the expected release date.

Publication Fees: For information on publication fees and which article types are subject to charges, visit our website. If your manuscript is accepted for publication and any fees apply, you will be contacted separately about payment during the production process; please follow the instructions in that e-mail. Arrangements for payment must be made before your article is published.

ASM Membership: Corresponding authors may join or renew ASM membership to obtain discounts on publication fees. Need to upgrade your membership level? Please contact Customer Service at Service@asmusa.org.

The ASM Journals program strives for constant improvement in our submission and publication process. Please tell us how we can improve your experience by taking this quick Author Survey.

Thank you for submitting your paper to mSystems.

Sincerely,

Evelien Adriaenssens
Editor
mSystems

Reviewer #1 (Comments for the Author):

This longitudinal study investigates the development of the gut microbiota in healthy infants from urban Bangladesh (BBGUT cohort) over their first two years of life, comparing them to a Belgian cohort (BABEL). While the core microbial signatures differed-most notably through the presence of Segatella in the Bangladeshi cohort-the overall patterns of bacterial colonization remained conserved across different settings. The authors identified diarrhoeal disease and antibiotics to be associated with temporary setbacks in infant microbiome development. The study also highlights a complex link between high levels of Segatella and disease episodes. However, as acknowledged by the authors, 16S rRNA sequencing (V4 region) lacks species- and strain-level resolution -the main limitation of the study, in my opinion-to allow inference of which taxa within the highly diverse Segatella genus correlates with the evidence presented. This is a significant drawback, as different strains/clades within the Segatella genus, and the S. copri sp in particular, are known to have functionally distinct roles that cannot be distinguished here. Still, the study is well performed, with an impressive longitudinal sampling density (partly compensating for the small-sized cohort) and is valuable for the description of high level trajectories in infant microbiome development, although the findings may not be generalizable to larger populations due to small cohort size. While the COVID-19 findings require more cautious interpretation, the discovery of conserved maturation trajectories across diverse geographies and the link between Segatella and illness are significant findings that warrant further high-resolution investigation. Ultimately, the study also emphasizes the necessity of including non-Western populations to metagenomic surveys to create a more accurate framework for understanding early-life gut health and development.

Intro:
"The initial inoculation is characterized by pioneer bacteria, ... Enterobacteriaceae." Please add a supporting reference.
Same for the following sentence and the following.

Materials and Methods:
"Thirty pregnant women were enrolled at the International Centre for Diarrheal Disease Research". Does this mean they were ill/symptomatic with diarrheal disease when enrolled? Please state their health status and whether this was followed-up in months following the enrollment, including when the baby was born.
Please add a section with detailed information on how host metadata (diet, health status, antibiotic usage, etc) were collected.
I think this sentence in brackets could be improved in clarity: "children born within 1 month of each other, sampled from 26/10/2018 - 24/11/2020", e.g. children "were" born... "and the sampling occurred between X and Y dates"?
please define the acronym "ADHC"
"Fecal microbiota profiling and moisture content measurement" - Were fresh samples processed locally after collection? Or were the samples moved elsewhere and processed for delivery, until analysis? Please add adequate details, e.g. freezing -80/-20C for how long, use of buffer X, homogenization ....
kudos to the authors for re-processing the Belgian (BABEL) cohort data using the exact same pipeline as in the Bangladeshi!
please provide version and settings (e.g. default) for all softwares used in the various analyses.

Results
on Segatella: out of curiosity, would you be able to do shotgun metagenomics or PCR to identify the specific species and subspecies clade of Prevotella in Bangladeshi babies? S. copri in particular is known to display high subspecies diversity with different clades carrying different CAZymes and functional repertoires, having different geographical distributions and displaying various associations to host health status. Also, please provide reference to work renaming this genus.
Fig 1A. Please specify in the figure caption that the PCoA contains all the samples, across all timepoints, in the dataset.
In "Disease-related events are associated with maturation setbacks", the authors mention quite various host metadata but I found no introduction of these and how they were collected in the methods. As previously commented, I think this information should be extensively integrated in the material and methods section in an independent paragraph (e.g. diet information was collected via food frequency questionnaires for mothers- if done-/other for babies; collection of information on health status, how?).
The authors suggest the lockdown caused a "plateauing" or "slowdown" in maturation. However, the data shows no significant overall decrease in maturation scores ($p=0.46$) or alpha diversity indices ($p=0.43/0.44$) during the lockdown, If I haven't misunderstood this part? I think the claim of a broad "delay" or "halting" may be slightly overstated given the lack of cohort-wide statistical significance and authors should rephrase this part accordingly.
In the statement "Further analysis revealed that the observed increase in Segatella abundance was primarily associated with disease-related samples, rather than healthy ones", I'd report the supporting statistics in brackets in the main text.
In the analysis of Fig. 5, I'm curious to know whether the authors identified specific antibiotics linked to Segatella spikes and a specific decrease in one or more taxa explaining the Segatella population expansion.

Discussion
Although novel and of interest, I think the authors should tone down the results about COVID-19 and limit discussion to statistically significant evidence, particularly the speculative comments about lockdown and increased breastfeeding frequency.
"Such resolution may uncover functionally distinct variants of Segatella that differ in their impacts on host health" - Segatella phylogenetic and functional heterogeneity is well described in literature (for instance, S. copri clade A is consistently reported as associated with biomarkers of inflammation). I suggest the authors rephrase the sentence referencing adequate literature.
The authors should probably comment on the small size of the cohort enrolled in the study, limiting generalizability of the findings presented.

This longitudinal study investigates the development of the gut microbiota in healthy infants from urban Bangladesh (BBGUT cohort) over their first two years of life, comparing them to a Belgian cohort (BABEL). While the core microbial signatures differed—most notably through the presence of *Segatella* in the Bangladeshi cohort—the overall patterns of bacterial colonization remained conserved across different settings. The authors identified diarrhoeal disease and antibiotics to be associated with temporary setbacks in infant microbiome development. The study also highlights a complex link between high levels of *Segatella* and disease episodes. However, as acknowledged by the authors, 16S rRNA sequencing (V4 region) lacks species- and strain-level resolution —the main limitation of the study, in my opinion—to allow inference of which taxa within the highly diverse *Segatella* genus correlates with the evidence presented. This is a significant drawback, as different strains/clades within the *Segatella* genus, and the *S. copri* sp in particular, are known to have functionally distinct roles that cannot be distinguished here. Still, the study is well performed, with an impressive longitudinal sampling density (partly compensating for the small-sized cohort) and is valuable for the description of high level trajectories in infant microbiome development, although the findings may not be generalizable to larger populations due to small cohort size. While the COVID-19 findings require more cautious interpretation, the discovery of conserved maturation trajectories across diverse geographies and the link between *Segatella* and illness are significant findings that warrant further high-resolution investigation. Ultimately, the study also emphasizes the necessity of including non-Western populations to metagenomic surveys to create a more accurate framework for understanding early-life gut health and development.

Intro:

- “The initial inoculation is characterized by pioneer bacteria, … Enterobacteriaceae.” Please add a supporting reference.
- Same for the following sentence and the following.

Materials and Methods:

- “Thirty pregnant women were enrolled at the International Centre for Diarrheal Disease Research”. Does this mean they were ill/symptomatic with diarrheal disease when enrolled? Please state their health status and whether this was followed-up in months following the enrollment, including when the baby was born.
- Please add a section with detailed information on how host metadata (diet, health status, antibiotic usage, etc) were collected.
- I think this sentence in brackets could be improved in clarity: “children born within 1 month of each other, sampled from 26/10/2018 – 24/11/2020“, e.g. children “were” born… “and the sampling occurred between X and Y dates”?
- please define the acronym “ADHC”
- “Fecal microbiota profiling and moisture content measurement” - Were fresh samples processed locally after collection? Or were the samples moved elsewhere and processed for delivery, until analysis? Please add adequate details, e.g. freezing -80/-20C for how long, use of buffer X, homogenization ….
- kudos to the authors for re-processing the Belgian (BABEL) cohort data using the exact same pipeline as in the Bangladeshi!

- please provide version and settings (e.g. default) for all softwares used in the various analyses.

Results

- on *Segatella*: out of curiosity, would you be able to do shotgun metagenomics or PCR to identify the specific species and subspecies clade of Prevotella in Bangladeshi babies? *S. copri* in particular is known to display high subspecies diversity with different clades carrying different CAZymes and functional repertoires, having different geographical distributions and displaying various associations to host health status. Also, please provide reference to work renaming this genus.
- Fig 1A. Please specify in the figure caption that the PCoA contains all the samples, across all timepoints, in the dataset.
- In “Disease-related events are associated with maturation setbacks”, the authors mention quite various host metadata but I found no introduction of these and how they were collected in the methods. As previously commented, I think this information should be extensively integrated in the material and methods section in an independent paragraph (e.g. diet information was collected via food frequency questionnaires for mothers- if done-/other for babies; collection of information on health status, how?).
- The authors suggest the lockdown caused a "plateauing" or "slowdown" in maturation. However, the data shows no significant overall decrease in maturation scores (p=0.46) or alpha diversity indices (p=0.43/0.44) during the lockdown, If I haven’t misunderstood this part? I think the claim of a broad "delay" or "halting" may be slightly overstated given the lack of cohort-wide statistical significance and authors should rephrase this part accordingly.
- In the statement “Further analysis revealed that the observed increase in Segatella abundance was primarily associated with disease-related samples, rather than healthy ones”, I’d report the supporting statistics in brackets in the main text.
- In the analysis of Fig. 5, I’m curious to know whether the authors identified specific antibiotics linked to *Segatella* spikes and a specific decrease in one or more taxa explaining the *Segatella* population expansion.

Discussion

- Although novel and of interest, I think the authors should tone down the results about COVID-19 and limit discussion to statistically significant evidence, particularly the speculative comments about lockdown and increased breastfeeding frequency.
- *“*Such resolution may uncover functionally distinct variants of *Segatella* that differ in their impacts on host health*”* - *Segatella* phylogenetic and functional heterogeneity is well described in literature (for instance, *S. copri* clade A is consistently reported as associated with biomarkers of inflammation). I suggest the authors rephrase the sentence referencing adequate literature.
- The authors should probably comment on the small size of the cohort enrolled in the study, limiting generalizability of the findings presented.

Dear Dr. Evelien M. Adriaenssens,
Dear Editor,

We are pleased to submit the revised version of our manuscript (mSystems00101-26) titled "*Global and local trajectories of infant gut microbiota development in Bangladesh across the COVID-19 pandemic*".

We sincerely thank the editor and reviewer 1 for their thorough evaluation and constructive comments on our work. As requested, we have addressed each comment in a detailed, point-by-point response below, and have revised the manuscript accordingly. In doing so, we have made minor adjustments throughout the text to improve clarity where appropriate and have updated Prof. Dr. Sara Vieira-Silva's affiliation to correspond with her current position.

EDITOR comments:

1. I note the high quality of the analyses performed but the low number of individuals sampled and the lack of strain level resolution due to 16S amplicon sequencing. I therefore urge the authors to be careful in their statements, such as interpreting the impact of COVID-19 lockdowns and the importance of Segatella.
I also note the lack of page and line numbers which makes it difficult to provide a detailed review.

Response 1: We thank the editor for their interest in our work and their valuable remarks. We acknowledge that the use of 16S rRNA amplicon sequencing limits taxonomic resolution and that the study includes a relatively small number of individuals. However, as also noted by reviewer 1, this last limitation is partly mitigated by the dense longitudinal sampling, which results in a robust and well-characterized dataset for each individual, making it comparable to other longitudinal infant gut microbiome studies such as Beller at al. (2021)[1]. Especially for non-Western cohorts, such densely sampled longitudinal studies are still uncommon, and we hope that our dataset can provide additional context for understanding infant gut microbiota development in these populations.

[1]Beller L, Deboutte W, Falony G, et al. Successional Stages in Infant Gut Microbiota Maturation. *mBio*. 2021;12(6):e0185721. doi:10.1128/mBio.01857-21

Action taken: We have revised the manuscript to moderate our interpretations, particularly regarding the impact of the COVID-19 lockdown to address all comments accordingly. The modifications regarding the COVID-19 section are described in more detail in response to the reviewer 1's comments 13 and 16.
Page and line numbers have also been added in the revised version of the manuscript.

We appreciate the editor's and reviewer's thoughtful feedback, which has helped us improve the clarity and quality of our manuscript. We hope that the revised version meets the journal's expectations and look forward to its consideration for publication in *mSystems*.

2. In the COVID-19 section, the authors compare maturation scores between infants before and during lockdown. However, it is unclear whether they have taken incidence of disease (or diarrhea) into account when performing this analysis. With the individual infant responses showing different trajectories, what is the correlation with disease?

Response 2: We thank the editor for this important comment and agree that the text lacked clarity regarding the type of samples used in this part of the analysis. In this analysis, we did not account for disease incidence when comparing maturation scores before and during the COVID-19 lockdown. This is because the samples analyzed from the second year of life, on which this comparison is based, predominantly represent healthy time points.

In our experimental design, longitudinal sampling in the first year included both healthy and disease-associated time points. However, sampling in the second year was less dense, and most samples analyzed for this study were collected at healthy time points (we sequenced approximately one sample per child per month), with only a very limited number of samples coinciding with disease episodes (for more details on the experimental design please see the "Study design" section in Supplementary Extended Methods).

As a result, the number of disease-associated samples in the second year is too small (disease associated samples: n=17, healthy time point samples: n=158) and unevenly distributed across individuals to support a robust analysis of the relationship between disease incidence and maturation scores. Therefore, no correlation analysis with disease was performed for this period.

We acknowledge that disease occurrence may influence individual maturation trajectories and contribute to variability, potentially explaining the different trajectories observed across individuals in Figure 4c. However, given the limitations of the available data, this could not be formally assessed in our study.

Action taken: To address this comment and improve clarity in the COVID-19 section, we have revised the text at the following parts (changes highlighted in bold):

Lines *487-491*: *"*Thus, we divided the months of the second year into two periods: pre-lockdown (months 13-16 corresponding to infant age) and lockdown (months 17-24 corresponding to infant age). **For this analysis we focused on samples representing healthy time points due to a limited number of samples associated with disease in year 2 (n=17/175 samples).***"*

Lines *499-500*: *"*Next, we compared maturation scores between infants before and during lockdown **(calculated based on HTP samples)**, accounting for bacterial rankings and presence across monthly samples throughout their second year of life as previously discussed (Fig 3b)."

Lines *518-519*: "Fig. 4c. …. Each point represents an infant's maturation score in each period **calculated considering HTP samples only**, with colors distinguishing different children."

REVIEWER #1 comments:

1. This longitudinal study investigates the development of the gut microbiota in healthy infants from urban Bangladesh (BBGUT cohort) over their first two years of life, comparing them to a Belgian cohort (BABEL). While the core microbial signatures differed-most notably through the presence of Segatella in the Bangladeshi cohort-the overall patterns of bacterial colonization remained conserved across different settings. The authors identified diarrhoeal disease and antibiotics to be associated with temporary setbacks in infant microbiome development. The study also highlights a complex link between high levels of Segatella and disease episodes. However, as acknowledged by the authors, 16S rRNA sequencing (V4 region) lacks species- and strain-level resolution -the main limitation of the study, in my opinion-to allow inference of which taxa within the highly diverse Segatella genus correlates with the evidence presented. This is a significant drawback, as different strains/clades within the Segatella genus, and the S. copri sp in particular, are known to have functionally distinct roles that cannot be distinguished here. Still, the study is well performed, with an impressive longitudinal sampling density (partly compensating for the small-sized cohort) and is valuable for the description of high level trajectories in infant microbiome development, although the findings may not be generalizable to larger populations due to small cohort size. While the COVID-19 findings require more cautious interpretation, the discovery of conserved maturation trajectories across diverse geographies and the link between Segatella and illness are significant findings that warrant further high-resolution investigation. Ultimately, the study also emphasizes the necessity of including non-Western populations to metagenomic surveys to create a more accurate framework for understanding early-life gut health and development.

Response 1: We thank the reviewer for the thoughtful summary and constructive evaluation of our work. We appreciate the positive remarks and are pleased that both the editor and reviewer found merit in our work. We are also grateful for the reviewer's observations regarding the limitations of our study, which we found very helpful for the improvement of the manuscript.

In response, we have carefully considered each of the reviewer's comments and addressed them point-by-point below.

We are thankful for the time and effort invested in this evaluation and we hope that the revisions adequately address the reviewer's concerns.

Intro:

2. "The initial inoculation is characterized by pioneer bacteria, ... Enterobacteriaceae." Please add a supporting reference. Same for the following sentence and the following.

Response 2: We thank the reviewer for pointing this out. To address this matter, we have added appropriate references to support the following statements:

Lines *107-108*: "The initial inoculation is characterized by pioneer bacteria, primarily facultative anaerobes such as *Enterobacteriaceae*."

Action taken (references added):
Xiao L, Zhao F. Microbial transmission, colonisation and succession: from pregnancy to infancy. Gut. 2023;72(4):772-786. doi:10.1136/gutjnl-2022-328970

Lines *108-109*: "If breastfeeding takes place, fermentative bacteria, such as *Bifidobacterium,* become dominant, facilitating the digestion of human milk oligosaccharides (HMOs)."

Action taken (reference added):
Xiao L, Zhao F. Microbial transmission, colonisation and succession: from pregnancy to infancy. Gut. 2023;72(4):772-786. doi:10.1136/gutjnl-2022-328970

Lines *109-111*: "As infants transition to solid foods, microbial diversity expands, with key taxa like *Bacteroidota*, *Lachnospiraceae*, and *Ruminococcaceae* emerging to metabolize complex carbohydrates."

Action taken (reference added):
Nunez H, Nieto PA, Mars RA, Ghavami M, Sew Hoy C, Sukhum K. Early life gut microbiome and its impact on childhood health and chronic conditions. Gut Microbes. 2025;17(1):2463567. doi:10.1080/19490976.2025.2463567

Xiao L, Zhao F. Microbial transmission, colonisation and succession: from pregnancy to infancy. Gut. 2023;72(4):772-786. doi:10.1136/gutjnl-2022-328970

A small remark: The reference numbering in the revised manuscript has also been updated based on all reference-related modifications made.

Materials and Methods:

3. "Thirty pregnant women were enrolled at the International Centre for Diarrheal Disease Research". Does this mean they were ill/symptomatic with diarrheal disease when enrolled? Please state their health status and whether this was followed-up in months following the enrollment, including when the baby was born.

Response 3: We thank the reviewer for highlighting this point, as this information was not clearly stated in the original text. We have revised the manuscript accordingly to clarify the health status of the mothers at enrollment and provide additional details on maternal health data collected during the study.

Action taken: We have included additional information in the Supplementary Extended Methods document. More specifically the following modifications were made (in bold):

Lines *2 - 14*: **"Thirty pregnant women were enrolled at the International Centre for Diarrheal Disease Research, Bangladesh (icddr,b) for the purposes of this study. All participating women were healthy and in the third trimester of their pregnancy at the time of enrollment. Individuals younger than 18 years of age or with major underlying conditions such as cancer, uncontrolled hypertension, or mental illness were excluded from participation. At the first household visit, information on birth characteristics (mode and place of delivery, gestational age) and overall maternal health during the final trimester was collected to confirm that no serious adverse events had occurred. No additional maternal health data were collected on the day of delivery, or in the postpartum period.**
Longitudinal metadata for this study was collected exclusively for the infants and included demographic information (birth mode/birth place/household composition), medical history information (vaccination/antibiotics/medication/disease occurrence) as well as dietary details (breastfeeding/formula feeding/solid food) (Supplementary Table 1a)."

A minor clarification has also been added in the "Study design and sample selection" subsection of the Materials and Methods section in the main manuscript:

Line *149*: "**Infant** fecal material collection occurred three times/week during the first six months, twice/week in the following six months, and once/week throughout the second year."

4. Please add a section with detailed information on how host metadata (diet, health status, antibiotic usage, etc) were collected.

<u>Response 4</u>: We recognize the importance of the reviewer's comment and agree that detailed reporting of metadata collection is essential for better comprehension of our work. In response, we have provided a more detailed description of the metadata collection procedures (including diet, health status, and antibiotic usage) in Supplementary Extended Methods. Given the word number constraints, we have included a brief explanatory statement in the main text, introducing the metadata collected and directing the reader to the supplementary material for full details.

<u>Action taken</u>: We revised the manuscript by adding more information in the following sections:

<u>Supplementary Extended Methods - "Study design" section:</u>
Lines *11-24*: "Longitudinal metadata for this study was collected exclusively for the infants and included demographic information (birth mode/birth place/household composition), medical history information (vaccination/antibiotics/medication/disease occurrence) as well as dietary details (breastfeeding/formula feeding/solid food) (Supplementary Table 1a). **All metadata was collected using structured questionnaires administered to the mothers by trained field staff during the household visits for sample collection and transportation. These visits were conducted as frequently as sampling occurred, and information was obtained via maternal report using standardized questionnaire responses, covering both the day of sample collection and the interval since the previous visit. Specifically, medical history questions asked which vaccines, antibiotics, or other medications the infant had received, including timing, duration, and reason for administration. For disease occurrence, mothers reported any illnesses their child had experienced or was experiencing since the previous visit. Dietary questions captured whether breastfeeding occurred and its frequency, the use of formula, and whether the infant had begun consuming solid foods."**

<u>Main manuscript - "Study design and sample selection" subsection of the Materials and Methods section:</u>
Lines *154-162*: "Trained field staff conducted household visits for sample collection and transport to the laboratory, during which detailed metadata questionnaires were completed. **These questionnaires were designed to collect longitudinal infant-specific metadata, including demographic characteristics (birth mode, birth place, and household composition), medical history (vaccination status, antibiotic/medication exposure, and disease occurrence), and dietary practices (breastfeeding, formula feeding, and solid food introduction) (Supplementary Table 1a). Further details on metadata collection are provided in Supplementary Extended Methods.**"

<u>5.</u> I think this sentence in brackets could be improved in clarity: "children born within 1 month of each other, sampled from 26/10/2018 - 24/11/2020", e.g. children "were" born... "and the sampling occurred between X and Y dates"?

<u>Response 5</u>: We agree with the reviewer that the original sentence was not sufficiently clear and have revised it as follows:

Lines *146-149*: "Thirty **healthy** pregnant women were enrolled at the International Centre for Diarrheal Disease Research, Bangladesh (icddr,b) and their newborns were followed from birth over a two-year period (**children were born between 26/10/2018-01/12/2018, and sampling occurred between 26/10/2018 – 24/11/2020**)."

<u>6.</u> Please define the acronym "ADHC"

<u>Response 6</u>: We recognize the lack of clarity in the definition of the acronym and have now clarified it as follows:

Lines *170-171*: "For this, 498 (out of 984) *ad hoc* collected samples (i. e., **collected on an *ad hoc* basis,** ADHC samples) were selected (Supplementary Fig. 2)."

<u>7.</u> "Fecal microbiota profiling and moisture content measurement" - Were fresh samples processed locally after collection? Or were the samples moved elsewhere and processed for delivery, until analysis? Please add adequate details, e.g. freezing -80/-20C for how long, use of buffer X, homogenization ....

<u>Response 7</u>: We thank the reviewer for this important remark, and we acknowledge that this information was not sufficiently presented in the manuscript. To address this, we incorporated some changes to improve the manuscript's "Fecal microbiota and moisture content measurement" section as well as the "Study design" and "Fecal microbiota profiling" sections in Supplementary Extended methods. We would also like to point out that the cold chain was maintained throughout sample transport, and storage by keeping samples at controlled low temperatures to prevent degradation.

<u>Action taken</u>: The following modifications/additions were made (in bold):

<u>Main manuscript - Fecal microbiota profiling and moisture content measurement section:</u>

Lines *176-177*: **"Frozen samples were aliquoted (150–200 mg) at the Rega Institute (Belgium) without thawing and further maintained at −80 °C until DNA extraction**. Bacterial DNA extraction…"

<u>Supplementary Extended Methods - Study design section:</u>

Lines *27-39*: In total 5,082 samples **were collected** throughout the sampling period. **Fresh samples were collected by the mothers in pre-labeled tubes and transported on the same day to the International Centre for Diarrheal Disease Research, Bangladesh (icddr,b) by trained field staff.** During the COVID-19 pandemic lockdown in Bangladesh (March-November 2020), samples were stored at -20°C in freezers at the participants' homes and **were** collected **and transported** once a week or biweekly, while metadata was collected over the phone. **Upon delivery to the Virology laboratory at the International Centre for Diarrheal Disease Research, Bangladesh (icddr,b),** all fresh samples were aliquoted in capped O-ring 2mL tubes and stored at -80°C until shipment to the Rega Institute laboratory of Viral Metagenomics in Belgium. Samples were shipped **on dry ice** in two batches (first year samples shipped in December 2019, second year samples shipped in January 2021) and stored at -80°C until further processing.

Supplementary Extended Methods - Fecal microbiota profiling section):
Lines *89-92*: **All fecal samples were further aliquoted at the Rega Institute (Belgium) and weighed (150–200 mg) while maintained in a frozen state to ensure accurate sample input prior DNA extraction.** For this part of the analysis, one aliquot was used for the laboratory analysis, and one was kept as backup at **-80°C**.

8. kudos to the authors for re-processing the Belgian (BABEL) cohort data using the exact same pipeline as in the Bangladeshi!

Response 8: We appreciate the reviewer's positive comment and kind words on this aspect of the work.

9. Please provide version and settings (e.g. default) for all softwares used in the various analyses.

Response 9: We thank the reviewer for pointing this out. The "Statistical analyses" subsection in both the main manuscript (Materials and Methods) and the Supplementary Extended Methods has now been revised accordingly.

Action taken: Software versions and setting details have been included in the following lines (highlighted in bold):

Main manuscript – "Statistical analyses":

Lines *205-224*: "All statistical analyses and visualization were conducted in R (http://www.R - project.org, **v4.3.2**), using the following packages: *ggplot2*[30]**(v3.4.4)**, *vegan*[31]**(v2.6-4)**, *phyloseq*[32]**(v1.42.0)**, *microbiome*[33]**(v1.20.0)** and *microViz*[34]**(v0.11.0)**. To analyze group differences and temporal trends in alpha diversity, linear mixed-effects models were used with the *lme4* **(v1.1-37)** and *lsmeans* **(v2.30-2)** R packages. These models account for the non-

independence of repeated measurements from the same infants by including subject-level random intercepts. P-values were computed using likelihood ratio tests for overall effects and Kenward-Roger adjusted Wald tests for pairwise group comparisons. To compare median differences between independent or paired groups of continuous variables, we used the Wilcoxon rank-sum (or Mann-Whitney U) and Wilcoxon signed-rank tests for two groups. To evaluate the association between genera prevalence patterns across the Bangladeshi and the Belgian infant cohorts, a Spearman rank correlation analysis was computed. Differences in microbial community composition (beta diversity) between cohorts were assessed using permutational multivariate analysis of variance (PERMANOVA) with the adonis2 function in the *vegan* R package **(Bray-Curtis dissimilarity, 999 permutations)**. **Differences in *Segatella* prevalence between sample groups across gut microbiota maturation stages were assessed using Fisher's exact tests while Pearson's chi-squared tests were** conducted on contingency tables that were created to assess the association between different covariates and (a) the occurrence of maturation setbacks, (b) the presence of samples with high *Segatella* abundance. Where applicable, multiple testing adjustments were made using the Benjamini-Hochberg method, with a false discovery rate (FDR) threshold set at 0.05.

Supplementary Extended Methods – "Statistical analyses" section):

Lines *112-156*: Dirichlet multinomial mixture modeling (DMM) for identification of bacterial communities

Community typing was performed by applying a Dirichlet multinomial mixture modeling (DMM) approach [35] to the 16S rRNA gene dataset in order to explore the presence of bacterial sub-communities within the Bangladeshi infant cohort. For this purpose, we used the *DirichletMultinomial* R package [36] **(v1.40.0)** on the rarefied table agglomerated at the genus level. The best number of clusters was determined based on the Bayesian information criterion (BIC) and every sample was assigned to one (Supplementary Table 1).

Order of appearance of most abundant bacterial genera

The most abundant genera in the cohort were defined as being present in more than 25% of the infants. Genera were then ranked based on the day in which they first appeared with a relative abundance exceeding 0.5%. Rankings were assessed using the Kendall W test with 10,000 permutations, applying the kendall.w function from the R *synchrony* package [37] **(v0.3.8)**. The final sequence of appearance was established by ordering the median ranks for each infant.

Diversity metrics and multivariate analysis of metadata effects on the microbiota

Alpha diversity (observed genus richness and Shannon diversity) and beta diversity (Bray-Curtis dissimilarity) metrics were calculated using the *microbiome* **(v1.20.0)** and *phyloseq* **(v1.42.0)** packages and genus-level ordinations were visualized using Principal-coordinate analysis (PcoA) computed from Bray-Curtis dissimilarities. To determine the contribution of metadata covariates

on microbiota variation, we conducted a univariate distance-based redundancy analysis (dbRDA) at the genus level using Bray-Curtis distances and the capscale function from the *vegan* R package **(v2.6-4)**. Significant covariates were identified and then refined with forward model selection (stepwise dbRDA) via the ordiR2step function in *vegan* **with 1000 permutations per step**, providing a nonredundant cumulative assessment of metadata contributions. The envfit function from the *vegan* package was used to fit metadata variables onto the PCoA ordination, with effect sizes shown as arrows. To examine the influence of metadata on specific genera, an analogous approach was used: the community was narrowed to focus on each of the top 15 genera, with dbRDA performed on Euclidean distances based on genus abundance, followed by forward model selection, while controlling for infant ID.

Results

10. On Segatella: out of curiosity, would you be able to do shotgun metagenomics or PCR to identify the specific species and subspecies clade of Prevotella in Bangladeshi babies? S. copri in particular is known to display high subspecies diversity with different clades carrying different CAZymes and functional repertoires, having different geographical distributions and displaying various associations to host health status. Also, please provide reference to work renaming this genus.

Response 10: We thank the reviewer for this insightful comment on the taxonomic and functional complexity of the *Segatella copri* complex (ScC). We agree that shotgun metagenomics would provide the highest resolution for strain-level analyses and CAZyme profiling. However, we are currently limited to V4 16S ASVs, which, as acknowledged above, cannot reliably distinguish ScC species. We note this as a limitation of the study and suggest it as an important direction for future work.

While we fully recognize the value of new metagenomic or clade-specific PCR data, generating such data falls beyond the scope of the present study and cannot be accommodated within the current revision timeline. Nonetheless, to address the reviewer's point using the data already generated, we performed an exploratory analysis based on 16S V4 ASV sequences. Using diagnostic hypervariable motifs described in the literature (Hitch et al., 2022; Tett et al., 2019; Blanco-Míguez et al., 2023) we tentatively assigned Segatella ASVs to known clades (A–G) and evaluated these assignments using a Neighbor-Joining phylogenetic reconstruction. This analysis suggested that most Segatella ASVs clustered within the S. copri Clade A/C group, with additional ASVs corresponding to Clade B and S. hominis (Clade E), Figure 1.
Given the limited resolving power of the V4 region, particularly for distinguishing closely related clades such as A versus C, we consider these assignments exploratory. For this reason, we have chosen to retain the original analysis and to report these ASVs conservatively as corresponding to Clades A/C, B, and E.

However, in line with the editor's suggestion of caution in interpreting *Segatella*-related findings, and given the limitations of our dataset, we have chosen not to include these results in the revised manuscript.

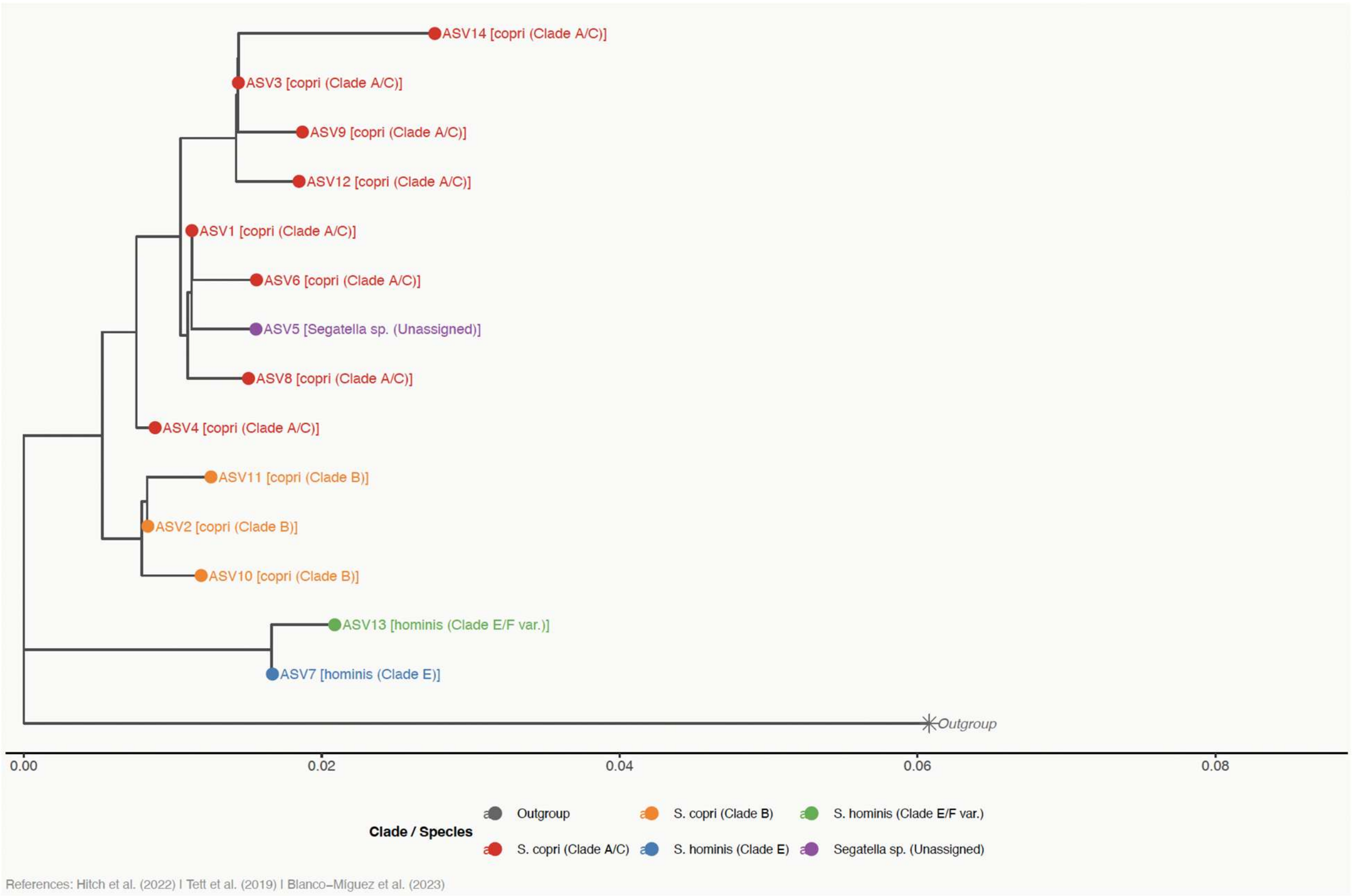

Fig. 1. Phylogenetic Structure of Segatella ASVs
Neighbor-Joining tree based on K80 distances of 16S rRNA V4 amplicon sequences. Tip labels indicate Amplicon Sequence Variants (ASVs) and exploratory clade assignments based on diagnostic hypervariable motifs. Colors represent distinct Segatella clades, with the majority of ASVs (n=8) clustering within the *S. copri* Clade A/C group. Nomenclature follows the genus reclassification by Hitch et al. (2022). Scale bar represents nucleotide substitutions per site.

Hitch TCA, Bisdorf K, Afrizal A, et al. A taxonomic note on the genus Prevotella: Description of four novel genera and emended description of the genera Hallella and Xylanibacter. *Syst Appl Microbiol*. 2022;45(6):126354. doi:10.1016/j.syapm.2022.126354

Tett A, Huang KD, Asnicar F, et al. The Prevotella copri Complex Comprises Four Distinct Clades Underrepresented in Westernized Populations. *Cell Host Microbe*. 2019;26(5):666-679.e7. doi:10.1016/j.chom.2019.08.018

Blanco-Míguez A, Gálvez EJC, Pasolli E, et al. Extension of the Segatella copri complex to 13 species with distinct large extrachromosomal elements and associations with host conditions. *Cell Host Microbe*. 2023;31(11):1804-1819.e9. doi:10.1016/j.chom.2023.09.013

11. Fig 1A. Please specify in the figure caption that the PCoA contains all the samples, across all timepoints, in the dataset.

Response 11: We thank the reviewer for highlighting this. To improve clarity, we have updated the Figure 1A caption as follows:

Lines *302-303*: "Principal coordinate analysis (PCoA) showing the clustering of **all** infant **(n=984)** and maternal samples **(n = 18)**."

12. In "Disease-related events are associated with maturation setbacks", the authors mention quite various host metadata but I found no introduction of these and how they were collected in the methods. As previously commented, I think this information should be extensively integrated in the material and methods section in an independent paragraph (e.g. diet information was collected via food frequency questionnaires for mothers- if done-/other for babies; collection of information on health status, how?).

Response 12: We thank the reviewer for this valuable comment. We agree with this feedback and recognize the importance of transparently reporting the metadata and clearly describing how it was collected, as this is essential for the interpretation and reproducibility of the study. As also addressed above, in response to the reviewer's comment 4, we have revised the manuscript to introduce the relevant metadata in the main Materials and Methods section. However, due to the journal's word limitations, we refrained from expanding this section extensively in the main text. Therefore, we have included a more detailed description of the metadata collection procedures in an independent paragraph in the Supplementary Extended Methods (for more details, please see Response 4).

13. The authors suggest the lockdown caused a "plateauing" or "slowdown" in maturation. However, the data shows no significant overall decrease in maturation scores (p=0.46) or alpha diversity indices (p=0.43/0.44) during the lockdown, If I haven't misunderstood this part? I think the claim of a broad "delay" or "halting" may be slightly overstated given the lack of cohort-wide statistical significance and authors should rephrase this part accordingly.

Response 13: We thank the reviewer for this suggestion and have rephrased these statements to ensure a more cautious tone in the interpretation of the lockdown effect on gut microbiota maturation.

Action taken: Specifically, we modified the following sections in the main manuscript text:

The section below:
"Although there was no significant overall decrease in the maturation score during the lockdown period (Fig. 4c), individual responses varied, with some infants showing signs of plateaued or reduced maturation. Furthermore, alpha diversity indices showed no significant change between the pre-lockdown and lockdown periods, indicating a potential slowdown or even halting in the microbial diversification process during the second year (linear mixed model, *p*=0.44 [Shannon index], *p*=0.43[observed richness]; Supplementary Table 2). These findings suggest a potential delay in microbiota maturation associated with the COVID-19 lockdown period."

Was modified as follows:
Lines *501-508*: "Although there was no significant overall decrease in the maturation score during the lockdown period (Fig. 4c), **and alpha diversity indices similarly showed no significant change between the pre-lockdown and lockdown periods (linear mixed model, *p*=0.44 [Shannon index], *p*=0.43 [observed richness]; Supplementary Table 2)**, individual responses varied, with some infants showing signs of plateaued or reduced maturation. **These results do not support a consistent, cohort-wide delay in microbiota maturation during the lockdown period (Fig. 4b). However, we identified temporal perturbations during this period, as reflected by changes in the relative abundance of specific bacterial taxa (Fig. 4c).**"

In line with this comment, we also revised the <u>abstract</u> accordingly:

The following sentence in the abstract:
"The second year of this cohort coincided with the onset of the COVID-19 pandemic and was associated with slower increase in alpha diversity and maturation, as several key taxa were less abundant than expected."

Was removed and replaced with the following:
Lines *70-72*: **"The second year of this cohort coincided with the onset of the COVID-19 pandemic where differences in the relative abundance of several key taxa were detected."**

<u>14.</u> In the statement "Further analysis revealed that the observed increase in Segatella abundance was primarily associated with disease-related samples, rather than healthy ones", I'd report the supporting statistics in brackets in the main text.

<u>Response 14:</u> We thank the reviewer for noting this. To compare the prevalence of *Segatella* between disease and healthy groups, we performed Fisher's exact tests within each maturation stage. We observed a statistically significant difference only in maturation stage C, where *Segatella* prevalence was higher in disease-related samples compared to samples representing

healthy time points. No significant differences were detected in the other maturation stages (GMMs).

Action taken: We have revised the original statement in the manuscript:

"Further analysis revealed that the observed increase in Segatella abundance was primarily associated with disease-related samples, rather than healthy ones."

and modified it as follows:
Lines *542-544*: "**Further analysis revealed that the observed increase in Segatella prevalence was primarily driven by disease-associated samples in maturation stage C (Fisher's exact test, p < 0.001, Supplementary Fig. 13a,b)**".

In addition, the following sentence was added in the caption of Supplementary Figure 13:
"**Comparing the prevalence of Segatella between disease- and healthy-associated samples, a statistically significant difference was observed only in maturation stage C (Fisher's exact test; stage A: p = 0.628; stage B: p = 0.628; stage C: p = 3.24×10^{-7}).**"
Supplementary Figure 13a was also updated to indicate the statistically significant different groups.

15. In the analysis of Fig. 5, I'm curious to know whether the authors identified specific antibiotics linked to Segatella spikes and a specific decrease in one or more taxa explaining the Segatella population expansion.

Response 15: We appreciate this insightful comment and we thank the reviewer for raising these points.
a) Regarding the potential link between antibiotic administration and *Segatella* spikes: we identified a total of 33 events with high *Segatella* abundance. Of these, 11 coincided with antibiotic use. Among these cases, Penicillin was administered in 7 instances, while other antibiotics were given in 4 cases (Macrolide = 1; Macrolide + Cephalosporin = 1; Nitroimidazole = 1; Cephalosporin = 1).

Unfortunately, given the limited number of observations, we could not perform a robust statistical analysis to assess associations with specific antibiotic classes.

b) To address the reviewer's second point, we explored compositional changes associated with high *Segatella* abundance (HSA) events by performing a within-individual analysis in which for each HSA event, the HSA representative sample was matched to the closest preceding HTP sample (sample representing a healthy time point) from the same individual. This allowed us to compare

each HSA event representative sample against the most recent available reference sample representing a healthy time point, while accounting for inter-individual differences.

We tested for differences in relative abundance of different bacterial genera between paired sample HSA and HTP groups (using Wilcoxon signed-rank tests with Benjamini–Hochberg correction) and found that *Bifidobacterium* was significantly reduced during HSA events compared to the matched HTP samples (p<0.001).

We note that in some cases the closest available HTP sample occurred up to approximately one month prior to the corresponding HSA sample and may therefore not fully capture a "pre-*Segatella*-spike" state. However, given that all samples included in this analysis correspond to time points beyond early infancy (≥ ~6 months of age), when the gut microbiota is generally expected to be less unstable than in early life, these preceding HTP samples are likely to represent a reasonable approximation of a "pre-*Segatella*-spike" state.

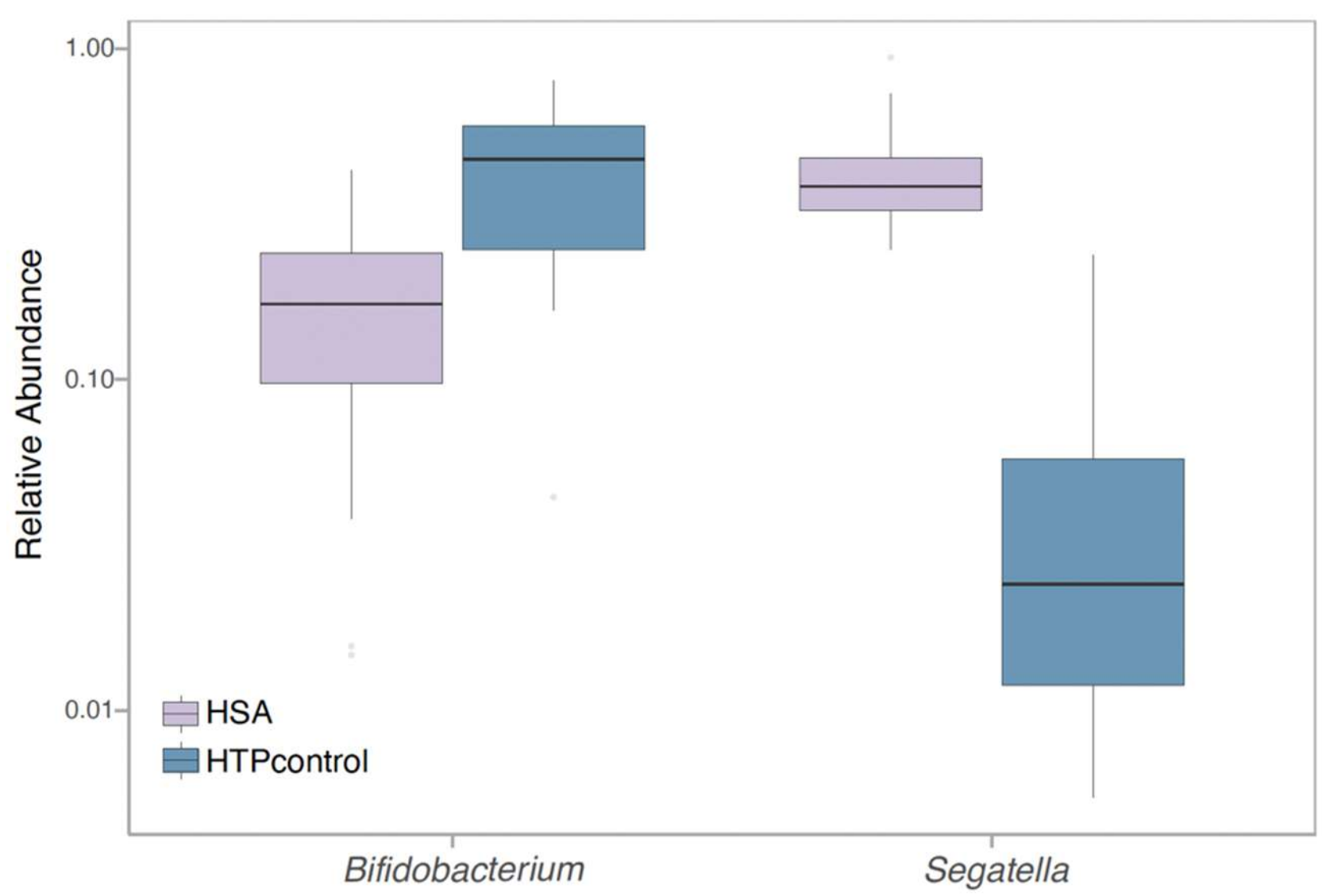

Fig 2. Boxplots showing the relative abundance of bacterial genera that differ significantly between the HSA and HTPcontrol (samples corresponding to the most recent HTP sample prior to the HSA) sample groups (paired Wilcoxon signed-rank tests, p < 0.001, adjusted for multiple comparisons using the Benjamini–Hochberg (BH) method).

<u>*Action taken*</u>: The following statements were added in the "Results", "Discussion" and Supplementary Extended Methods "Samples with high Segatella abundance (HSA)" sections:

<u>In "Results":</u>

Lines *563-567*: "**Within-individual comparison of HSA event samples against the closest preceding healthy time point (n = 33 paired events from 16 infants) identified**

***Bifidobacterium* as the only genus with a significant reduction in relative abundance during HSA events (Wilcoxon signed-rank test, adjusted $p < 0.001$, Supplementary Fig. 15)**."

In "Discussion":

Lines *692-698*: "**In the developing infant gut, a *Bifidobacterium*-dominated community is progressively displaced by Bacteroidetes—including *Bacteroides* and *Prevotella/Segatella* clade members as complementary feeding is introduced and dietary complexity increases (Vatanen et al. 2022; Olm et al. 2022). High *Segatella* abudance (HSA) events in our cohort superficially resemble this trajectory, with Segatella expanding while Bifidobacterium contracts; however, the extent to which this reflects a disease-associated perturbation remains unclear, and further work is required to resolve the underlying community dynamics**."

In the Supplementary Extended Methods "Samples with high Segatella abundance (HSA)" section:

Lines *167-172*: "**We performed a within-individual paired analysis to assess compositional changes associated with high Segatella abundance (HSA) events. For each HSA representative sample, the closest preceding healthy time point (HTP) sample from the same individual was selected as a matched reference, and paired differences in bacterial genus relative abundance between HSA and HTP samples were tested using Wilcoxon signed-rank tests with Benjamini–Hochberg correction for multiple comparisons**."

In addition, the results presented in response to this comment (Fig. 2), are now included in Supplementary figures as **Supplementary figure 15**.

Vatanen T, Ang QY, Siegwald L, et al. A distinct clade of Bifidobacterium longum in the gut of Bangladeshi children thrives during weaning. *Cell*. 2022;185(23):4280-4297.e12. doi:10.1016/j.cell.2022.10.011

Olm MR, Dahan D, Carter MM, et al. Robust variation in infant gut microbiome assembly across a spectrum of lifestyles. *Science*. 2022;376(6598):1220-1223. doi:10.1126/science.abj2972

Discussion

16. Although novel and of interest, I think the authors should tone down the results about COVID-19 and limit discussion to statistically significant evidence, particularly the speculative comments about lockdown and increased breastfeeding frequency.

Response 16: We thank the reviewer for bringing this to our attention. In response, we have revised the "Discussion" section to focus only on statistically significant findings regarding the COVID-19 section statements. We have retained the statements regarding lockdown and

breastfeeding frequency but have clarified that this interpretation is speculative and not directly supported by our data, and we have explicitly framed it as a hypothesis for future research.

Action taken: We modified the "Discussion" paragraph below:

"Sampling in the BBGUT cohort partially overlapped with the early COVID-19 lockdown measures in Bangladesh, resulting in most of the second year being spent under restrictions. To our knowledge, this is the first longitudinal study reporting potential pandemic effects on healthy infant gut microbiota development in Bangladesh and the first to examine these effects within the same cohort. During this period, we observed a reduction in relative abundance of genera associated with microbiota maturation, such as *Segatella* and *Megasphaera*. Although our results indicate a substantial presence of *Bacillota* in the first year of life, we do not observe the expected bacterial diversification and progressive increase in *Bacteroidota* abundances (formerly *Bacteroidetes*[64]) during the second year, usually observed in Bangladeshi[22] and other cohorts[11,42,58,72]. Here, we observed a plateauing in maturation scores after the implementation of lockdown measures, further supporting a potential influence of pandemic-related restrictions on microbiota development. Several factors may account for the observed deviation from the expected maturation trajectory during the second year. Lockdown-driven lifestyle and dietary changes related to COVID-19 may have contributed to delayed microbiota maturation. Extended breastfeeding throughout both years may also explain this observation, especially if breastfeeding frequency was increased during the lockdown period. The lower intestinal pH in breastfed infants may inhibit the colonization of acid-sensitive bacteria, thereby shaping microbial composition, preventing pathogen growth and potentially constraining overall microbial diversity[1,73] even after solid food introduction. This effect may be more pronounced during periods of social distancing, limiting horizontal bacterial transmission. Furthermore, we cannot exclude that changes in the sample handling upon collection during this period, might have also affected the observed compositional profiles."

To now read as follows:

Lines *649-680*: "Sampling in the BBGUT cohort partially overlapped with the early COVID-19 lockdown measures in Bangladesh, resulting in most of the second year being spent under restrictions. To our knowledge, this is the first longitudinal study reporting potential pandemic effects on healthy infant gut microbiota development in Bangladesh and the first to examine these effects within the same cohort. **Although no cohort-wide changes in gut microbiota maturation were observed during the lockdown period, taxon-specific changes were detected.** During this period, we observed a reduction in relative abundance of genera associated with microbiota maturation, such as *Segatella* and *Megasphaera*. Although our results indicate a substantial presence of *Bacillota* in the first year of life, we do not observe the expected bacterial diversification and progressive increase in *Bacteroidota* abundances (formerly *Bacteroidetes*[64])

during the second year, usually observed in Bangladeshi[22] and other cohorts[11,42,58,72]. **Several factors may account for these observations such as lockdown-driven lifestyle and dietary changes related to COVID-19. However, as these variables were not measured in our study, this interpretation remains speculative and the effect of such associations should be systematically assessed in future studies. For instance,** extended breastfeeding throughout both years **may have also contributed**, especially if breastfeeding frequency was increased during the lockdown period, **although this cannot be determined from our data**. The lower intestinal pH in breastfed infants may inhibit the colonization of acid-sensitive bacteria, thereby shaping microbial composition, preventing pathogen growth and potentially constraining overall microbial diversity[1,73] even after solid food introduction. This effect may be more pronounced during periods of social distancing, limiting horizontal bacterial transmission. Furthermore, we cannot exclude that changes in the sample handling upon collection during this period, might have also affected the observed compositional profiles."

17. "Such resolution may uncover functionally distinct variants of Segatella that differ in their impacts on host health" - Segatella phylogenetic and functional heterogeneity is well described in literature (for instance, S. copri clade A is consistently reported as associated with biomarkers of inflammation). I suggest the authors rephrase the sentence referencing adequate literature.

Response 17: We thank the reviewer for pointing this out. We have revised the section accordingly.

Action taken:

The following sentence in the "Discussion":
"As our data revealed an association to disease in a non-Western infant cohort, future research should aim to investigate *Segatella* species with higher taxonomic resolution[84]"

has been replaced with the following:
Lines *704-736*: "**As our data revealed an association with disease in a non-Western infant cohort, future studies should prioritize higher taxonomic (i.e., species- and strain-level) resolution of *Segatella copri*, which may uncover functionally distinct variants that differ in their impacts on host health, as shown in other taxa (e.g. pathogenic and commensal *E.coli* strains[82–84]). This is critical to account for its documented phylogenetic and functional heterogeneity, as the species comprises multiple clades with distinct metabolic repertoires and divergent associations with host health, including links to inflammatory and autoimmune conditions such as Rheumatoid Arthritis, as well as to improved metabolic responses in the context of high-fiber diets (Tett et al., 2019; De Filippis et al., 2019; Scher et al., 2013; Pedersen et al., 2016).**"

18. The authors should probably comment on the small size of the cohort enrolled in the study, limiting generalizability of the findings presented.

Response 18: We appreciate the reviewer's comment and agree that this limitation should also be clearly stated in the "Discussion" section. To do so we added the following sentence:

Lines *679-686*: "Furthermore, we cannot exclude that changes in the sample handling upon collection during this period, might have also affected the observed compositional profiles. **Moreover, the relatively small cohort size of this study might limit the generalizability of our findings. However,** to date, only limited studies have examined the impact of the COVID-19 pandemic on healthy infant gut microbiota development, reporting changes in both diversity and maturation[74–76]. Further investigation into the long-term effects of the COVID-19 period is needed, particularly in LMICs, where the prevalence of other factors such as malnutrition could have compounding effects on the development of a healthy gut microbiome.

Re: mSystems00101-26R1 (Global and local trajectories of infant gut microbiota development in Bangladesh across the COVID-19 pandemic)

Dear Prof. Jelle Matthijnssens:

Thank you for the privilege of reviewing your work. Below you will find my comments, instructions from the mSystems editorial office, and the reviewer comments.

In line with journal guidelines, I have brought in a second reviewer for this round. This reviewer identified similar issues as I did before in terms of the strength of certain statements, as related to the size of the dataset and methodology used. Please restate as requested.
The reviewer also identified some areas where that were not sufficiently clear and could do with clarifications in text.

Please return the manuscript within 60 days; if you cannot complete the modification within this time period, please contact me. If you do not wish to modify the manuscript and prefer to submit it to another journal, notify me immediately so that the manuscript may be formally withdrawn from consideration by mSystems.

Revision Guidelines
To submit your modified manuscript, log into the submission site at https://msystems.msubmit.net/cgi-bin/main.plex. Go to Author Tasks and click the appropriate manuscript title to begin. The information you entered when you first submitted the paper will be displayed; update this as necessary. Note the following requirements:

• Upload point-by-point responses to the issues raised by the reviewers in a file named "Response to Reviewers," NOT in your cover letter.
• Upload a compare copy of the manuscript (without figures) as a "Marked-Up Manuscript" file.
• Upload a clean .DOC/.DOCX version of the revised manuscript and remove the previous version.
• Each figure must be uploaded as a separate, editable, high-resolution file (TIFF or EPS preferred), and any multipanel figures must be assembled into one file.
• Any supplemental material intended for posting by ASM should be uploaded with their legends separate from the main manuscript. You can combine all supplemental material into one file (preferred) or split it into a maximum of 10 files with all associated legends included.

For complete guidelines on revision requirements, see our Submission and Review Process webpage. Submission of a paper that does not conform to guidelines may delay acceptance of your manuscript.

Data availability: ASM policy requires that data be available to the public upon online posting of the article, so please verify all links to sequence records, if present, and make sure that each number retrieves the full record of the data. If a new accession number is not linked or a link is broken, provide mSystems production staff with the correct URL for the record. If the accession numbers for new data are not publicly accessible before the expected online posting of the article, publication may be delayed; please contact production staff (mSystems@asmusa.org) immediately with the expected release date.

Publication Fees: For information on publication fees and which article types are subject to charges, visit our website. If your manuscript is accepted for publication and any fees apply, you will be contacted separately about payment during the production process; please follow the instructions in that e-mail. Arrangements for payment must be made before your article is published.

ASM Membership: Corresponding authors may join or renew ASM membership to obtain discounts on publication fees. Need to upgrade your membership level? Please contact Customer Service at Service@asmusa.org.

The ASM Journals program strives for constant improvement in our submission and publication process. Please tell us how we can improve your experience by taking this quick Author Survey.

Thank you for submitting your paper to mSystems.

Sincerely,
Evelien Adriaenssens
Editor
mSystems

Reviewer #1 (Comments for the Author):

I thank the authors for the thorough explanations and replies to my comments. I think the text and methodological integrations

greatly improved the clarity of the manuscript and supported the quality of the work.

Regarding the Segatella, I'm thankful to the authors for the additional computational analysis with the existing data and I agree these are of exploratory value. I have no additional suggestions to the authors.

Reviewer #2 (Comments for the Author):

Overall comments
The authors of this study, "Global and local trajectories of infant gut microbiota development in Bangladesh across the COVID-19 pandemic", have compared the gut microbiota of a Bangladeshi cohort (BBGUT) and the BABEL cohort, which is very interesting, as each cohort appears to have distinct microbial players during microbiome maturation. Another particularly interesting aspect of this study is the observation that disease may cause a setback in microbiome maturation. This is an interesting study, and the authors have generated valuable insights. I appreciate the authors for their rigorous data analysis.

As microbiome maturation itself is a multifactorial developmental process in infants, it is influenced by numerous environmental and biological factors to which children are exposed. Therefore, I find that the authors' interpretation of the microbiome changes in the second year of life associated with COVID-19 is highly overstated. This section needs to be toned down considerably or supported with additional evidence and further analyses before such conclusions can be made. Attributing these changes primarily to COVID-19-related disturbances appears to be an overinterpretation.

Major comment:

Lines 448-492: I still find this section to be overinterpreted. Microbial maturation is primarily a developmental process. Therefore, how confident are the authors in stating that "this pattern suggests the COVID-19 lockdown measures may have interfered with the expected maturation process of the gut microbiota"? Have the authors examined whether similar patterns are present in the mothers' microbiota? The adult microbiome in the Bangladeshi cohort already appears to have relatively low Bacteroides, suggesting that this may simply represent the normal microbiome composition within this population. How confidently, therefore, can these observations be attributed specifically to COVID-19? Furthermore, how can the authors distinguish between normal developmental changes and genuine COVID-19-related maturation setbacks in these healthy children? I suggest rewriting this section to focus on describing the developmental changes observed over the second year of life in the Results, while moving the interpretation regarding the impact of COVID-19 to the Discussion. As it currently stands, presenting these conclusions within the Results section is potentially misleading.

I was unable to access the GitHub repository and, therefore, could not evaluate the code or the STORMS checklist. The authors should clearly explain how samples were randomised and distributed across sequencing runs to demonstrate that the differences observed, particularly in the maternal samples, are not sequencing artefacts.

Minor comments

Line 82: "If breastfeeding takes place, fermentative bacteria such as Bifidobacterium become dominant, facilitating the digestion of human milk oligosaccharides." Instead of "If breastfeeding takes place", this sentence could be rewritten more naturally; for example, can be started as "Breastfeeding promotes dominance of fermentative bacteria such as Bifidobacterium...".

Lines 89-91: Reference required.

Line 161 and 241: Please explain what RDP stands for.

Lines 208-209: Can the authors explain why only 20 samples were used from the Bangladeshi infants when 30 infants were sampled in the main methods?

Line 214: What do the authors mean by "early life"? Has this been defined earlier in the manuscript?

Lines 222-225: The authors state that Bifidobacterium dominates the infant gut throughout the first two years of life. However, they also state that facultative anaerobes such as Escherichia/Shigella and Streptococcus were enriched in maturation stage A. Does this imply that Bifidobacterium is less abundant or absent in stage A? This relationship should be clarified.

Lines 225-226: Please provide a reference for the statement regarding higher oxygen availability.

Line 233: Regarding the absence of Bacteroides, what do the authors mean by "nearly absent in mothers"? Can the numbers be stated here?
The absence of Bacteroides in the adult population (18 mothers) of the Bangladeshi cohort is surprising. Could this be due to sequencing or processing artefacts? Please explain how the samples were processed and sequenced. Were the mother and

infant samples processed separately, or were they distributed randomly across sequencing runs? Has this information been included in the STORMS checklist? I was unable to access the GitHub repository containing the code and checklist.

Lines 251-252: Prevotellaceae is referred to at the species level, while the following sentence immediately refers to the family Prevotellaceae. Could this be corrected?

Line 253: What do the authors mean by "several maternal faecal samples"? Please provide the exact numbers and statistics here.

Line 261: Please provide the reference for the statement regarding water retention.

Line 269: What do the authors mean by "resilience"? Please define this term in the context of this study.

Line 272: What distance metric was used for the principal coordinates analysis? I assume it was Bray-Curtis distance, but this should be explicitly stated. Will an ellipse help to see the 3 GMMs clearly here? Could the authors explain the abline 1B and the solid lines in 1E?

Line 304: What do the authors mean by key bacteria?

Lines 358-362: Interestingly, observed richness differs between 0-3 months in the BABEL cohort and the BBGUT cohort, but not during the remaining months. Also, Shannon diversity does not appear to reflect the same pattern. May I ask whether this is due to the way the age groups were binned? Have the authors examined each month separately? For example, month 4 in the BABEL cohort appears to have particularly high virome diversity, and several microbiome studies have also reported increased microbiome maturation around this age. Could the current binning strategy be masking these patterns in the Shannon diversity analysis?

Lines 373-376: Could the authors explain these results in biological terms rather than purely statistical terms? What does "cumulative explanatory power" represent biologically?

Line 379: Are these R2 values supported by p.values? If not, can the authors state it clearly?

Line 384: For the Belgian infants, could the authors provide the sample size? It would be useful to include one or two lines describing this in the Methods section.

Lines 393-398: I feel these lines on Streptococcus belong in the Discussion rather than the Results section, as there is currently no evidence supporting this claim and remains speculative.

Lines 424-428: I find this figure largely repetitive of Figure 1A, except that the mother samples have been removed. Could this be integrated?

Lines 573-579: This is a very interesting discussion. The idea that breastfeeding cessation is the primary driver of microbiota maturation in Western infants appears to contrast with the findings from the Bangladeshi cohort, where the introduction of solid foods may instead be the major driver of microbiome maturation. Could the authors discuss this apparent difference in greater detail?

Line 663: Since this is a 16S rRNA gene sequencing study, the results cannot be considered "comprehensive." The term is relative and may overstate the scope of the findings. Could the authors consider using a more appropriate term?

Lines 667-670: This statement remains too strong and should be toned down.

Dear Dr. Evelien M. Adriaenssens,
Dear Editor,

We are pleased to resubmit the second revision of our manuscript (mSystems00101-26R1) titled "*Global and local trajectories of infant gut microbiota development in Bangladesh across the COVID-19 pandemic*".

We thank the editor and both reviewers for their careful reading and for pushing us to test our own claims more rigorously. In responding to Reviewer #2 we re-examined the sequencing design and the second-year analyses, and this changed our interpretation in one important respect: we no longer attribute the second-year compositional changes to the COVID-19 lockdown, because our study design cannot separate that explanation from normal development. We are grateful for the comment that led us there. Reviewer comments are reproduced in *italics*; our responses follow each.

Editor comments

> *In line with journal guidelines, I have brought in a second reviewer for this round. This reviewer identified similar issues as I did before in terms of the strength of certain statements as they relate to the size of the dataset and methodology used. Please restate as requested. The reviewer also identified some areas that were not sufficiently clear and could do with clarifications in text.*

Response: We thank the editor for the important remark. We have restated the claims in question. In particular, the causal language linking the second-year microbiota changes to the COVID-19 lockdown has been removed from the Results, the corresponding interpretation has been moved to the Discussion and reframed as an association our design cannot test, and the concluding statements have been toned down. The clarifications requested by the reviewer have been made in the text and are itemized below.

Reviewer #1

> *I thank the authors for the thorough explanations and replies to my comments. I think the text and methodological integrations greatly improved the clarity of the manuscript and supported the quality of the work. Regarding the Segatella, I'm thankful to the authors for the additional computational analysis with the existing data and I agree these are of exploratory value. I have no additional suggestions to the authors.*

Response: We thank Reviewer #1 for the positive assessment and for the constructive comments in the previous round, which substantially improved the manuscript. The *Segatella* analyses remain presented as exploratory throughout. No further changes were required.

Reviewer #2

Overall comments

> *As microbiome maturation itself is a multifactorial developmental process in infants, it is influenced by numerous environmental and biological factors to which children are exposed. Therefore, I find that the authors' interpretation of the microbiome changes in the second year of life associated with COVID-19 is highly overstated. This section needs to be toned down considerably or supported with additional evidence and further analyses before such conclusions can be made. Attributing these changes primarily to COVID-19-related disturbances appears to be an overinterpretation.*

Response: We thank the reviewer for their insightful comment and agree with their observation. Our re-examination is described in full under the major comment below; in short, all infants in this cohort were born within a 36-day window, so infant age and calendar date are collinear by construction, and the design cannot distinguish a lockdown effect from normal development at that age. The Results now describe these changes as developmental trajectories against infant age, and the COVID-19 interpretation has been toned down, moved to the Discussion, and explicitly framed as untestable in this design.

Please note that the line numbers mentioned in each response correspond to the lines in Marked_up_Manuscript.docx.

Major comment:

<u>Comment 1:</u>

> *Lines 448-492: I still find this section to be overinterpreted. Microbial maturation is primarily a developmental process. Therefore, how confident are the authors in stating that "this pattern suggests the COVID-19 lockdown measures may have interfered with the expected maturation process of the gut microbiota"? Have the authors examined whether similar patterns are present in the mothers' microbiota? The adult microbiome in the Bangladeshi cohort already appears to have relatively low Bacteroides, suggesting that this may simply represent the normal microbiome composition within this population. How confidently, therefore, can these observations be attributed specifically to COVID-19? Furthermore, how can the authors distinguish between normal developmental changes and genuine COVID-19-related maturation setbacks in these healthy children? I suggest rewriting this section to focus on describing the developmental changes observed over the second year of life in the Results, while moving the interpretation regarding the impact of COVID-19 to the Discussion. As it currently stands, presenting these conclusions within the Results section is potentially misleading.*
>
> *I was unable to access the GitHub repository and, therefore, could not evaluate the code or the STORMS checklist. The authors should clearly explain how samples were randomised and*

distributed across sequencing runs to demonstrate that the differences observed, particularly in the maternal samples, are not sequencing artefacts.

Response 1: We thank the reviewer for these comments, which prompted a re-examination of our interpretation. We answer the six points in turn.

(i) We no longer stand by suggesting the impact of COVID-19 lockdown in our results. We had not given enough weight to a feature of our cohort: all 20 infants were born within a 36-day window (26 October – 1 December 2018), so the onset of the national lockdown in Bangladesh (26 March 2020) falls at infant age 15.8–17.0 months for every child in the cohort. Infant age and calendar time are therefore collinear by construction, and no infant contributes samples that could enable testing the impact of lockdown. The distinction the reviewer asks us to draw is not possible in this design, and it is now explicit in the text to avoid any assumptions in this regard.

(ii) The mothers cannot serve as a comparator, but they do establish the population baseline, and the reviewer's inference from them is correct. As each mother contributed a single fecal sample collected shortly after birth in late 2018, with no longitudinal follow-up, the maternal samples cannot be used as a control to test for a lockdown effect. However, they do establish the population baseline, and it is as the reviewer suggests: in this cohort *Bacteroides* is essentially absent from the mothers (mean 0.07%, median 0.00%; detected at ≥ 0.5% in 1 of 18), while *Prevotellaceae* dominate (mean 27.2%, median 22.1%), with *Segatella* detected in 15 of 18 mothers and contributing 21.9% on average. This fits the reported adult configuration for Bangladeshi[1]and other South Asian populations[2], and we now present low *Bacteroides* as the local adult norm rather than as an isolated oddity.

(iii) Results and Discussion have been restructured as suggested. We deleted the sentence "*this pattern suggests the COVID-19 lockdown measures may have interfered with the expected maturation progress of the gut microbiota*", together with "*As previously discussed, this decline coincided with the COVID-19 lockdown measures in Bangladesh*." The Results (Lines 511–543) now describe these changes as developmental trajectories against infant age without linking it to the lockdown; the pandemic appears only in the Discussion (Lines 764–774) as a coincidence this study cannot test; and the Conclusion has been toned down (Lines 818–822, see Comment 24). We also note in the Discussion that sample handling changed during this period (home −20 °C storage, metadata collected by telephone), a further non-biological variable that is equally inseparable from infant age.

(iv) Repository access. We apologize for the access problem, which has been corrected. The repository at https://github.com/Matthijnssenslab/Bangladesh_InfGut_16S is public and contains the analysis code and the completed STORMS checklist. The raw sequence data are deposited under accession number PRJEB95966 at the European Nucleotide Archive (ENA).

(v) Randomization across sequencing runs, and the maternal result. Samples were randomized throughout laboratory processing from aliquoting onward and sequenced across seven Illumina MiSeq runs (DI22R01–DI22R06, DI22R28). Each run carried samples from 17–20 of the 20 infants. We disclose two departures from full randomization: all 18 maternal samples were sequenced on one run (DI22R28, together with 84 infant samples analyzed in this study), and because year-1 and year-2 material was shipped in two batches (December 2019, January 2021), run DI22R06 carries 154 of the 175 analyzed year-2 samples (88%), so study year is largely confounded with run. This is now described in the Supplementary Extended Methods and in STORMS item 4.13.

Because that confound also makes run a proxy for infant age, we partitioned the two terms rather than comparing them: run accounts for 57% of the variance in infant age (one-way ANOVA $\eta^2 = 0.570$, $p < 2 \times 10^{-16}$). Fitted alone, run explains $R^2 = 0.072$ of infant community variation; fitted after age, $R^2 = 0.019$ remains, so most of its apparent effect is variance shared with age. Because age enters as a single term while run receives six degrees of freedom, this understates age; with the two given equal degrees of freedom, age explains $R^2 = 0.151$ against run's $R^2 = 0.011$, and the age signal is significant within each of the seven runs analyzed separately. A small, genuine run effect does remain among the six age-mixed runs once the run/year confound is removed ($R^2 = 0.014$ beyond age), and we describe it as small rather than absent. Details are also provided in STORMS item 7.7.

The second year shift itself is likewise not a between-run artefact. Because run DI22R06 carries 154 of the 175 analyzed year-2 samples, the months 13–16 versus 17–24 contrast is made almost entirely within a single sequencing run, so differences between runs cannot account for it. We state this as the narrower claim it is: the contrast is internal to one run, rather than replicated across a partition of runs.

(vi) The maternal result is unlikely to be a sequencing artefact. More broadly, the quality-control data indicate that the microbiota profiles generated in this study predominantly reflect biological variation rather than technical variability. Each sequencing run included a positive control consisting of identical fecal DNA sequenced across all seven runs, allowing direct assessment of run-to-run variation. The positive control recovered *Bacteroides* consistently, including on DI22R28 containing the maternal samples (8.3% versus a mean of 8.6% and a range of 7.7–10.6% across runs), demonstrating consistent recovery of the genus from identical input material.

Technical variation between runs was substantially lower than biological variation among study samples (median Bray–Curtis dissimilarity: 0.079 among positive controls versus 0.549 among study samples), and sequencing run did not significantly explain positive-control composition (PERMANOVA, $R^2 = 0.421$, $p = 0.662$) (Supplementary Fig. S3). In addition, ZymoBIOMICS mock community samples, processed through the complete workflow including DNA extraction, consistently recovered its eight constituent genera across runs (median Pearson correlation = 0.94;

median Bray–Curtis distance = 0.10, 99.30–100.00% on-target; DI22R28: 99.30%) and contained no *Bacteroides* in any of its 13 libraries, excluding contamination as a source of the genus. Index cross-talk was also negligible, as shown by the *Runella slithyformis* control (99.96–99.99% on target).

Within this validated framework, the maternal profile: low *Bacteroides* abundance and dominance of *Prevotellaceae* is consistent with previously reported Bangladeshi[1] and other South Asian populations[2]. Together, these results indicate that the observed microbiota patterns, including the maternal profile, primarily represent biological signals rather than technical variability or sequencing noise.

[1]Lin, A. *et al.* Distinct distal gut microbiome diversity and composition in healthy children from Bangladesh and the United States. *PLoS One* 8, e53838 (2013).
[2]Ramachandran, S. L. et al. Distinct trajectories of urbanization shape the human gut microbiome across South Asia. *bioRxiv* (preprint) 2026.01.22.701183 (2026).

Actions taken:

To address the reviewer's major comment, the following sections of the manuscript were modified (changes highlighted in bold):

Importance:

Lines *62-64*: In this study, we longitudinally characterized the gut microbiota development in a healthy infant cohort from urban Bangladesh, showing that geographically distinct populations can harbor different microbial communities while following similar developmental patterns. **We further observe that illness can temporarily influence microbiota maturation, and that part of the microbiota maturation period in this cohort overlapped with a major societal disruption, the COVID-19 pandemic.** Together, these findings illustrate the importance of geographic, cultural, and environmental context when defining healthy gut microbiota trajectories and contribute to a more inclusive, globally representative framework for understanding early-life microbiota development.

Introduction:

Lines *115-119*: In this study, we analyzed 16S rRNA data from a densely sampled longitudinal cohort of healthy Bangladeshi children (BBGUT cohort) to investigate early-life gut microbiota dynamics. Specifically, we characterized the development of the gut bacterial community from birth to two years of age in an urban infant population from Dhaka, **a period that partially overlapped with the COVID-19 pandemic and** explored factors that may influence this process.

Results:

Lines *511-533*: While analyzing the monthly progression of gut microbiota maturation in the BBGUT cohort (considering only HTP samples) we noticed a qualitative decrease in the relative abundance of some taxa during the second year (Fig. 4a). **To further assess these shifts, we compared period-specific changes in bacterial relative abundances and identified several genera with significantly greater fluctuations after 16 months of age (Supplementary Fig. S13, Supplementary Table S9). We therefore divided the second year into two periods, months 13–16 and months 17–24 of infant age. As all infants were born within a 36-day window, this boundary also coincides with the onset of the national COVID-19 lockdown in Bangladesh (26 March 2020; Supplementary Fig. S14), meaning that infant age and calendar period cannot be distinguished in this cohort.** For this analysis we focused on samples representing healthy time points due to a limited number of samples associated with disease in year 2 (n=17/175 samples). Comparing the two periods revealed a statistically significant decline in the relative abundance of several common bacteria, including *Segatella, Bacteroides, Veillonella* and *Megasphaera*, observed during **months 17-24** (Fig. 4b, Supplementary Table S10). Only *Blautia*, a genus suggested to have probiotic potential[54] and *Streptococcus*, showed an increase in relative abundance during this time. **Although changes in these taxa were observed, the current study design does not allow us to determine whether they were driven by pandemic-related factors.**

Lines *534-543*: Next, we compared maturation scores between infants before and **after 16 months of age** (calculated based on HTP samples), accounting for bacterial rankings and presence across monthly samples throughout their second year of life as previously discussed (Fig 3b). Although there was no significant overall decrease in the maturation score during **months 17-24** (Fig. 4c), and alpha diversity indices similarly showed no significant change between the **two age periods** (linear mixed model, p=0.44 [Shannon index], p=0.43 [observed richness]; Supplementary Table S3), individual responses varied, with some infants showing signs of plateaued or reduced maturation. **While** these results do not support a consistent, cohort-wide delay in microbiota maturation (Fig. 4b), we identified temporal perturbations during this period, as reflected by changes in the relative abundance of specific bacterial taxa (Fig. 4c).

In addition to the above, **Figure 4** (manuscript) and **Supplementary Figure S13** (Supplementary_Figures_withlegends.docx) have been updated:

Lines *545-556*: **Fig 4. (a)** Mean relative abundance of the most common bacterial genera found in the Bangladeshi infant cohort during the first two years of life (0-24 months). To account for the rapid changes in infant gut composition after birth, the first 10 days of life have been grouped as Month 0. The number of healthy samples assigned to each month is shown at the top of each bar, and the period that coincides with the onset of the first COVID-19 lockdown measures in

Bangladesh is marked on the x-axis. **(b)** Bacterial genera with a statistically significant difference in their median relative abundance when comparing **the 13-16 month** and **17-24 month** groups during the second year of life (Wilcoxon signed-rank test, p<0.05, FDR<0.05). **(c)** Average weighted maturation score comparison between the **two age** periods during the second year of life. Each point represents an infant's maturation score in each period calculated considering HTP samples only, with colors distinguishing different children. Lines connect scores for the same infant across the two periods, illustrating individual trajectories in maturation patterns. (Wilcoxon signed-rank test, p=0.46). Note that data for the second year include 16 out of 20 infants.

Supplementary figure S13. Boxplots showing two-month fluctuations in genus-level bacterial abundances **across early (months 13–16) and late (months 17–24) age windows**. Normalized median abundance changes at the genus level were calculated using all valid pairs of equidistant time points spaced two months apart, for each child and bacterial genus, across the 9–24 month age range. For each child and genus, the normalized change was defined as the difference between the two median abundances divided by their average. Sample pairs were categorized into three period groups: ***E–E*** (both time points during the **early age window** period, months 9–16), ***E–L*** (crossing from **early to late age window**, months 15–18), and ***L–L*** (both time points during the **late age window** period, months 17–24). Statistical differences between groups were assessed using linear mixed-effects models fitted separately for each genus, with fixed effects for period group and age, and random intercepts for each child. Genera with significant differences between period comparisons ($p < 0.05$) are highlighted in the figure.

Discussion:

Lines *724-736*: Several studies have shown that neonatal antibiotic exposure can delay microbiota maturation, reduce bifidobacteria abundance, increase the abundance of *Enterobacteriaceae* species, and lower overall microbial diversity[10,66–69]. In LMICs, early-life exposure to a broad range of enteric pathogens, often subclinical infections, is common and may influence the typical colonization process[38]. In this cohort, we report specific setbacks in microbiota maturation coinciding with antibiotic use and events of concurrent antibiotic administration and gastrointestinal disease. Despite these acute setbacks, no major persistent impacts on gut microbiota development were observed during early life. **Maturation-stage regressions were also observed in healthy samples during the second year of life (Fig. 1e). In this study, this interval overlapped almost entirely with the lockdown period in Bangladesh, suggesting that these regressions cannot be attributed specifically to lockdown-related factors. Instead, the illness episodes and antibiotic exposures recorded during this period represent potential contributing factors more directly supported by the available data.**

Lines *744-747*: Sampling in the BBGUT cohort partially overlapped with the early COVID-19 lockdown measures in Bangladesh, resulting in most of the second year being spent under

restrictions. **To our knowledge, this is the first longitudinal study of healthy infant gut microbiota development in Bangladesh to span the pandemic period within a single cohort.**

Lines *764-774*: **However, because all infants experienced the first COVID-19 lockdown at the same developmental stage, the effects of pandemic-related restrictions cannot be separated from normal age-related microbiota development in this study.** Furthermore, we cannot exclude that changes in the sample handling upon collection during this period, might have also affected the observed compositional profiles. **Finally,** the relatively small cohort size of this study might limit the generalizability of our findings.

Another modification in Discussion is also addressed in **Comment 24**. Supplementary Tables S9, S10, have also been updated accordingly.

In addition, the following lines were added in Supplementary Extended Methods:

Lines *86-97*: **To explore the gut microbiota development during the second year of life, we divided the data into two calendar-based periods according to the recorded sample collection date. Bangladesh declared a nationwide general holiday, with movement restrictions, from 26 March 2020. Samples collected before and on or after that date were classified in two age periods: the months 13-16 period, corresponding to samples collected before the lockdown was declared and the months 17-24 period, corresponding to samples collected after this date. All samples fell within their respective age-period classifications, with the exception of one (I01_151), which, although collected at month 17, was obtained before the lockdown was declared and was therefore included in the months 13–16 period. The comparison was restricted to healthy (HTP) infant samples from the second year of life, giving 52 samples in the months 13-16 period (16 November 2019 – 25 March 2020) and 106 in the months 17-24 period (31 March – 19 November 2020, infant age 17–24 months) from 16 infants.**

Lines *114-143*: All samples were randomized at every stage of laboratory processing, **beginning with aliquoting and continuing through the seven Illumina MiSeq sequencing runs, each of which comprised two 96-well plates. Each run included samples from 17–20 of the 20 infants. Two departures from complete randomization occurred: all 18 maternal samples were sequenced in a single run, and because year-1 and year-2 samples were shipped in two batches, most year-2 samples (154/175 analyzed samples; 88%) were sequenced in one run, resulting in confounding between study year and sequencing run (described in more detail under STORMS item 7.7).** Negative controls (DNA extraction negative control and PCR negative control) included along the whole process. Positive controls, specifically: DNA from a fecal sample analyzed previously, a *Runella slithyformis* control and a commercial mock community (ZymoBIOMICS) were also used **to assess technical variability across the entire workflow**.

To evaluate whether the maternal *Bacteroides* abundance results could be explained by sequencing batch effects, we analyzed the control series included on every plate of each MiSeq run. The positive control, comprising identical fecal DNA sequenced across all seven runs, showed consistent *Bacteroides* recovery (mean 8.6%, range 7.7–10.6%), including in the run containing the maternal samples (8.3%), indicating normal recovery from identical input DNA. Technical variation among positive-control libraries was substantially lower than biological variation among study samples (median Bray–Curtis dissimilarity 0.079 versus 0.549), and sequencing run did not significantly influence positive-control composition (PERMANOVA, $R^2 = 0.42$, $p = 0.66$) (Supplementary Fig. S3). In addition, the *Runella slithyformis* control was 99.96–99.99% on target across runs, and negative controls contained only ~0.5% of the median study-sample read depth. Mock communities showed consistent genus-level taxonomic profiles across sequencing runs (median Pearson correlation = 0.94; median Bray–Curtis dissimilarity = 0.10, 99.30–100.00% on-target), supporting the reproducibility of the sequencing and analysis workflow. Together, these results indicate that the observed microbiota patterns, including the maternal profile, primarily represent biological signals rather than technical variability or sequencing noise.

New figure: Supplementary Fig. S3

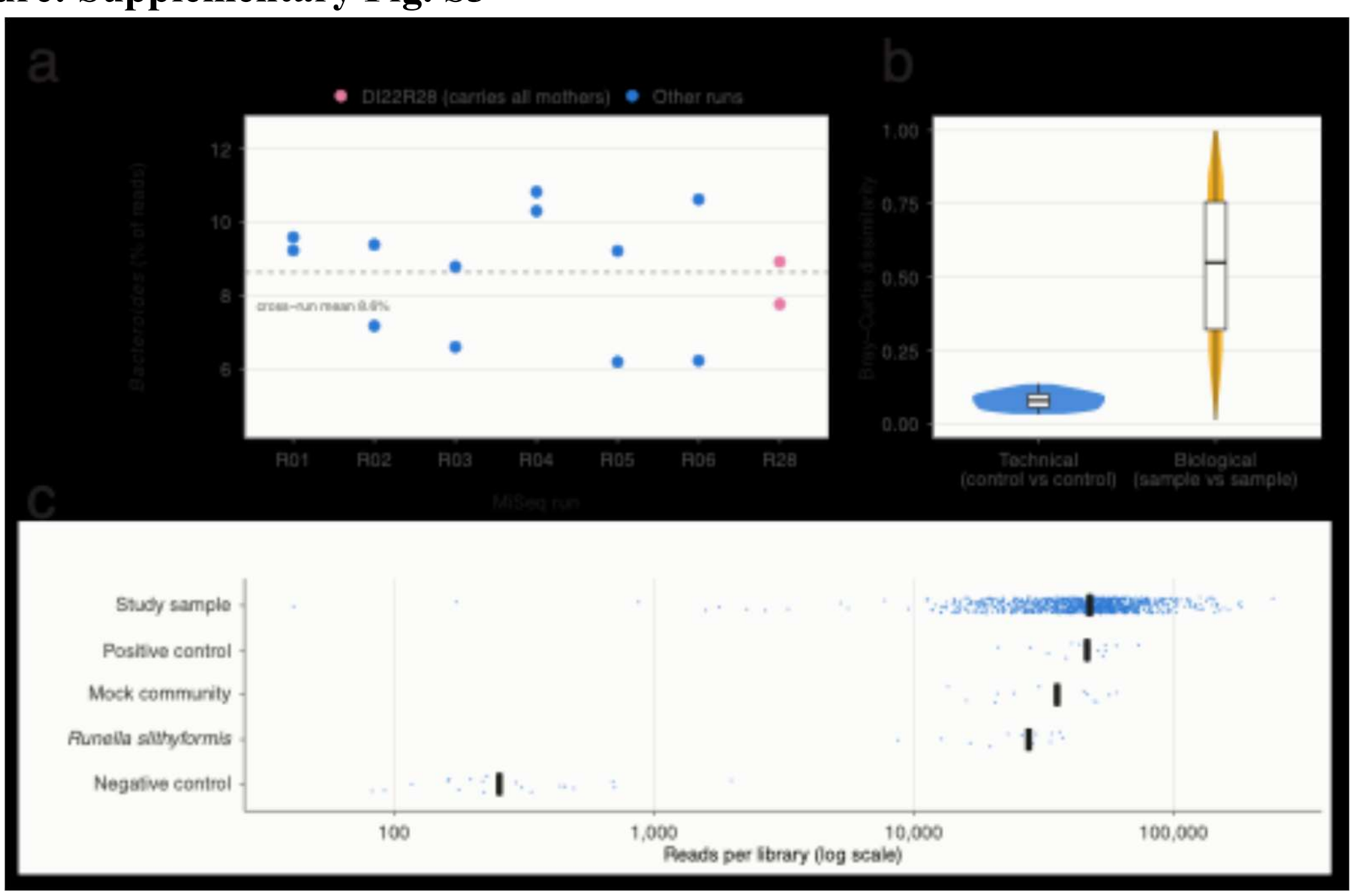

Supplementary Fig. S3. Control-based quality control across the seven MiSeq runs shows that the maternal *Bacteroides* result is not a sequencing artefact. A control series was included on every 96-well plate of every run, allowing run-to-run technical variation to be measured directly rather than inferred. Run labels are abbreviated (R01 = DI22R01 … R28 = DI22R28). Taxonomy was re-assigned from the raw ASV tables against RDP 19; plant, host and non-prokaryotic sequences were removed before normalization, and libraries were rarefied to even depth within each comparison.

(a) Bacteroides relative abundance in the positive control, an identical fecal DNA extract sequenced on all seven runs (2 libraries per run; rarefied to 21,149 reads). Each point is one library; the dashed line is the cross-run mean (8.6%). Recovery is consistent across runs (range 7.7–10.6%). Run DI22R28, which carries all 18 maternal samples (pink), gives 8.3%, i.e. $z = -0.30$ relative to the other six runs, and its library on plate p18, the plate carrying the maternal samples, gives 7.8%. The run and plate that carry the mothers therefore recover *Bacteroides* normally from identical input DNA. Sequencing run did not significantly explain positive-control composition (PERMANOVA on Bray–Curtis dissimilarities, $R^2 = 0.42$, $p = 0.66$, $n = 14$ libraries). Values are for *Bacteroides* sensu stricto as circumscribed by RDP 19, which places several former *Bacteroides* species in *Phocaeicola.*

(b) Bray–Curtis dissimilarity between positive-control libraries (technical variation, identical input DNA) and between 200 randomly selected study samples (biological variation). Violins show the full distribution; boxes show the median and interquartile range. Technical variation (median 0.079) is ~6.9-fold smaller than biological variation (median 0.549).

(c) Reads per library by class (log scale; bar = median). ZymoBIOMICS mock communities are also shown (n=13, median 35,586 reads). Negative controls (DNA-extraction and PCR blanks) carry a median of 253 reads, ~0.5% of the median study-sample depth (47,537 reads), indicating low background. The single-organism *Runella slithyformis* control was 99.96–99.99% on target across runs (off-target 0.006–0.040%), indicating negligible index hopping or cross-talk between libraries.

Minor comments

Comment 2:

> *Line 82: "If breastfeeding takes place, fermentative bacteria such as Bifidobacterium become dominant, facilitating the digestion of human milk oligosaccharides." Instead of "If breastfeeding takes place", this sentence could be rewritten more naturally; for example, can be started as "Breastfeeding promotes dominance of fermentative bacteria such as Bifidobacterium...".*

Response 2: We thank the reviewer for this suggestion.

Actions taken: The sentence has been revised as follows:

Lines *87-88*: Breastfeeding promotes dominance of fermentative bacteria such as *Bifidobacterium*, facilitating the digestion of human milk oligosaccharides (HMOs)[11].

Comment 3:

> *Lines 89-91: Reference required.*

Response 3: We thank the reviewer for pointing this out.

Actions taken: The following references have been added to support the sentence below:

Lines *94-96*: Geographical and cultural differences also shape microbial composition. Studies have consistently shown that gut microbiota diversity and structure vary with urbanization, westernization, and lifestyle[9,15–17].

- [9]Derrien, M., Alvarez, A.-S. & de Vos, W. M. The gut Microbiota in the first decade of life. Trends Microbiol. 27, 997–1010 (2019).
- [15]Obregon-Tito, A., Tito, R. Y., Metcalf, J. L. *et al.* Subsistence strategies in traditional societies distinguish gut microbiomes. Nat. Commun. 6, 6505 (2015).
- [16]Lin, A. et al. Distinct distal gut microbiome diversity and composition in healthy children from Bangladesh and the United States. PLoS One 8, e53838 (2013).
- [17]de Goffau, M. C. et al. Gut microbiomes from Gambian infants reveal the development of a non-industrialized Prevotella-based trophic network. Nat. Microbiol. 7, 132–144 (2022).

Comment 4:

Line 161 and 241: Please explain what RDP stands for.

Response 4: We thank the reviewer for pointing this out. We have revised the manuscript to define RDP at its first occurrence as the Ribosomal Database Project (RDP) and to improve clarity for readers.

Actions taken: The full name of the RDP database has now been clarified below:

Lines *173-175*: Taxonomy was assigned using the formatted RDP **(Ribosomal Database Project)** training set "rdp_train_set_19"[31] after merging paired sequences and eliminating chimeras.

Comment 5:

Lines 208-209: Can the authors explain why only 20 samples were used from the Bangladeshi infants when 30 infants were sampled in the main methods?

Response 5: We appreciate the reviewer's suggestion and provided an additional explanation in the Materials and Methods section of the main manuscript.

Actions taken: The following sentence (highlighted in bold) was added in the manuscript's "Study design and sample selection" section:

Lines *144-146*: **Due to incomplete sampling and missing metadata, only 20 of the 30 enrolled infants were included in this study.** In total, 1,002 samples were selected for this study, from which 18 were maternal and 984 originated from 20 infants.

Comment 6:

> *Line 214: What do the authors mean by "early life"? Has this been defined earlier in the manuscript?*

Response 6: We appreciate the reviewer's remark.

Actions taken: We have modified the following sentence to improve clarity:

Lines *228-231*: Specifically, **during the first months of life**, infant samples predominantly clustered within stage A, gradually transitioning to stage B, and ultimately reaching stage C around the beginning of the second year of life (Fig. 1b, Supplementary Fig. S6a).

Comment 7:

> *Lines 222-225: The authors state that Bifidobacterium dominates the infant gut throughout the first two years of life. However, they also state that facultative anaerobes such as Escherichia/Shigella and Streptococcus were enriched in maturation stage A. Does this imply that Bifidobacterium is less abundant or absent in stage A? This relationship should be clarified.*

Response 7: We thank the reviewer for raising this point. We agree that the original wording did not clearly convey the message.

Actions taken: We have revised the following lines (changes highlighted in bold):

Lines *237-240*: *Bifidobacterium* dominated the Bangladeshi infant gut throughout the first two years of life. Facultative anaerobes such as *Escherichia/Shigella* and *Streptococcus* **were also highly abundant** in maturation stage A, **but unlike *Bifidobacterium*,** these taxa declined in stages B and C (Fig. 1c).

Comment 8:

Lines 225-226: Please provide a reference for the statement regarding higher oxygen availability.

Response 8: We appreciate the reviewer's remark.

Actions taken: The missing reference has been added to support the relevant statement:

Lines *240-242*: A similar trend was observed in Belgian infants[10], suggesting that these genera may be indicative of a conserved early-life gut environment, with higher oxygen availability[37].

[37]Espey, M. G. Role of oxygen gradients in shaping redox relationships between the human intestine and its microbiota. *Free Radic. Biol. Med.* 55, 130–140 (2013).

Please note that in the revised manuscript version the numbering of the references has been updated.

Comment 9:

Line 233: Regarding the absence of Bacteroides, what do the authors mean by "nearly absent in mothers"? Can the numbers be stated here?
The absence of Bacteroides in the adult population (18 mothers) of the Bangladeshi cohort is surprising. Could this be due to sequencing or processing artefacts? Please explain how the samples were processed and sequenced. Were the mother and infant samples processed separately, or were they distributed randomly across sequencing runs? Has this information been included in the STORMS checklist? I was unable to access the GitHub repository containing the code and checklist.

Response 9: We thank the reviewer for this valuable comment. We have clarified the meaning of "nearly absent" by providing additional information as requested. Across the 18 maternal samples, 4 individuals had *Bacteroides* abundances below the 0.5% relative abundance threshold, while only one individual had an abundance value marginally above this cutoff.

Actions taken: We have added this clarification to the revised manuscript:

Lines *249-251*: Notably, *Bacteroides* had a relatively low abundance in Bangladeshi infants and was nearly absent in their mothers (**1/18 individuals with a relative *Bacteroides* abundance of >0.5%**; Fig. 1c, Supplementary Fig. S7), a key difference from Western populations.

We appreciate the reviewer's concern regarding the possibility that these observations could be related to sequencing or processing artefacts. We have addressed these points in detail in our response to the major comment by providing information on the sequencing design, the sample distribution across runs, while we performed additional control-based quality control analyses to test for batch effects (**new Supplementary Fig. S3**, see response 1 to major comment).

Importantly, the control-based quality control assessment across the different MiSeq runs indicates that the observed *Bacteroides* result is not attributable to a sequencing artefact. We hope these additional analyses and clarifications adequately address the reviewer's concerns.

Comment 10:

> *Lines 251-252: Prevotellaceae is referred to at the species level, while the following sentence immediately refers to the family Prevotellaceae. Could this be corrected?*

Response 10: We thank the reviewer for this suggestion.

Actions taken: We have corrected the phrasing accordingly:

Lines *274-275*: …. **where species of the** *Prevotellaceae* **family** dominate the gut microbiome[13,16,17,19,24].

Comment 11:

> *Line 253: What do the authors mean by "several maternal faecal samples"? Please provide the exact numbers and statistics here.*

Response 11: We appreciate this remark and have revised the sentence to include quantitative information, providing a more precise description.

Actions taken: The sentence below:
"Compared to infants, members of the *Prevotellaceae* family were enriched in several maternal fecal samples (Supplementary Fig. S7)."

Was modified as follows (changes highlighted in bold):

Lines *275-278*: Compared to infants, members of the *Prevotellaceae* family exhibited high relative abundance in several maternal fecal samples **(15/18 individuals harbored *Segatella* alone or in combination with *Leyella* and/or *Prevotellamassilia*; mean combined relative abundance: 30.5%)** (Supplementary Fig. S7).

Comment 12:

> *Line 261: Please provide the reference for the statement regarding water retention.*

Response 12: We thank the reviewer for noting this.

<u>Actions taken:</u> We have adjusted the citations to the appropriate position within the sentence to make its association with the statement clearer.

Lines *285-286*: Consumption of non-fermentable fiber increases stool water content and plasticity, contributing to increased water retention in the gut[49,52].

- [49]Vandeputte, D., Falony, G., Vieira-Silva, S., Tito, R. Y., Joossens, M. & Raes, J. Stool consistency is strongly associated with gut microbiota richness and composition, enterotypes and bacterial growth rates. *Gut* 65, 57–62 (2016).
- [52]Mudgil, D. & Barak, S. Composition, properties and health benefits of indigestible carbohydrate polymers as dietary fiber: A review. *Int. J. Biol. Macromol.* 61, 1–6 (2013).

<u>Comment 13:</u>

> *Line 269: What do the authors mean by "resilience"? Please define this term in the context of this study.*

Response 13: We appreciate this comment and have rephrased the sentence to more accurately describe "resilience" in the context of this study which is the maintenance of a structured and predictable maturation trajectory despite occasional regressions.

<u>Actions taken:</u> The following modification was made (in bold):

Lines *291-296*: Despite occasional regressions to earlier maturation stages (Fig. 1e) - potentially due to illness, medication, or other factors - we observed a conserved and structured trajectory of microbiota maturation over time in 3 distinct GMM stages across infants (Fig. 1e, $n = 20$, Kendall test, Kendall's corrected $w = 0.91$, and $p = 9.9e{-5}$). This **finding** highlights the resilience of the Bangladeshi infant gut microbiome **in** its ability **to maintain a structured and predictable developmental pattern despite occasional regressions.**

<u>Comment 14:</u>

> *Line 272: What distance metric was used for the principal coordinates analysis? I assume it was Bray-Curtis distance, but this should be explicitly stated. Will an ellipse help to see the 3 GMMs clearly here? Could the authors explain the abline 1B and the solid lines in 1E?*

Response 14: We thank the reviewer for these helpful suggestions. Indeed, the distance metric used for the PCoA was Bray–Curtis dissimilarity, which is now explicitly stated in the caption of Figure 1 in the revised manuscript.
Regarding the ellipses in the PCoA plot, we evaluated whether they would improve visualization of the three GMMs. However, because the clusters partially overlap, the ellipses did not improve the interpretability of the figure (Figure R2). We therefore retained the initial figure.
The abline in manuscript Figure 1b indicates the separation between the two sampling years, and the solid lines in Figure 1e connect longitudinal samples from the same individual to highlight individual microbiome maturation trajectories. Both are used to aid visualization. These descriptions have now been included in the caption of Figure 1.

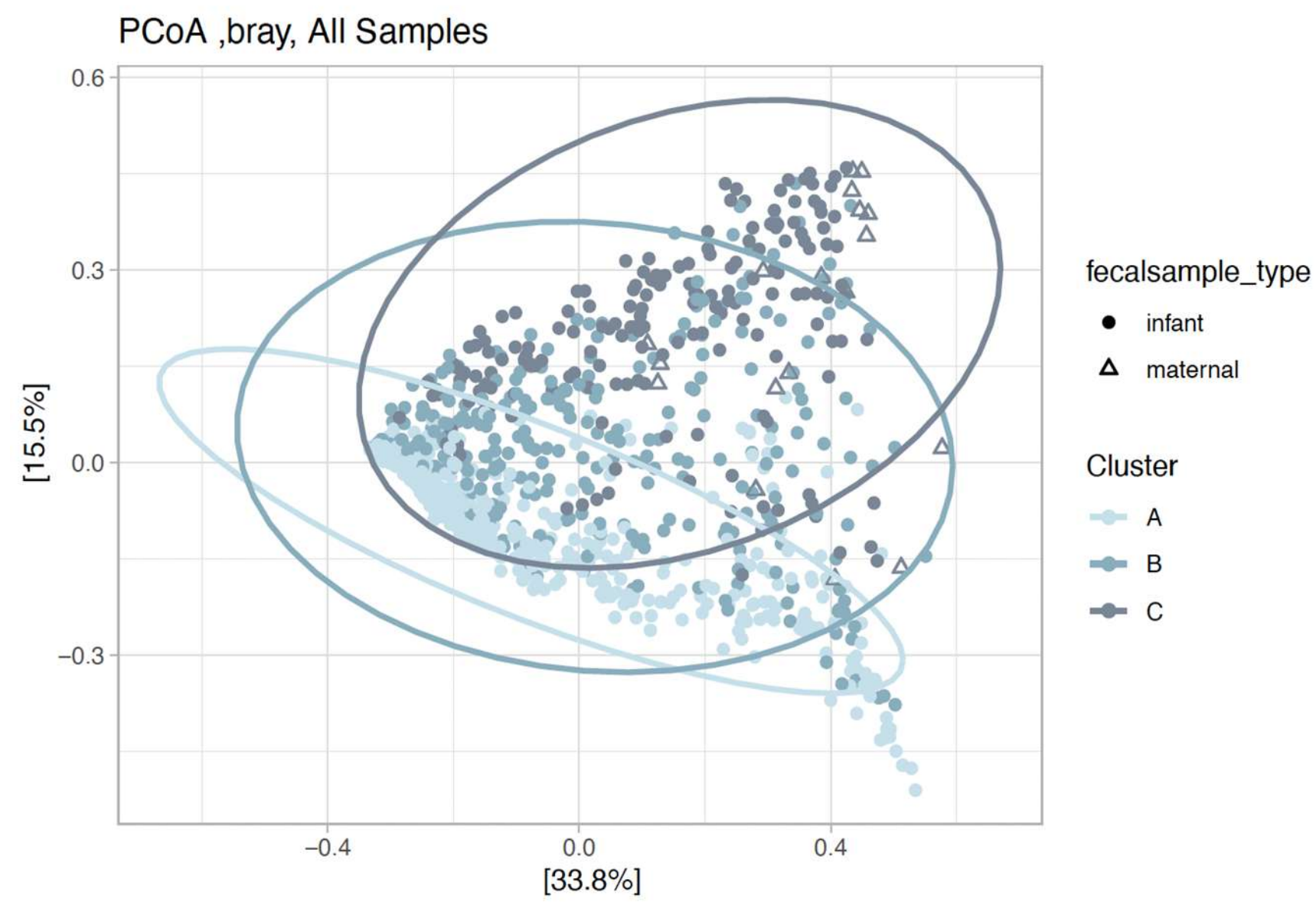

Figure R2: Reconstruction of the PCoA in Figure 1a of the original manuscript including ellipses.

Actions taken: The caption of Figure 1 in the manuscript was modified as follows (in bold):

Lines *298-324*: Fig 1. (a) Principal coordinate analysis (PCoA) **based on Bray–Curtis dissimilarity** showing the clustering of all infant (n = 984) and maternal samples (n = 18). Samples are colored according to gut microbiota maturation stage, with circles representing infant samples and triangles representing maternal samples. (b) Density plot illustrating the distribution of sample age (in days) across GMM stages A, B, and C. **The vertical dashed line indicates the separation between year 1 and year 2 at day 365.** …. (e) Stepwise progression of gut microbiota maturation stages per infant. Identified maturation stages, determined using the DMM analysis at the genus level on the combined infant and maternal samples (n=1002), are represented in three distinct colors along the vertical axis. Here, only HTP infant samples (n = 486) are shown. The horizontal axis represents age in days (0–2 years), with dashed lines indicating the separation between the first and second year of life. **Solid lines are used to connect the samples of each individual for visualization purposes.** Only 16/20 selected infants have samples spanning both years.

<u>Comment 15:</u>

Line 304: What do the authors mean by key bacteria?

Response 15: We thank the reviewer for this remark. We agree that the term "key bacteria" was insufficiently specific. We used this term to refer to the most abundant bacterial genera that were present in both cohorts.

<u>Actions taken:</u> The following sentence has been revised to improve clarity:

Lines *338-340*: While **some of these** bacteria followed similar patterns in acquisition and persistence in both cohorts during early life, the relative abundance dynamics differed over time for most genera (Supplementary Fig. S8a, b).

<u>Comment 16:</u>

Lines 358-362: Interestingly, observed richness differs between 0-3 months in the BABEL cohort and the BBGUT cohort, but not during the remaining months. Also, Shannon diversity does not appear to reflect the same pattern. May I ask whether this is due to the way the age groups were binned? Have the authors examined each month separately? For example, month 4 in the BABEL cohort appears to have particularly high virome diversity, and several microbiome studies have also reported increased microbiome maturation around this age. Could the current binning strategy be masking these patterns in the Shannon diversity analysis?

Response 16: We thank the reviewer for raising this important point. To address this comment, we repeated the alpha diversity analysis using individual months of age rather than age bins (Fig. R3).

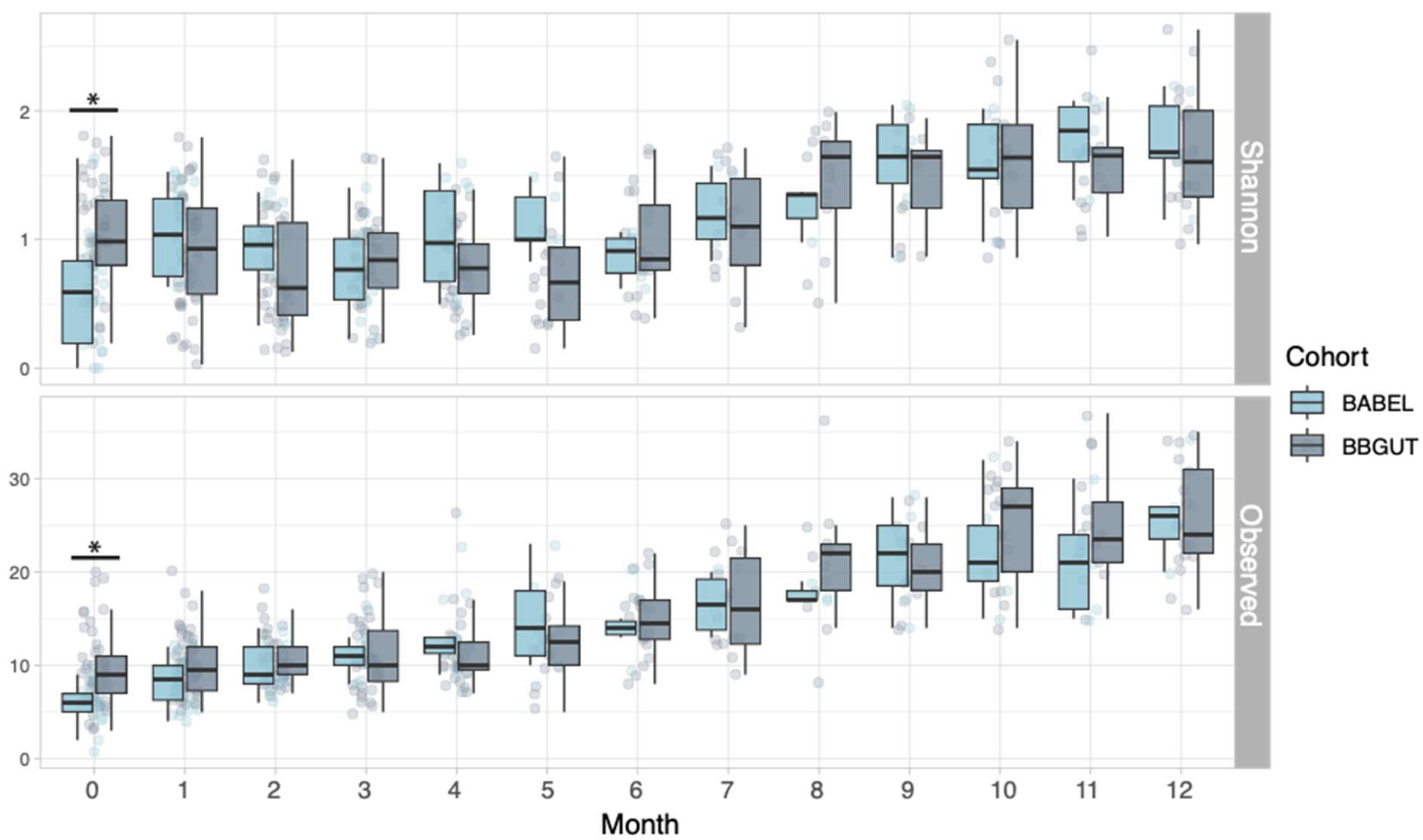

Figure R3. Month-specific analysis of alpha diversity (observed and Shannon diversity) measures between cohorts. Samples are shown as individual points with boxplots representing the distribution within each cohort. Statistical comparisons between cohorts were performed for each month using Wilcoxon rank-sum tests, with p-values adjusted for multiple testing using the Benjamini–Hochberg method. Significant differences were observed only at month 0 (first 10 days of life) for observed richness (adjusted $p = 0.003$) and Shannon diversity (adjusted $p = 0.005$). No significant differences were detected between cohorts at any subsequent month (adjusted $p > 0.05$).

This analysis showed that the only statistically significant difference between cohorts occurred at month 0 (representing the first 10 days of life) for both observed richness and Shannon diversity. For Shannon diversity only, this difference was not detected in the original 0–3 month age bin comparison, likely because the immediate postnatal period represents a short and highly dynamic window that was combined with subsequent months in the broader age bin in Fig. 2c of the manuscript. However, no significant differences between the two cohorts were detected at any subsequent month, including month 1 and month 4, after correction for multiple testing (adjusted $p > 0.05$).

Since this analysis did not change the overall interpretation of the alpha diversity results, we have retained Fig. 2c in the manuscript. We have instead included Figure R3 as Supplementary figure S9 and expanded on the Results section.

<u>*Actions taken:*</u> The following information was added in Results:

Lines *361-366*: **To determine whether these observations resulted from age-binning effects, we performed a month-by-month analysis of alpha diversity (Supplementary Fig. S9).**

Differences in alpha diversity were statistically significant only during the first 10 days of life (Shannon diversity and observed richness, Wilcoxon rank-sum tests, adjusted p<0.01) while subsequent time points showed similar patterns, supporting our initial conclusion that alpha diversity follows a consistent trajectory over time.

Comment 17:

Lines 373-376: Could the authors explain these results in biological terms rather than purely statistical terms? What does "cumulative explanatory power" represent biologically?

Response 17: We thank the reviewer for this suggestion and have modified the phrasing to better explain the results in biological terms.

Actions taken: The following sentence was modified:
"Overall, the cumulative explanatory power of the non-redundant variables in our multivariate distance-based redundancy analysis (dbRDA) accounted for 24% of the variance in our data (Fig. 3a)."

And now reads as follows:

Lines *424-427*: Overall, the non-redundant variables in our multivariate distance-based redundancy analysis (dbRDA) **collectively explained approximately** 24% **of the variation in the microbial community composition** (Fig. 3a, Supplementary Table S6).

Comment 18:

Line 379: Are these R2 values supported by p.values? If not, can the authors state it clearly?

Response 18: We thank the reviewer for raising this point. Yes, the reported R^2 values are supported by significance testing.

Actions taken: We have now included the adjusted p-values in the revised manuscript to make this information explicit.

Lines *427-432*: Among significant covariates, infant identity, reflecting interindividual variation, had the highest explanatory power (n=984, dbRDA with Bray-Curtis dissimilarity, $R^2 = 0.12$, **adjusted $p < 0.01$**), followed by age ($R^2 = 0.09$, **adjusted $p < 0.01$**), diet ($R^2 = 0.013$, **adjusted $p < 0.01$**), diarrhea occurrence ($R^2 = 0.006$, **adjusted $p < 0.01$**), and antibiotic use ($R^2 = 0.006$, **adjusted $p < 0.05$**) (Fig. 3a, Supplementary Table S6).

<u>Comment 19:</u>

> *Line 384: For the Belgian infants, could the authors provide the sample size? It would be useful to include one or two lines describing this in the Methods section.*

Response 19: We thank the reviewer for this suggestion.

<u>Actions taken:</u> We have included the sample size of the Belgian infant cohort in the Materials and Methods "Study design and sample selection" section of the revised manuscript:

Lines *153-155*: For the comparative analysis with the Belgian infant population, we included 265 samples from the BABEL cohort[10] consisting of 7 infants.

<u>Comment 20:</u>

> *Lines 393-398: I feel these lines on Streptococcus belong in the Discussion rather than the Results section, as there is currently no evidence supporting this claim and remains speculative.*

Response 20: We thank the reviewer for this suggestion. We agree that the interpretation of the observed association is speculative. Accordingly, we have retained the observation that *Streptococcus* was associated with diarrhea episodes in the Results section and moved the interpretation of this finding in the Discussion.

<u>Actions taken:</u> The following sentence in Results section remained as follows:
Lines *444-445*: Interestingly *Streptococcus*, one of the common bacterial genera in healthy early-life samples, was associated with diarrhea episodes in this cohort.

The following lines (highlighted in bold) were added in the <u>Discussion</u> section:

Lines *737-742*: **In addition, *Streptococcus* - one of the common bacterial genera in healthy early-life samples - was associated with diarrhea episodes. We hypothesize that this may be linked to potential enteric viral infections, which could increase the abundance of genera like *Streptococcus*[70,71]. However, as *Streptococcus* is a lactic acid bacterium, its colonization in the infant gut may also be promoted by sustained breastfeeding among other factors. Further research is required to elucidate the role and implications of *Streptococcus* presence in the healthy infant gut within this cohort.**

Comment 21:

> *Lines 424-428: I find this figure largely repetitive of Figure 1A, except that the mother samples have been removed. Could this be integrated?*

Response 21: We thank the reviewer for this suggestion. While both figures present ordinations, they serve different purposes. Figure 1a provides an overview of the overall clustering of all infant and maternal samples. In contrast, Figure 3a accompanies the dbRDA analysis and focuses on the infant samples only, allowing visualization of the metadata covariates associated with compositional variation within this cohort. We therefore believe that keeping these figures separate better reflects the distinct aims of the two analyses, and we have retained them as separate figures.

Actions taken: none

Comment 22:

> *Lines 573-579: This is a very interesting discussion. The idea that breastfeeding cessation is the primary driver of microbiota maturation in Western infants appears to contrast with the findings from the Bangladeshi cohort, where the introduction of solid foods may instead be the major driver of microbiome maturation. Could the authors discuss this apparent difference in greater detail?*

Response 22: We thank the reviewer for highlighting this part of the discussion. To address this comment, we have elaborated more on this section.

Actions taken: The following statements were added (highlighted in bold):

Lines *698-706*: Our observations in a Bangladeshi infant population contradict the idea that breastfeeding cessation is the main driver of microbiota maturation - as seen in western infants[56,60]. Here, we observed gut microbiota maturation over time, reflected by increased alpha diversity and the emergence of more complex taxa, similar to that of the Belgian cohort, despite extended breastfeeding. **Another study conducted in a Bangladeshi infant cohort showed that the introduction of complementary foods was associated with shifts in microbiota composition and suggested that, in populations where breastfeeding continues alongside complementary feeding, dietary transitions may contribute to microbiota maturation beyond the effects of breastfeeding cessation alone[24].**

- [24]Vatanen, T., Ang, Q. Y., Siegwald, L. *et al.* A distinct clade of *Bifidobacterium longum* in the gut of Bangladeshi children thrives during weaning. *Cell* 185, 4280–4297.e12 (2022).

<u>Comment 23:</u>

> *Line 663: Since this is a 16S rRNA gene sequencing study, the results cannot be considered "comprehensive." The term is relative and may overstate the scope of the findings. Could the authors consider using a more appropriate term?*

Response 23: We thank the reviewer for this comment and have rephrased the sentence to avoid using the term "comprehensive".

<u>Actions taken:</u> The following sentence in Conclusion has been modified:
"This study provides one of the most comprehensive views of gut microbiota development in a LMIC country, offering detailed insights into the maturation of the infant gut microbiome in Bangladesh."

And now reads as follows:

Lines *814-816*: This study provides **a densely sampled longitudinal characterization of gut microbiota development in an LMIC setting**, offering detailed insights into the maturation of the infant gut microbiome in Bangladesh.

<u>Comment 24:</u>

> *Lines 667-670: This statement remains too strong and should be toned down.*

Response 24: We agree with the reviewer that this statement should be revised following the modifications made in the interpretation of the gut microbiota changes during the second year of life.

<u>Actions taken:</u> The initial statement in Conclusion was revised:
"We are the first to report potential effects of the COVID-19 pandemic on the early life gut microbiota development in a Bangladeshi infant population, highlighting the need for continued longitudinal monitoring to assess potential long-term impacts."

And is now written as follows:

Lines *818-822*: We are the first to characterize early-life gut microbiota development in a Bangladeshi infant population during COVID-19, providing insights into microbiota dynamics under these unique circumstances and highlighting the need for appropriate study designs and continued longitudinal monitoring to evaluate potential long-term impacts.

Re: mSystems00101-26R2 (Global and local trajectories of infant gut microbiota development in Bangladesh across the COVID-19 pandemic)

Dear Ms. Maria Ioanna Papadaki:

Thank you for the thoughtful replies to the reviewer's comments and the revisions to the manuscript.
Please make sure that the ENA record of the sequence data is made publicly available as soon as possible, as I was unable to locate it.

Your manuscript has been accepted, and I am forwarding it to the ASM production staff for publication. Your paper will first be checked to make sure all elements meet the technical requirements. ASM staff will contact you if anything needs to be revised before copyediting and production can begin. Otherwise, you will be notified when your proofs are ready to be viewed.

Data Availability: ASM policy requires that data be available to the public upon online posting of the article, so please verify all links to sequence records, if present, and make sure that each number retrieves the full record of the data. If a new accession number is not linked or a link is broken, provide production staff with the correct URL for the record. If the accession numbers for new data are not publicly accessible before the expected online posting of the article, publication may be delayed; please contact ASM production staff immediately with the expected release date.

Publication Fees: For information on publication fees and which article types have charges, please visit our website. We have partnered with Copyright Clearance Center (CCC) to collect author charges. If fees apply to your paper, you will receive a message from no-reply@copyright.com with further instructions. For questions related to paying charges through RightsLink, please contact CCC at ASM_Support@copyright.com or toll free at +1-877-622-5543. CCC makes every attempt to respond to all emails within 24 hours.

ASM Membership: Corresponding authors may join or renew ASM membership to obtain discounts on publication fees. Need to upgrade your membership level? Please contact Customer Service at Service@asmusa.org.

Embargo Policy: A press release may be issued as soon as the manuscript is posted on the mSystems Latest Articles webpage. The corresponding author will receive an email with the subject line "ASM Journals Author Services Notification" when the article is available online.

Cover Image Submissions: If you would like to submit a potential Cover Image, please email a file and a short legend to msystems@asmusa.org. Please note that we can only consider images that (i) the authors created or own and (ii) have not been previously published. By submitting, you agree that the image can be used under the same terms as the published article. Image File requirements: TIF/EPS, 7.5 inches wide by 8.25 inches tall (at least 2,250 pixels wide by 2,475 pixels tall), minimum 300 dpi resolution (600 dpi preferred), RGB, and no figure elements, e.g., arrows or panel labels. The legend should be a short description of the image, 1-2 sentences recommended. Please use the ASM Journals Cover Image Sizing Tool (https://journals.asm.org/pb-assets/html/asm-cover-sizing-tool.html) to ensure that your proposed cover image meets our size requirements.

Thank you for submitting your paper to mSystems.

Sincerely,
Evelien Adriaenssens
Editor
mSystems
