# Supplementary material for: Global and local trajectories of infant gut microbiota development in Bangladesh across the COVID-19 pandemic
Source: mSystems. 2026 Sep 1;11(9):e00101-26. doi: 10.1128/msystems.00101-26 (PMC13595919; doi:10.1128/msystems.00101-26)
Supplement: Supplementary file 2 [file msystems.00101-26-s0001.docx]

**Supplementary Figures**

**Supplementary figure S1.** Amplicon Sequence Variants (ASVs) richness accummulation across increasing sample sizes in the BBGUT and BABEL cohorts. (**a**) ASV richness in the BBGUT cohort, assessed using 20 replicates per sample size. The x-axis represents the number of infants included in each iteration (ranging from 1 to 20), while the y-axis indicates the number of unique ASVs detected. Each boxplot displays the distribution of ASV counts for a given sample size. The aim is to evaluate how ASV richness stabilizes as more infants are included, providing insights into ASV saturation within the cohort. (**b**) ASV richness in the BBGUT and BABEL cohorts considering the first year of life only, assessed using 8 replicates per sample size. The same approach was applied, with the x-axis representing the number of infants sampled (ranging from 1 to 20 in BBGUT and 1 to 8 in BABEL), and the y-axis showing the corresponding ASV richness. The fill color differentiates between the two cohorts. This approach allows for an assessment of ASV saturation across varying sample sizes in both cohorts.

**Supplementary figure S2.** Timeline showing the total number samples selected and sequenced per individual infant. Blue circles show the predefined healthy time points (HTP) for which samples were selected when no gastrointestinal disease signs were reported. Grey triagnles represent additional samples selected *ad hoc*, before, during and after disease or vaccination related events (ADHC). In total, 984 samples were selected for the Bangladeshi infant cohort [486 HTP and 498 ADHC).

**Supplementary figure S3.** Control-based quality control across the seven MiSeq runs shows that the maternal *Bacteroides* result is not a sequencing artefact. A control series was included on every 96-well plate of every run, allowing run-to-run technical variation to be measured directly rather than inferred. Run labels are abbreviated (R01 = DI22R01 … R28 = DI22R28). Taxonomy was re-assigned from the raw ASV tables against RDP 19; plant, host and non-prokaryotic sequences were removed before normalization, and libraries were rarefied to even depth within each comparison. **(a)** Bacteroides relative abundance in the positive control, an identical fecal DNA extract sequenced on all seven runs (2 libraries per run; rarefied to 21,149 reads). Each point is one library; the dashed line is the cross-run mean (8.6%). Recovery is consistent across runs (range 7.7–10.6%). Run DI22R28, which carries all 18 maternal samples (pink), gives 8.3%, i.e. z = −0.30 relative to the other six runs, and its library on plate p18, the plate carrying the maternal samples, gives 7.8%. The run and plate that carry the mothers therefore recover *Bacteroides* normally from identical input DNA. Sequencing run did not significantly explain positive-control composition (PERMANOVA on Bray–Curtis dissimilarities, R² = 0.42, p = 0.66, n = 14 libraries). Values are for *Bacteroides* sensu stricto as circumscribed by RDP 19, which places several former *Bacteroides* species in *Phocaeicola.* **(b)** Bray–Curtis dissimilarity between positive-control libraries (technical variation, identical input DNA) and between 200 randomly selected study samples (biological variation). Violins show the full distribution; boxes show the median and interquartile range. Technical variation (median 0.079) is ~6.9-fold smaller than biological variation (median 0.549). **(c)** Reads per library by class (log scale; bar = median). ZymoBIOMICS mock communities are also shown (n=13, median 35,586 reads). Negative controls (DNA-extraction and PCR blanks) carry a median of 253 reads, ~0.5% of the median study-sample depth (47,537 reads), indicating low background. The single-organism *Runella slithyformis* control was 99.96–99.99% on target across runs (off-target 0.006–0.040%), indicating negligible index hopping or cross-talk between libraries.

**Supplementary figure S4.** **(a)** Sankey diagram illustrating taxonomic changes in the BABEL cohort data when using the updated taxonomic classifications from RDP training set 19 (rdp_train_set_19) compared to the previously used RDP set 16 used for the published BABEL data in 2021 (Beller et al. 2021). In total, ~39% of the originally identified genera (24 out of 61) exhibited taxonomic revisions under the updated classification scheme used in the latest RDP set. Note that the latest reliesed RDP training set includes more than 600 new genera and 2500 species since version 18 (Wang et al. 2024). **(b)** Alluvial plot depicting the redistribution of samples from the BABEL dataset, which were previously classified into three maturation stages (n = 7 BABEL infants; 1 excluded as an outlier [DMM_BABEL2021]), now clustering into two maturation stages (DMM_BABEL2025). While taxonomic updates play a key role in the shifts observed in the Dirichlet Multinomial Mixture (DMM) analysis, minor variations also emerge from stochastic factors introduced during data processing.These differences likely resulted from slight variations in the reproduction of the decontamination and rarefaction steps, performed to align with the BBGUT data processing pipeline for replication purposes. Specifically, these methodological factors led to differences in 10 % of the ASV sequences (23/197) between the old and new taxonomy tables, ultimately influencing the inferred maturation stages in the current DMM analysis.

**Supplementary figure S5**. **(a)** Empirical cumulative distribution function (ECDF) plot and showing the cumulative proportion of samples with *Segatella* relative abundance, with a dashed vertical line at 0.25 abundance, marking the threshold we define as high Segatella abundance (HSA), where 20% of the samples are above this value. **(b)** Histogram illustrating the distribution of *Segatella* relative abundance across the infant fecal samples. A discontinuity in the histogram further supported the selection of the 0.25 abundance threshold as a definition for HSA samples.

**Supplementary figure S6.** (**a**) Timing of transitions between GM Maturation stages A to B and B to C. Each boxplot represents the distribution of transition age for the respective transition type, with the y-axis indicating the day of transition. Individual data points represent transition events for each individual, with colors distinguishing different infants. (**b**) Alpha diversity (observed and Shannon diversity) measures across age categories. Each boxplot represents the distribution of diversity values within an age group (shown in 3-month bins on the x-axis). We assessed the significance of the increase in diversity over time for infants using a linear mixed-effects model accounting for dependence resulting from repeated measurements of the same infant using a random effect (p< 2.2e-16 [observed diversity], p< 2.2e-16 [shannon diversity]). Similarly, we also used a linear mixed-effects model to compare the alpha diversity between the last trimester of the second year (21-24 months) and the maternal samples accounting for pairing of the data between mothers and their infants (p= 1.241e-06 [observed diversity], p=0.09876 [shannon diversity]).

**Supplementary figure S7.** Relative abundance of most common bacterial genera detected in maternal samples. The stacked bars show the relative abundance of the top 15 bacterial genera across 18 maternal samples. Each bar represents a maternal sample, with different genera color-coded according to their taxonomical annotation. The height of each colored segment within a bar reflects the relative abundance of the corresponding genus in that sample. At the top, the percentage of water content measured for each maternal sample is displayed as calculated after sample liophylization.

**Supplementary figure S8. (a)** Differential abandance analysis of the most common bacterial genera found in the BBGUT and BABEL cohorts. Only genera with a statistically significant difference (Wilcoxon test, adjusted *p* <0.05) are displayed in the plot. **(b)** Longitudinal trends in the median relative abundance of most common bacterial genera detected in the BBGUT and BABEL cohorts over the first year of life. Only genera with a relative abundance of at least 0.5% in at least one sample were included. Lines represent cohort-specific trends, with points indicating median values for each month. Certain genera were excluded from each cohorts as described in Fig. 2(a), to focus on relevant comparisons.

**Supplementary figure S9**. Month-specific analysis of alpha diversity (observed and Shannon diversity) measures between cohorts. Samples are shown as individual points with boxplots representing the distribution within each cohort. Statistical comparisons between cohorts were performed for each month using Wilcoxon rank-sum tests, with p-values adjusted for multiple testing using the Benjamini–Hochberg method. Significant differences were observed only at month 0 (first 10 days of life) for observed richness (adjusted *p* = 0.003) and Shannon diversity (adjusted *p* = 0.005). No significant differences were detected between cohorts at any subsequent month (adjusted *p* > 0.05).

**Supplementary figure S10**. **(a)** Order of appearance for the top 15 most abundant genera found in the BABEL (left) and **(b)** BBGUT (right) cohorts. Genera represent 97% and 96% of the total read abundances respectively. Genera are ordered according to their first appearance (presence defined as relative abundance >=0.5%) during the first year of life. The bacterial generadepicted further grouped and colored according to their Phylum classification.

**Supplementary figure S11**. (**a**) Progression of GMM stages over time for all samples across infants of the BBGUT cohort (n=984). Time points for which the microbiota reverts to a previous maturation stage are highlighted with larger dots. Regression to a lower GMM stage is considered following at least two samples of the next stage during the first year and following one sample of the next stage in the second year. (**b**) Variation in maturation scores across time for all samples of each infant. Maturation scores were determined by averaging the ranked order of genera present in each sample (presence threshold: relative abundance >=0.5 %). The black line represents a quadratic regression model with a 95% confidence interval. Larger dots represent maturation scores lower than the confidence interval. Here, for both plots larger dots aim to highlight only samples (n=31) that display both regression to a previous maturation stage and a low maturation score.

**Supplementary figure S12.** Comparison of alpha diversity metrics before and during setback events in infants from the BBGUT cohort**.** Paired boxplots depict observed richness (left) and Shannon diversity (right) in samples collected during setback events (pink) and the most recent healthy time points prior to setback (teal). Samples were paired within individuals (n = 33 setback events). Significant decreases in both observed richness (Wilcoxon signed-rank test, *p* = 5.5 × 10⁻⁶) and Shannon diversity (Wilcoxon signed-rank test, *p* = 1.9 × 10⁻⁴) were detected during setbacks compared to preceding healthy samples.

**Supplementary figure S13.** Boxplots showing two-month fluctuations in genus-level bacterial abundances across early (months 13–16) and late (months 17–24) age windows. Normalized median abundance changes at the genus level were calculated using all valid pairs of equidistant time points spaced two months apart, for each child and bacterial genus, across the 9–24 month age range. For each child and genus, the normalized change was defined as the difference between the two median abundances divided by their average. Sample pairs were categorized into three period groups: ***E–E*** (both time points during the **early age window** period, months 9–16), ***E–L*** (crossing from **early to late age window**, months 15–18), and ***L–L*** (both time points during the **late age window** period, months 17–24). Statistical differences between groups were assessed using linear mixed-effects models fitted separately for each genus, with fixed effects for period group and age, and random intercepts for each child. Genera with significant differences between period comparisons (*p* < 0.05) are highlighted in the figure.

**Supplementary figure S14.** Stringency index for Bangladesh during the first year of the COVID-19 pandemic. Data retrieved from <https://data.humdata.org/dataset/oxford-covid-19-government-response-tracker>. Data source: Blavatnik School of Government, University of Oxford (2023) – with minor processing by Our World in Data.

 **Supplementary figure S15. (a**) Barplot showing distribution of *Segatella* prevalence across different gut microbiota maturation (GMM) stages and maternal samples in different sample groups. Groups represent (i) all infant and maternal samples (n= 1002), (ii) only healthy infant samples (n=486) and (iii) only disease-associated samples (n= 420). For visualization purposes, maternal samples which are also assigned the maturation stage C, were named C_m to distinguish between infant and maternal. Comparing the prevalence of *Segatella* between disease- and healthy-associated samples, a statistically significant difference was observed only in maturation stage C (Fisher’s exact test; stage A: *p* = 0.628; stage B: *p* = 0.628; stage C: *p* = 3.24 × 10⁻⁷). **(b)** Principal Coordinate Analysis (PCoA) of infant and maternal gut microbiome samples, illustrating the variation in *Segatella* relative abundance across samples. Samples are color-coded according to the abundance, with a gradient ranging from low (blue) to high (red). Fecal sample types are distinguished by different shapes (dots for infants and triangles for maternal samples).

**Supplementary figure S16.** Boxplots showing the distribution of median abundances for the different *Segatella* ASVs present in HSA samples associated with healthy time points (healthy) and disease. For the differential abundance analysis, only ASVs with sufficient paired observations were included for comparison using the Wilcoxon signed-rank test. No statistically significant differences were found after correction for multiple testing.While certain ASVs, such as ASV10 and ASV11, suggest potential differences in abundance between groups, we can not draw firm conclusions due to the limited sample size.

**Supplementary figure S17.** Boxplots showing the relative abundance of bacterial genera that differ significantly between the HSA and HTPcontrol (samples corresponding to the most recent HTP sample prior to the HSA) sample groups (paired Wilcoxon signed-rank tests, *p* < 0.001, adjusted for multiple comparisons using the Benjamini–Hochberg (BH) method).
