# Supplementary material for: Global and local trajectories of infant gut microbiota development in Bangladesh across the COVID-19 pandemic
Source: mSystems. 2026 Sep 1;11(9):e00101-26. doi: 10.1128/msystems.00101-26 (PMC13595919; doi:10.1128/msystems.00101-26)
Supplement: Supplementary file 3 [file msystems.00101-26-s0002.docx]

Study design

Thirty pregnant women were enrolled at the International Centre for Diarrheal Disease Research, Bangladesh (icddr,b) for the purposes of this study. All participating women were healthy and in the third trimester of their pregnancy at the time of enrollment. Individuals younger than 18 years of age or with major underlying conditions such as cancer, uncontrolled hypertension, or mental illness were excluded from participation. At the first household visit, information on birth characteristics (mode and place of delivery, gestational age) and overall maternal health during the final trimester was collected to confirm that no serious adverse events had occurred. No additional maternal health data were collected on the day of delivery, or in the postpartum period.

Longitudinal metadata for this study was collected exclusively for the infants and included demographic information (birth mode/birth place/household composition), medical history information (vaccination/antibiotics/medication/disease occurrence) as well as dietary details (breastfeeding/formula feeding/solid food) (Supplementary Table S1). All metadata was collected using structured questionnaires administered to the mothers by trained field staff during the household visits for sample collection and transportation. These visits were conducted as frequently as sampling occurred, and information was obtained via maternal report using standardized questionnaire responses, covering both the day of sample collection and the interval since the previous visit. Specifically, medical history questions asked which vaccines, antibiotics, or other medications the infant had received, including timing, duration, and reason for administration. For disease occurrence, mothers reported any illnesses their child had experienced or was experiencing since the previous visit. Dietary questions captured whether breastfeeding occurred and its frequency, the use of formula, and whether the infant had begun consuming solid foods. Most infants exclusively breastfed during the first six months on average, with continued breastfeeding alongside complementary food throughout the second year.

In total 5,082 samples were collected throughout the sampling period. Fresh samples were collected by the mothers in pre-labeled tubes and transported on the same day to the International Centre for Diarrheal Disease Research, Bangladesh (icddr,b) by trained field staff. During the COVID-19 pandemic lockdown in Bangladesh (March-November 2020), samples were stored at -20°C in freezers at the participants’ homes and were collected and transported once a week or biweekly, while metadata was collected over the phone. Upon delivery to the Virology laboratory at the International Centre for Diarrheal Disease Research, Bangladesh (icddr,b), all samples were aliquoted in capped O-ring 2mL tubes and stored in the Virology laboratory at the International Centre for Diarrheal Disease Research, Bangladesh (icddr,b) at -80°C until shipment to the Rega Institute laboratory of Viral Metagenomics in Belgium. Samples were shipped on dry ice in two batches (first year samples shipped in December 2019, second year samples shipped in January 2021) and stored at -80°C until further processing. The STORMS checklist for the current study can be accessed at <https://github.com/Matthijnssenslab/Bangladesh_InfGut_16S>.

Sample selection

Infant selection for sequencing was done after collection of the first year samples. Due to gaps in sampling and metadata information for some infants, only 20 out of the 30 enrolled infants were selected for this study. Due to additional dropouts and sampling gaps during the second year, samples and information were only available for 16 of the 20 infants. To assess whether our sample size was sufficient for this study, we measured amplicon sequence variant (ASV) richness accumulation across increasing sample sizes (Supplementary Fig. S1a, b).

For the characterization of the longitudinal development of the healthy gut microbiota, we selected approximately 33 samples per infant at predefined time points without reports for antibiotic intake or gastrointestinal disease (~21 samples during the first year, ~12 during the second year). To facilitate comparisons with the Belgian cohort, samples around predefined time points (i.e., samples representing healthy time points or HTP samples) were selected around the following days for each infant:

**a)** Year 1: 0, 3 7, 10, 15, 21, 30, 45, 60, 75, 90, 105, 120, 150, 180, 210, 240, 270, 300, 330, and 360 (as described previously^1^)

**b)** Year 2: 390, 420, 450, 480, 510, 540, 570, 600, 630, 660, 690, 720
When samples selected for this category were not available at a given predefined time point (e.g. due to sampling limitations), the next available sample was selected within a window of 2-5 days before/after the predefined time point. In the event that no samples were available within the defined time window, no sample was selected. Samples were considered as HTP if they were collected at least 6 days after an antibiotic event during year one and at least 3 days after an antibiotic event during year 2. If clinical signs of gastrointestinal disease (without antibiotic administration) occurred at the foreseen sample selection time point, a sample was considered HTP if collected at least 4 days later. In total, 486 (out of 984) samples were selected as HTP (Supplementary Fig. S2).

To assess the effect of external events on the early gut microbiota development we selected 20-30 additional samples per infant associated with specific life events during the first year of life. In this work, these *ad hoc* collected samples (i. e., ADHC samples) were chosen before (closest day before onset), during (one or more samples depending on event duration) and after (up to 10 days post-event) occurrences of particular events: gastrointestinal disease and/or antibiotic intake, antiparasitic/antifungal medication or vaccination events. For reported gastrointestinal clinical signs (e.g., abdominal swelling/abdominal discomfort, constipation), events lasting more than two days prompted selection of multiple surrounding samples. Events lasting two days or less led to the consideration of the closest subsequent sample as a HTP sample following the criteria described in the previous paragraph. Medication with non-antibiotic and/or non-antifungal drugs was not considered as an external event due to their high administration frequency. Nevertheless, non-antibiotic and/or non-antifungal medication with potential gastrointestinal effects were considered in the forward dbRDA analysis (see Statistical analysis) and did not significantly explain compositional variation. Given the less dense sampling that took place in the second year of life, second year samples were not assigned to external events unless they coincided with a gastrointestinal disease or medication event as described above. In total, 498 (out of the 984) ADHC infant samples, representing 20 individuals, were selected for this part of the study (Supplementary Fig. S2).

To explore the gut microbiota development during the second year of life, we divided the data into two calendar-based periods according to the recorded sample collection date. Bangladesh declared a nationwide general holiday, with movement restrictions, from 26 March 2020. Samples collected before and on or after that date were classified in two age periods: the months 13-16 period, corresponding to samples collected before the lockdown was declared and the months 17-24 period, corresponding to samples collected after this date. All samples fell within their respective age-period classifications, with the exception of one (I01_151), which, although collected at month 17, was obtained before the lockdown was declared and was therefore included in the months 13–16 period. The comparison was restricted to healthy (HTP) infant samples from the second year of life, giving 52 samples in the months 13-16 period (16 November 2019 – 25 March 2020) and 106 in the months 17-24 period (31 March – 19 November 2020, infant age 17–24 months) from 16 infants.

Fecal microbiota profiling

All fecal samples were further aliquoted at the Rega Institute (Belgium) and weighed (150–200 mg) while maintained in a frozen state to ensure accurate sample input prior DNA extraction. For this part of the analysis, one aliquot was used for the laboratory analysis, and one was kept as backup at -80°C. Bacterial DNA extraction followed a modification of a published protocol^2^. Briefly, DNA was extracted from the frozen fecal aliquots using the MagAttract PowerMicrobiome DNA/RNA kit (Qiagen) on the TECAN extraction platform, adding a 10-minute heating step at 90°C after vortexing, and omitting the steps of DNA removal.

16S rRNA amplification and sequencing followed a method previously described^3^, where extracted DNA was amplified in triplicate using standard primers [515F(5'-GTGYCAGCMGCCGCGGTAA-3') and 806R(5'-GGACTACNVGGGTWTCTAAT-3')] targeting the V4 region of the 16S rRNA gene. To create dually barcoded libraries, these primers were modified to include index sequences between each primer and the Illumina adaptor sequences. All samples were randomized at every part of the laboratory processing, beginning with aliquoting and continuing through the seven Illumina MiSeq sequencing runs, each of which comprised two 96-well plates. Each run included samples from 17–20 of the 20 infants. Two departures from complete randomization occurred: all 18 maternal samples were sequenced in a single run, and because year-1 and year-2 samples were shipped in two batches, most year-2 samples (154/175 analyzed samples; 88%) were sequenced in one run, resulting in confounding between study year and sequencing run (described in more detail under STORMS item 7.7). Negative controls (DNA extraction negative control and PCR negative control) included along the whole process. Positive controls, specifically: DNA from a fecal sample analyzed previously, a *Runella slithyformis* control and a commercial mock community (ZymoBIOMICS) were also used to assess technical variability across the entire workflow.

To evaluate whether the maternal *Bacteroides* abundance results could be explained by sequencing batch effects, we analyzed the control series included on every plate of each MiSeq run. The positive control, comprising identical fecal DNA sequenced across all seven runs, showed consistent *Bacteroides* recovery (mean 8.6%, range 7.7–10.6%), including in the run containing the maternal samples (8.3%), indicating normal recovery from identical input DNA. Technical variation among positive-control libraries was substantially lower than biological variation among study samples (median Bray–Curtis dissimilarity 0.079 versus 0.549), and sequencing run did not significantly influence positive-control composition (PERMANOVA, R² = 0.42, *p* = 0.66) (Supplementary Fig. S3). In addition, the *Runella slithyformis* control was 99.96–99.99% on target across runs, and negative controls contained only ~0.5% of the median study-sample read depth. Mock communities showed consistent genus-level taxonomic profiles across sequencing runs (median Pearson correlation = 0.94; median Bray–Curtis dissimilarity = 0.10, 99.30–100.00% on-target), supporting the reproducibility of the sequencing and analysis workflow. Together, these results indicate that the observed microbiota patterns, including the maternal profile, primarily represent biological signals rather than technical variability or sequencing noise.

Statistical analyses

**Dirichlet multinomial mixture modeling (DMM) for identification of bacterial communities**

Community typing was performed by applying a Dirichlet multinomial mixture modeling (DMM) approach^4^ to the 16S rRNA gene dataset in order to explore the presence of bacterial sub-communities within the Bangladeshi infant cohort. For this purpose, we used the *DirichletMultinomial* R package^5^ (v1.40.0) on the rarefied table agglomerated at the genus level. The best number of clusters was determined based on the Bayesian information criterion (BIC) and every sample was assigned to one (Supplementary Table S2).

**Determining maturation setbacks**

“Maturation setbacks” were defined by two criteria: regression to a lower gut microbiota maturation (GMM) stage, as determined by the DMM analysis, and a decrease in the maturation score. Maturation scores per sample were calculated as described in Beller and colleagues by computing the average ranks of the genera, with weights assigned based on the presence or absence of each specific genus^1^. Maturation curves were computed for each infant by applying a quadratic fit on maturation scores over time. During the first year of life, a regression was defined as a return to an earlier maturation stage (e.g., from stage B to A) following at least two consecutive samples classified in the more advanced stage. In the second year, where one sample per infant per month was analyzed, a regression was defined as any return to an earlier stage, even if it followed only a single sample in the subsequent stage. To confirm a maturation setback, both a regression to an earlier maturation stage and a maturation score below the curve’s confidence interval were required.

**Order of appearance of most abundant bacterial genera**

The most abundant genera in the cohort were defined as being present in more than 25% of the infants. Genera were then ranked based on the day in which they first appeared with a relative abundance exceeding 0.5%. Rankings were assessed using the Kendall W test with 10,000 permutations, applying the kendall.w function from the R *synchrony* package^6^ (v0.3.8). The final sequence of appearance was established by ordering the median ranks for each infant.

**Diversity metrics and multivariate analysis of metadata effects on the microbiota**

Alpha diversity (observed genus richness and Shannon diversity) and beta diversity (Bray-Curtis dissimilarity) metrics were calculated using the *microbiome*^7^ (v1.20.0) and *phyloseq*^8^ (v1.42.0) packages and genus-level ordinations were visualized using Principal-coordinate analysis (PcoA) computed from Bray-Curtis dissimilarities. To determine the contribution of metadata covariates on microbiota variation, we conducted a univariate distance-based redundancy analysis (dbRDA) at the genus level using Bray-Curtis distances and the capscale function from the *vegan*^9^ R package (v2.6-4). Significant covariates were identified and then refined with forward model selection (stepwise dbRDA) via the ordiR2step function in *vegan* with 1000 permutations per step, providing a nonredundant cumulative assessment of metadata contributions. The envfit function from the *vegan* package was used to fit metadata variables onto the PCoA ordination, with effect sizes shown as arrows. To examine the influence of metadata on specific genera, an analogous approach was used: the community was narrowed to focus on each of the top 15 genera, with dbRDA performed on Euclidean distances based on genus abundance, followed by forward model selection, while controlling for infant ID.

**Samples with high *Segatella* abundance (HSA)**

To determine the threshold of which samples were considered to have high *Segatella* abundances (HSA) an empirical approach was followed. First, only samples following the first day of the *Segatella* appearance were considered. We then examined Segatella abundance in our data and visually set a high-abundance threshold using a histogram and empirical cumulative distribution function (Supplementary Fig. S5). Thus, high *Segatella* abundance was defined as a relative abundance higher than 25% in a given sample. In case multiple HSA samples were identified in a single disease-associated event, then the sample with the highest *Segatella* abundance was selected for further analysis.

We performed a within-individual paired analysis to assess compositional changes associated with high *Segatella* abundance (HSA) events. For each HSA representative sample, the closest preceding healthy time point (HTP) sample from the same individual was selected as a matched reference, and paired differences in bacterial genus relative abundance between HSA and HTP samples were tested using Wilcoxon signed-rank tests with Benjamini–Hochberg correction for multiple comparisons.

References

1.	Beller, L. *et al.* Successional stages in infant gut Microbiota maturation. *MBio* **12**, e0185721 (2021).

2.	Falony, G. *et al.* Population-level analysis of gut microbiome variation. *Science* **352**, 560–564 (2016).

3.	Tito, R. Y. *et al.* Brief report: Dialister as a microbial marker of disease activity in spondyloarthritis. *Arthritis Rheumatol.* **69**, 114–121 (2017).

4.	Holmes, I., Harris, K. & Quince, C. Dirichlet multinomial mixtures: generative models for microbial metagenomics. *PLoS One* **7**, e30126 (2012).

5.	Morgan, M. DirichletMultinomial: Dirichlet-Multinomial Mixture Model Machine Learning for Microbiome Data. R package version 1.47.2. (2024).

6.	Gouhier, T. C. & Guichard, F. Synchrony: quantifying variability in space and time. *Methods in Ecology and Evolution* **5**, 524–533 (2014).

7.	Lahti, L. & al., S. S. et. Tools for microbiome analysis in R. Microbiome package version 1.23.1. (2017).

8.	McMurdie, P. J. & Holmes, S. phyloseq: an R package for reproducible interactive analysis and graphics of microbiome census data. *PLoS One* **8**, e61217 (2013).

9.	Oksanen, J. *et al.* vegan: Community Ecology Package. R package version 2.7-0. (2025).
